# Microcin determinants are associated with B2 phylogroup of human fecal *Escherichia coli* isolates

**DOI:** 10.1002/mbo3.345

**Published:** 2016-03-14

**Authors:** Lenka Micenková, Juraj Bosák, Barbora Štaudová, Darina Kohoutová, Darina Čejková, Vladana Woznicová, Martin Vrba, Alena Ševčíková, Jan Bureš, David Šmajs

**Affiliations:** ^1^ Department of BiologyFaculty of MedicineMasaryk UniversityKamenice 5Building A6625 00BrnoCzech Republic; ^2^ 2nd Department of Internal Medicine ‐ GastroenterologyCharles University in PragueFaculty of Medicine at Hradec KrálovéUniversity Teaching HospitalSokolská 581500 05Hradec KrálovéCzech Republic; ^3^ Department of ImmunologyVeterinary Research InstituteHudcova 70621 00BrnoCzech Republic; ^4^ Department of MicrobiologyFaculty of MedicineMasaryk University and St. Anne's University HospitalPekarˇská 53656 91BrnoCzech Republic; ^5^ Department of Clinical MicrobiologyFaculty Hospital BrnoJihlavská 20625 00BrnoCzech Republic

**Keywords:** Bacteriocin, colicin, *E. coli*, microcin, phylogroup.

## Abstract

*Escherichia coli* strains are classified into four main phylogenetic groups (A, B1, B2, and D) and strains of these phylogroups differ in a number of characteristics. This study tested whether human fecal *E. coli* isolates belonging to different phylogroups differ in prevalence of bacteriocinogenic isolates and prevalence of individual bacteriocinogenic determinants. A set of 1283 fecal *E. coli* isolates from patients with different diseases was tested for the presence of DNA regions allowing classification into *E. coli* phylogroups and for the ability to produce bacteriocins (23 colicins and 7 microcins). Of the isolates tested, the most common was phylogroup B2 (38.3%) followed by phylogroups A (28.3%), D (26.3%) and B1 (7.2%). Altogether, 695 bacteriocin producers were identified representing 54.2% of all tested isolates. The highest prevalence of bacteriocin producers was found in group B2 (60.3%) and the lowest in group B1 (44.6%). Determinants encoding colicins E1, Ia, and microcin mV were most common in phylogroup A, determinants encoding microcins mM and mH47 were most common in phylogroup B2, and determinant encoding mB17 was most common in phylogroup D. The highest prevalence of bacteriocinogeny was found in phylogroup B2, suggesting that bacteriocinogeny and especially the synthesis of microcins was associated with virulent and resident *E. coli* strains.

## Introduction

*Escherichia coli* is a commensal in the human gastrointestinal tract, and at the same time, one of the most frequent pathogens. *E. coli* represents a rather heterogeneous bacterial species, differing in a number of important characteristics. *E. coli* strains can be classified into four main phylogenetic groups A, B1, B2, and D (Clermont et al. 2000). *E. coli* strains belonging to different phylogroups differ in a number of characteristics including genome size (Bergthorsson and Ochman 1998), number of pathogenic strains (Boyd and Hartl 1998; Picard et al. 1999; Johnson and Stell 2000), virulence (Dobrindt et al. 2003), antibiotic‐resistance profiles (Coque et al. 2008), and abilities to exploit sugars and ecological niches (Gordon and Cowling 2003). As shown by Gordon and Cowling (2003), the relative abundance of *E. coli* phylogroups in mammals depends on climate, host diet, and body mass. The phylogroup combination of A/B1 is common in nonhuman mammals, the phylogroup combination of A/B2 is most prevalent in humans (Escobar‐Páramo et al. 2006) and the subgroup of phylogroup B2 and B2_3_, has only been found in humans (Carlos et al. 2010).

In humans, resident and transient commensal *E. coli* strains can be distinguished based on their ability to persist in the intestines. The persistence of *E. coli* strains in human intestines is associated with the presence of several virulence determinants (P‐fimbriae, capsule, aerobactin production) (Adlerberth et al. 1998; Nowrouzian et al. 2001, 2005). In general, *E. coli* strains in phylogenetic group B2 carry more virulence factors than strains belonging to phylogroups A, B1, and D (Picard et al. 1999; Nowrouzian et al. 2005). A relationship between virulence and residency may exists since *E. coli* strains in phylogroup B2 are more often resident strains than transient strains (Zhang et al. 2002; Gordon et al. 2005; Nowrouzian et al. 2005). The presence of *E. coli* strains belonging to phylogroups B2 and D have also been found to be associated with advanced colorectal neoplasia (Kohoutová et al. 2014). In addition, extraintestinal pathogenic *E. coli* strains are the most often in phylogroups B2 and D (Picard et al. 1999; Johnson and Russo 2002).

*Escherichia coli* strains are known to synthesize two types of bacteriocins, colicins and microcins. While colicins are plasmid‐encoded toxic exoproteins with molecular masses ranging from 29 to 75 kDa, microcins are oligopeptides with molecular masses up to 12 kDa (Braun et al. 1994; Šmarda and Šmajs 1998; Cascales et al. 2007). Unlike colicins, microcins are often post‐translationally modified and are exported from the producer bacteria (Cascales et al. 2007; Duquesne et al. 2007). The ecological role and molecular evolution of bacteriocinogeny remains unclear, however, the synthesis of bacteriocins may have both defensive and invasive functions within microbial communities (Riley and Wertz 2002).

Association of *E. coli* phylogroups and bacteriocinogeny has been tested in several previous publications, which produced different results. Gordon and O'Brien (2006) tested a set of 266 fecal *E. coli* strains for the presence of 18 bacteriocin types and failed to find significant differences in the frequency of bacteriocinogeny between different *E. coli* phylogroups. They also failed to find differences in the prevalence of bacteriocin determinants among the phylogroups. In a study on human fecal and uropathogenic strains (411 and 361 strains, respectively), the prevalence of colicinogenic strains was higher in phylogroups A and D compared to phylogroup B2, while group B2 had a higher prevalence of microcin‐encoding strains compared to strains in group A or D (Šmajs et al. 2010). A study by Budicˇ et al. (2011) revealed increased bacteriocinogeny in the phylogroup B2 among 105 uroseptic strains. In addition, an association between production of several bacteriocins and virulence determinants has been found in a number of studies (Azpiroz et al. 2009; Šmajs et al. 2010; Budicˇ et al. 2011; Petkovšek et al. 2012; Micenková et al. 2014).

In this communication, we studied the prevalence of bacteriocin production and the prevalence of bacteriocin determinants (23 colicin and 7 microcin types), relative to *E. coli* phylogroups (A, B1, B2, and D), in a large set of 1283 fecal *E. coli* isolates of human origin. The objective of the study was to determine if there is a relationship between *E. coli* phylogenetic group and production of bacteriocins, including the frequency of bacteriocin types.

## Materials and Methods

### Bacterial strains

*Escherichia coli* isolates of human fecal origin analyzed in this study (*n *=* *1283) were isolated from patients at two university hospitals in Brno (*n *=* *1181) and one in Hradec Králové, Czech Republic (*n *=* *102), between the years 2007 and 2012. In this study, predominant *E. coli* isolates (*n *=* *102) were selected from the set of *E. coli* strains previously described by Kohoutová et al. (2014). A single *E. coli* isolate was used from one patient.

The patients from which *E. coli* isolates were isolated were indicated for cultivation of intestinal flora for different reasons including infectious and parasitic diseases (*n *=* *471); neoplasms (*n *=* *152); blood diseases (*n *=* *10); endocrine, nutritional and metabolic diseases (*n *=* *107); mental and behavioral disorders (*n *=* *5); diseases of the nervous system (*n *=* *12); diseases of the circulatory system (*n *=* *33); diseases of the respiratory system (*n *=* *22); diseases of the digestive system (*n *=* *216); diseases of the skin and subcutaneous tissue (*n *=* *11); diseases of the musculoskeletal system and connective tissue (*n *=* *10); diseases of the genitourinary system (*n *=* *16); pregnancy, childbirth, and the puerperium (*n *=* *5); congenital malformations, deformations, and chromosomal abnormalities (*n *=* *1); symptoms, signs, and abnormal clinical and laboratory findings, not elsewhere classified (*n *=* *100); injury, poisoning and certain other consequences of external causes (*n *=* *10); and factors influencing health status and contact with health services (*n *=* *102). An International Statistical Classification of Diseases and Related Health Problems, 10th Revision (ICD‐10)‐2015‐WHO Version for 2015, was used for the classification of diseases. Detailed characteristics of all clinical isolates are summarized in Table S1.

*Escherichia coli* isolates were identified using the biochemical ENTEROtest16 (Erba Lachema, Brno, Czech Republic). All clinical samples were collected after patients gave written informed consent regarding participation in the study and for their samples to be used for research. The study was approved by the Joint Ethics Committee (Charles University in Prague, Faculty of Medicine at Hradec Králové & University Teaching Hospital Hradec Králové) and the ethics committee of the Faculty of Medicine, Masaryk University, Czech Republic.

Six different indicator strains (*E. coli* K12‐Row, C6 (*ϕ*), B1, P400, S40, and *Shigella sonnei* 17) were used to detect bacteriocinogeny on agar plates (Šmajs et al. 2010; Micenková et al. 2014). For PCR screening, control colicin producers were as follows (*E. coli* BZB2101pColA ‐ CA31, BZB2102 pColB ‐ K260, BZB2103 pColD ‐ CA23, BZB2107 pColE4 ‐ CT9, BZB2108 pColE5 ‐ 099, BZB2150 pColE6 ‐ CT14, BZB2120 pColE7 ‐ K317, BZB2279 pColIa ‐ CA53, BZB2202 ColIb ‐ P9, BZB2116 pColK ‐ K235, PAP1 pColM ‐ BZBNC22, BZB2123 pColN ‐ 284 (original source: A. P. Pugsley), *E. coli* 189BM pColE2 ‐ P9 (B. A. D. Stocker), *E. coli* 385/80 pColE1, pColV (H. Lhotová), *E. coli* 185M4 pColE3 ‐ CA38 (P. Fredericq), *E. coli* W3110 pColE8, W3110 pColE9 (J. R. James), *E. coli* K‐12 pColS4 (D. Šmajs), *S. boydii* M592 (serovar 8) pColU (V. Horák), *E. coli* K339 pColY (D. Friedman), *Shigella sonnei* (colicinotype 7) pColJs (J. Šmarda), and *E. coli* pCol5 and *E. coli* pCol10 (H. Pilsl). As microcin control producers, the following bacterial strains were used: *E. coli* 449/82 pColX (microcin B17); *E. coli* 313/66 pColG (microcin H47); *E. coli* 363/79 pColV (microcin V, original source: H. Lhotová); *E. coli* TOP10F’ pDS601 (microcin C7); *E. coli* D55/1 (microcin J25); *E. coli* B1239 (microcin L, D. Šmajs) were used as positive controls for PCR reactions (Šmajs et al. 2010; Micenková et al. 2014).

### Phylogenetic analysis of *E. coli* isolates

A previously described, the triplex PCR method, based on the combination of *chuA* and *yjaA* genes and the TSPE4.C2 DNA fragment, was used to assign *E. coli* isolates to one of the four main phylogenetic groups (A, B1, B2, and D) (Clermont et al. 2000).

In addition, we have analyzed a set of randomly selected 96 *E. coli* isolates (B776 – B867) with a new Clermont et al. (2013) phylotyping method. Modified *chuA*,* yjaA*, and TSPE4.C2 DNA primers were used and an additional gene, *arpA*, was detected. *E. coli* isolates were assigned to the phylogenetic groups A, B1, B2, D, C, E, F, and clade I (Clermont et al. 2013).

### Bacteriocin detection

TY agar plates (containing yeast extract (Hi‐Media, Mumbai, India) 5 g/L, tryptone (Hi‐Media) 8 g/L, sodium chloride 5 g/L, and a 1.5% (w/v) of agar (Hi‐Media) were inoculated using the needle stab technique and plates were incubated at 37°C for 48 h. The bacteria were then killed using chloroform vapors (30 min) and each plate was then overlaid with a thin layer of soft agar (TY agar, 0.7% (w/v)) containing 10^7^ cells/mL of an indicator strain (*E. coli* K12‐Row, C6 (*ϕ*), B1, P400, S40 and *Shigella sonnei* 17). The plates were then incubated at 37°C overnight. All 1283 *E. coli* isolates of clinical origin were tested against all six indicators. Only zones of at least 1.5 mm width were considered as bacteriocin inhibition zones. In the case of producer isolates, bacteriocin type was further identified (Šmajs et al. 2010; Micenková et al. 2014), when detection of the 23 colicin (A, B, D, E1, E2‐9, Ia, Ib, Js, K, L, M, N, S4, U, Y and 5/10) and seven microcin (mH47, mM, mB17, mC7, mJ25, mL, and mV) genes was carried out. Isolated genomic DNA (using DNAzol reagent, Invitrogen, Carlsbad, CA, USA according to the manufacturer's protocol) was diluted 100‐fold in sterile distilled water. Alternatively, the colony PCR method was used (one bacterial colony of each bacteriocin producer was resuspended in 100 *μ*L of sterile distilled water and 1 *μ*L of this suspension was added to the PCR mix). A list of primers is shown in Table S2. PCR method conditions were 94°C (2 min; 5 min for colony PCR); 94°C (30 sec), 60°C (30 sec), 72°C (1 min), 30 cycles; and 72°C (7 min).

Because microcins mH47 and mM are sensitive to chloroform vapors (Patzer et al. 2003), all *E. coli* isolates were tested for the presence of microcin M and H47‐encoding genes using the PCR method. PCR products of related bacteriocin types (colicins E2‐9, Ia‐Ib, U‐Y) were sequenced using dideoxy chain terminator sequencing with amplification primers (Table S2) and sequences were analyzed using Lasergene software (DNASTAR, Inc., Madison, WI).

### Statistical analyses

The statistical analyses of the prevalence bacteriocin determinants and of phylogenetic groups used standard methods derived from the binomial distribution, including the two‐tailed Fisher's exact test corrected using a Bonferroni correction. *STATISTICA* software, version 8.0 (StatSoft, Tulsa, OK, USA), was used for calculations.

## Results

### Frequency of phylogenetic groups, bacteriocinogeny, and bacteriocinogenic determinants in *E. coli* isolates

Results of phylogenetic analysis of 1283 *E. coli* isolates are shown in Table 1 and S1. The most common was phylogroup B2 (38.3%) followed by phylogroup A (28.3%), D (26.3%), and group B1 (7.2%).

**Table 1:** Phylogenetic groups, bacteriocinogeny, and detected bacteriocinogenic determinants identified among *Escherichia coli* isolates of human fecal origin

No. of strains/producers/determinants	*E. coli* phylogroup (%)				Statistical significance^a^
	A	B1	B2	D	
*E. coli* strains	363 (28.3)	92 (7.2)	491 (38.3)	337 (26.3)	
Bacteriocinogenic isolates^b^	186 (51.2)	41 (44.6)	296 (60.3)	172 (51.0)	B1 × B2: *P *=* *0.03
Colicin producers	75 (40.3)	16 (39.0)	51 (17.2)	58 (33.7)	A × B2: *P* __ 0.01; B1 × B2: *P *=* *0.01; B2 × D: *P* __ 0.01
Microcin producers	36 (19.4)	8 (19.5)	116 (39.2)	54 (31.4)	A × B2: *P* __0.01
Strains producing both colicins and microcins	75 (40.3)	17 (41.5)	129 (43.6)	58 (33.7)	–
Detected bacteriocin determinants	376	76	612	331	–
Detected colicin determinants	234 (62.2)	48 (63.2)	246 (40.2)	168 (50.8)	A × B2: *P* __ 0.01; A × D: *P *=* *0.02; B1 × B2: *P* __ 0.01; B2 × D: *P* __0.01
Detected microcin determinants	142 (37.8)	28 (36.8)	366 (59.8)	161 (48.6)	A × B2: *P* __ 0.01; A × D: *P *=* *0.02; B1 × B2: *P* __ 0.01; B2 × D: *P* __ 0.01
Unknown bacteriocin determinants^c^	–	–	–	2 (0.6)	–

a Statistically significant results (*P *≤* *0.05) are shown. Bonferroni correction was applied to correct for multiple tests.

b Colicin producers, microcin producers and strains producing both colicins and microcins among bacteriocinogenic *E. coli* isolates

c In two *E. coli* isolates, showing a clear zone of inhibition against indicator strains in the overlay test, no PCR products with bacteriocin‐specific primers were obtained

Altogether, 695 bacteriocin producers were identified representing 54.2% of all 1283 tested isolates. The number of detected bacteriocin producers and the number of detected bacteriocin determinants differed significantly between phylogroups (Table 1). The highest bacteriocinogeny was found in group B2 (60.3%; *P *=* *0.03 compared to group B1) and the lowest bacteriocinogeny was found in group B1 (44.6%).

Bacteriocin producers encoding only colicins (one or more) were significantly less common in phylogenetic group B2, compared to phylogroups A, B1, and D (*P *≤* *0.01), whereas producers encoding only microcins (one or more) were significantly more common in phylogroup B2, compared to phylogroup A (*P *<* *0.01) (Table 1).

While phylogroups A and B1 encoded colicin determinants most often (62.2% and 63.2%, respectively; *P *≤* *0.02 compared to groups B2 and D), colicin determinants were least common in phylogroup B2 (40.2%; *P* __ 0.01 compared to group A and B1). In contrast, phylogroup B2 encoded the highest number of microcin determinants (59.8%; *P* __ 0.01 compared to group A and B1).

No statistically significant differences were found in the occurrence of phylogenetic groups with respect to the patient gender or diagnosis and in the prevalence of bacteriocinogeny or bacteriocin determinants with respect to the patient diagnosis (data not shown). Altogether, 281 different genotypes were found among 1283 tested isolates when phylogroups and bacteriocin determinants were combined (data not shown).

### Frequency of individual bacteriocin‐encoding determinants

Genetic determinants encoding 30 bacteriocin types including 23 colicins (A, B, D, E1, E2‐9, Ia, Ib, Js, K, L, M, N, S4, U, Y, and 5/10) and seven microcins (mH47, mM, mB17, mC7, mJ25, mL, and mV) were tested in *E. coli* bacteriocin producers used in this study. Out of 1393 identified individual bacteriocin determinants, 696 and 697 encoded colicins and microcins, respectively. The most frequent bacteriocin determinants were those that encoded mH47 (*n *=* *224), Ia (*n *=* *211), mV (*n *=* *206), mM (*n *=* *163), E1 (*n *=* *139), and M (*n *=* *107). On the other hand, colicin E4, E9, and L, were not detected in any of the tested isolates. Results of detection of individual bacteriocin determinants are shown in Table 2 and S1. In phylogroup A, determinants encoding E1, Ia, and mV were most common, in phylogroup B2, microcins mM and mH47 were most common, and in phylogroup D, microcin B17 was most common.

**Table 2:** Detection of individual bacteriocin determinants in phylogenetic groups of bacteriocinogenic *Escherichia coli* of human fecal origin

	*E. coli* phylogroup				Statistical significance
	A (*n *=* *363)	B1 (*n *=* *92)	B2 (*n *=* *491)	D (*n *=* *337)	
Bacteriocin nonproducers	177 (48.8)	51 (55.4)	195 (39.7)	165 (49.0)	B1 × B2: *P *=* *0.03
Bacteriocin producers	186 (51.2)	41 (44.6)	296 (60.3)	172 (51.0)	B1 × B2: *P *=* *0.03

	A (%) (*n *=* *186)	B1 (%) (*n *=* *41)	B2 (%) (*n *=* *296)	D (%) (*n *=* *172)	Statistical significance
*Bacteriocin determinant*					
mV	68 (36.6%)	12 (29.3%)	86 (29.1%)	40 (23.3%)	A × D: *P *=* *0.04
mM	19 (10.2%)	5 (12.2%)	102 (34.5%)	37 (21.5%)	A × B2: *P *<* *0.01; A × D = 0.02; B1 × B2: *P *<* *0.01; B2 × D: *P *=* *0.02
mH47	37 (19.9%)	6 (14.6%)	137 (46.3%)	44 (25.6%)	A×B2: *P *<* *0.01; B1 × B2: *P *<* *0.01; B2 × D: *P *<* *0.01;
mB17	15 (8.1%)	4 (9.8%)	33 (11.1%)	33 (19.2%)	A × D: *P *=* *0.02
mC7	3 (1.6%)	1 (2.4%)	4 (1.4%)	4 (2.3%)	–
mJ25	–	–	1 (0.3%)	1 (0.6%)	–
mL	–	–	3 (1.0%)	2 (1.2%)	–
Ia	72 (38.7%)	14 (34.1%)	80 (27.0%)	45 (26.2%)	A × B2: *P *=* *0.04
Ib	24 (12.9%)	6 (14.6%)	23 (7.8%)	9 (5.2%)	
A	1 (0.5%)	–	1 (0.3%)	–	–
B	15 (8.1%)	6 (14.6%)	16 (5.4%)	11 (6.4%)	–
D	–	–	1 (0.3%)	–	–
E1	50 (26.9)	8 (19.5%)	46 (15.5%)	35 (20.3%)	A × B2: *P *=* *0.02
E2	–	–	2 (0.7%)	2 (1.2%)	–
E3	–	–	–	1 (0.6%)	–
E4	–	–	–	–	–
E5	–	–	1 (0.3%)	–	–
E6	2 (1.1%)	–	1 (0.3%)	–	–
E7	8 (4.3%)	3 (7.3%)	3 (1.0%)	2 (1.2%)	–
E8	1 (0.5%)	–	–	4 (2.3%)	–
E9	–	–	–	–	–
K	8 (4.3%)	1 (2.4%)	10 (3.4%)	8 (4.7%)	–
L	–	–	–	–	–
M	38 (20.4%)	9 (22.0%)	36 (12.2%)	24 (14.0%)	–
N	2 (1.1%)	–	8 (2.7%)	3 (1.7%)	–
S4	4 (2.2%)	1 (2.4%)	2 (0.7%)	6 (3.5%)	–
U	1 (0.5%)	–	–	2 (1.2%)	–
Y	1 (0.5%)	–	1 (0.3%)	1 (0.6%)	–
5/10	2 (1.1%)	–	4 (1.4%)	1 (0.6%)	–
Js	5 (2.7%)	–	11 (3.7%)	14 (8.1%)	–
Unknown	–	–	–	2 (1.2%)	–

*Statistically significant results (*P *≤* *0.05) are shown. Bonferroni correction was applied to correct for multiple tests.

## Discussion

Altogether, more than 54% of all tested isolates were identified as bacteriocin producers in this study. Our previous study (Šmajs et al. 2010) revealed bacteriocin production in 55% (226 out of 411) of fecal *E. coli* having similar human origins. Other studies on human fecal *E. coli* strains have revealed somewhat lower prevalences of bacteriocinogeny. A study by Gordon and O'Brien (2006) revealed, in a similar set of human *E. coli* strains, 102 bacteriocin‐producing strains among 266 tested (38%). However, this study detected only 18 bacteriocin determinants compared to 30 determinants used in our study (Gordon and O'Brien 2006). In our study, 76 bacteriocin determinants were found for those bacteriocin types not tested in the study of Gordon and O'Brien, theoretically representing 5.9% of bacteriocin producers. A study performed by Šmarda and Obdržálek (2001) detected bacteriocinogeny only on the plates with indicator strains and identified bacteriocin production in 41.37% of 1043 *E. coli* human fecal strains collected from 1993 to 1999. In contrast to previous studies, Kohoutová et al. (2014) found a relatively high frequency of bacteriocinogeny (65.0%) in fecal *E. coli* strains isolated from patient with colorectal adenoma and in strains isolated from patients with colorectal carcinoma (69.0%). In general, it appears that about one half of human *E. coli* strains of fecal origin synthesize one or more bacteriocins. The number of bacteriocin determinants for which there were no positive *E. coli* strains in each phylogroup (shown in Table 2) appeared to correlate with the number of investigated *E. coli* strains in each phylogroup. This suggests that each of the tested bacteriocin types can be encoded by any phylogroup and that there are no specific bacteriocin types exclusively associated with particular *E. coli* phylogroups.

The prevalence of bacteriocin producers and bacteriocin determinants among several *E. coli* isolates from the same person was analyzed by Kohoutová et al. (2014). This work characterized 622 isolates taken from mucosal biopsies from cecum, transverse colon, and rectum from patients with colorectal adenoma or carcinoma, and from healthy controls. On average, seven *E. coli* isolates were taken from each patient and slightly over two different *E. coli* strains were identified in each patient with respect to bacteriocin production. Other studies revealed similar results indicating that the prevalence of different *E. coli* strain per individual range from 1.4 to 2.5 (Moreno et al. 2006; Nielsen et al. 2014). These data indicate that despite observed variability among *E. coli* strains within a single patient, each niche is inhabited by one or few predominant *E. coli* strains. This study, in most cases, characterized predominant *E. coli* strains.

In this study, we demonstrated an increased bacteriocinogeny in *E. coli* phylogroup B2 (60.3%). A study of Budicˇ et al. (2011) detected an even greater increase in bacteriocinogeny in *E. coli* strains belonging to phylogroup B2 (89%). However, while our study was performed on human fecal *E. coli* strains, the study of Budicˇ et al. (2011) tested uropathogenic strains that were isolated from septic patients (*n *=* *105). Since most uropathogenic strains originate from fecal strains (Brumfitt and Hamilton‐Miller 1998), more virulent and therefore more frequently bacteriocinogenic strains could have been over‐represented in urinary tract and subsequent septic infections.

**Figure 1:**
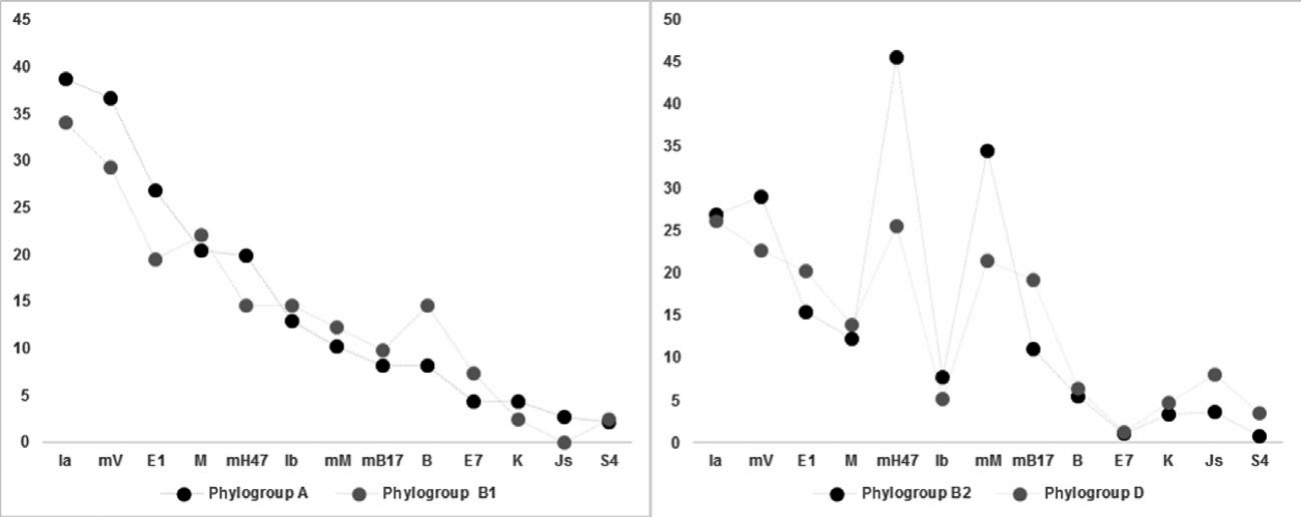
A graphical representation of the most prevalent bacteriocin determinants identified in different *Escherichia coli* phylogroups. The percentage of detected bacteriocin determinants out of all detected determinants in a particular phylogroup is shown for 13 of the most frequent bacteriocin determinants. Phylogroups A and B1 showed similar patterns of encoded bacteriocin determinants and similar bacteriocin genes were also prevalent in phylogroups B2 and D.

Compared to phylogroups B2 and D, phylogroup A and B1 tended to encoded more colicin types. In contrast, group B2 and D tended to encoded more microcins. The only exception was mV, an 8.7 kDa type IIa microcin (Duquesne et al. 2007) that was less common in phylogroup D. In our previous study, we found that prevalence of colicinogenic strains was higher in phylogroups A and D compared to phylogroup B2, while group B2 had a higher prevalence of microcin‐encoding strains compared to strains of group A or D (Šmajs et al. 2010). However, both human fecal and uropathogenic strains were tested in this study. Among uropathogenic strains, the majority of microcin‐encoding strains (72%) were in group B2 (Budicˇ et al. 2011). In this study, phylogroups A and B1 appear to be similar with respect to encoded bacteriocins and similar bacteriocins were also prevalent in phylogroups B2 and D (Fig. 1). The observed similarities in prevalence of bacteriocinogenic determinants within *E. coli* phylogroups are consistent with the phylogeny of *E. coli* strains where the B2 phylogroup is an ancestral group and group D is a sister group of the A+B1 clade, in which A and B1 are sister groups (Lecointre et al. 1998). Based on the accumulated sequencing data from multiple loci, additional phylogroups including group E (Tenaillon et al. 2010), group F (Jaureguy et al. 2008), group C (Moissenet et al. 2010), and *Escherichia* clade I (Luo et al. 2011) have been recently described. We have analyzed a set of randomly selected 96 *E. coli* isolates with a new Clermont et al. (2013) phylotyping method and isolates belonging to phylogroups C, E, and F represented 13.5% of the 96 analyzed *E. coli* isolates. The percentage of isolates belonging to phylogroups C, E, and F was similar to the data from the study of Clermont et al. (2013). Among 96 analyzed *E. coli* isolates, no statistically significant changes were observed in bacteriocinogeny and in distribution of bacteriocin determinants in the original as well as in the new phylogroups (data not shown). These observations suggest that additional typing to newly described phylogroups would result in no or in minor alteration of the results with respect to bacteriocinogeny and to distribution of bacteriocin determinants in *E. coli* phylogroups.

Both microcins mM and mH47 (which are very common in group B2 compared to other phylogroups) often co‐occur (Gordon and O'Brien 2006) and are chromosome‐encoded (Laviña et al. 1990; Welch et al. 2002). However, chromosomally encoded microcin determinants mM and mH47 can be detected in all *E. coli* phylogroups suggesting frequent horizontal gene transfer of the corresponding determinants. In addition to microcin mB17, phylogroup D also often encodes colicin Js, although in the case of colicin Js, it did not reach statistical significance (*P *=* *0.2). Interestingly, features of colicin Js overlap with both colicins and microcins (Šmajs and Weinstock 2001a,b).

A comparison with characterized bacteriocin determinants in Australian *E. coli* strains revealed that mB17 and mV bacteriocin types are less common among Australian strains while other bacteriocin determinants including E7 are more common in Australian compared to Czech strains (Gordon and O'Brien 2006); this suggests that the prevalence of bacteriocin genes differ in geographic locations. This finding is in agreement with previous sequence analysis of colicin‐encoding genes isolated from two geographically distinct groups of *E. coli* strains (isolated in the Czech Republic and Amazonia, Brazil), which revealed that colicin genes were very similar among the strains of each group but different between them (Šmajs et al. 2012), indicating that bacteriocin types and their gene sequences are population‐specific while *E. coli* strains are different in different geographic locations.

In humans, transient strains of *E. coli* more often belong to phylogroup B1, while resident *E. coli* strains more often belong to phylogroup B2 (Nowrouzian et al. 2005). Resident strains are detectable in the human intestines for months while transient strains persist for days to weeks (Sears et al. 1950, 1956; Sears and Brownlee 1952). The highest prevalence of bacteriocinogenic strains was found in the phylogroup B2 suggesting that bacteriocinogeny and especially the synthesis of microcins were associated with resident strains. Moreover, a previously published study revealed a direct correlation between prevalence of bacteriocinogeny and the number of different virulence factors encoded by a strain of *E. coli* (Micenková et al. 2014). Since virulence genes likely evolved and are being maintained to improve interhost persistence of commensal bacteria (Levin 1996; Le Gall et al. 2007) and since several virulence (i.e., with *aer*,* cnf1*,* fyuA*,* hlyA*,* iroCDN*,* iucC*,* papCG*,* sfa*,* tcpC*, and *usp*) and bacteriocin determinants (e.g., E1, Ia, S4 mB17, mE492, mH47, mI47, mM, and mV) are associated in *E. coli* strains (Azpiroz et al. 2009; Šmajs et al. 2010; Budicˇ et al. 2011; Petkovšek et al. 2012; Micenková et al. 2014), bacteriocin synthesis appears to further promote stable colonization of the gut. It is therefore likely that the selection for residency is the driving force for increased virulence and for increased frequency of bacteriocinogeny and predominant synthesis of microcins.

## Conflict of Interest

The authors declare that they have no competing interests.

## Supporting information


**Table S1.** All detected characteristics of *E. coli* isolates analyzed in this study.

**Table S2.** DNA Primers used for PCR detection of colicin and microcin‐encoding genes and genes encoding virulence factors.
