# Supplementary material for: Microcin determinants are associated with B2 phylogroup of human fecal Escherichia coli isolates
Source: Microbiologyopen. 2016 Mar 14;5(3):490–8. doi: 10.1002/mbo3.345 (PMC4906000; doi:10.1002/mbo3.345)
Supplement: Supplementary file 1 — Table S1. All detected characteristics of E. coli isolates analyzed in this study. [file MBO3-5-490-s001.docx]

| **Strain** | **Source** | **Sex** | **Diagnoses** | **Hospital** | **Year of isolation** | **Phylogenetic group** | **Bacteriocins** |
| --- | --- | --- | --- | --- | --- | --- | --- |
| A36 | Fecal E. coli | M | Diseases of the digestive system | St. Anne's University Hospital Brno | 2007 | D | Ia |
| A62 | Fecal E. coli | M | Diseases of the digestive system | St. Anne's University Hospital Brno | 2007 | B2 | N, mB17, mV |
| A110 | Fecal E. coli | F | Factors influencing health status and contact with health services | St. Anne's University Hospital Brno | 2007 | B2 | mH47, mV |
| A46 | Fecal E. coli | F | Neoplasms | St. Anne's University Hospital Brno | 2007 | A | mH47, mM, mV |
| A1 | Fecal E. coli | M | Diseases of the nervous system | St. Anne's University Hospital Brno | 2007 | B2 | mH47, mM |
| A107 | Fecal E. coli | F | Symptoms, signs and abnormal clinical and laboratory findings, not elsewhere classified | St. Anne's University Hospital Brno | 2007 | B2 | mH47, mM |
| A12 | Fecal E. coli | F | Factors influencing health status and contact with health services | St. Anne's University Hospital Brno | 2007 | D | mH47, mM |
| A144 | Fecal E. coli | F | Injury, poisoning and certain other consequences of external causes | St. Anne's University Hospital Brno | 2007 | B2 | mH47, mM |
| A180 | Fecal E. coli | F | Symptoms, signs and abnormal clinical and laboratory findings, not elsewhere classified | St. Anne's University Hospital Brno | 2007 | D | mH47, mM |
| A19 | Fecal E. coli | M | Neoplasms | St. Anne's University Hospital Brno | 2007 | D | mH47, mM |
| A21 | Fecal E. coli | F | Certain infectious and parasitic diseases | St. Anne's University Hospital Brno | 2007 | D | mH47, mM |
| A25 | Fecal E. coli | F | Diseases of the respiratory system | St. Anne's University Hospital Brno | 2007 | A | mH47, mM |
| A30 | Fecal E. coli | M | Diseases of the digestive system | St. Anne's University Hospital Brno | 2007 | D | mH47, mM |
| A42 | Fecal E. coli | F | Neoplasms | St. Anne's University Hospital Brno | 2007 | B2 | mH47, mM |
| A57 | Fecal E. coli | M | Symptoms, signs and abnormal clinical and laboratory findings, not elsewhere classified | St. Anne's University Hospital Brno | 2007 | B2 | mH47, mM |
| A7 | Fecal E. coli | M | Certain infectious and parasitic diseases | St. Anne's University Hospital Brno | 2007 | A | mH47, mM |
| A14 | Fecal E. coli | M | Diseases of the digestive system | St. Anne's University Hospital Brno | 2007 | D | mH47, mL, mM |
| A121 | Fecal E. coli | M | Neoplasms | St. Anne's University Hospital Brno | 2007 | B2 | mH47 |
| A122 | Fecal E. coli | F | Diseases of the digestive system | St. Anne's University Hospital Brno | 2007 | B2 | mH47 |
| A160 | Fecal E. coli | F | Diseases of the digestive system | St. Anne's University Hospital Brno | 2007 | B2 | mH47 |
| A39 | Fecal E. coli | F | Diseases of the circulatory system | St. Anne's University Hospital Brno | 2007 | A | mH47 |
| A53 | Fecal E. coli | F | Diseases of the digestive system | St. Anne's University Hospital Brno | 2007 | B2 | mH47 |
| A149 | Fecal E. coli | F | Diseases of the blood and blood-forming organs and certain disorders involving the immune mechanism | St. Anne's University Hospital Brno | 2007 | D | mC7 |
| A35 | Fecal E. coli | F | Neoplasms | St. Anne's University Hospital Brno | 2007 | B2 | mB17, mV |
| A33 | Fecal E. coli | M | Factors influencing health status and contact with health services | St. Anne's University Hospital Brno | 2007 | B2 | mB17, mC7, mH47, mM |
| A158 | Fecal E. coli | F | Diseases of the digestive system | St. Anne's University Hospital Brno | 2007 | D | mB17 |
| A32 | Fecal E. coli | F | Symptoms, signs and abnormal clinical and laboratory findings, not elsewhere classified | St. Anne's University Hospital Brno | 2007 | A | M |
| A64 | Fecal E. coli | F | Factors influencing health status and contact with health services | St. Anne's University Hospital Brno | 2007 | D | M |
| A139 | Fecal E. coli | F | Diseases of the circulatory system | St. Anne's University Hospital Brno | 2007 | B2 | K |
| A154 | Fecal E. coli | F | Diseases of the skin and subcutaneous tissue | St. Anne's University Hospital Brno | 2007 | D | K |
| A159 | Fecal E. coli | M | Factors influencing health status and contact with health services | St. Anne's University Hospital Brno | 2007 | B2 | Js, N |
| A48 | Fecal E. coli | M | Diseases of the genitourinary system | St. Anne's University Hospital Brno | 2007 | D | Js, mM |
| A49 | Fecal E. coli | F | Certain infectious and parasitic diseases | St. Anne's University Hospital Brno | 2007 | B2 | Js, mM |
| A179 | Fecal E. coli | M | Diseases of the digestive system | St. Anne's University Hospital Brno | 2007 | D | Js, mB17, mM |
| A63 | Fecal E. coli | M | Diseases of the digestive system | St. Anne's University Hospital Brno | 2007 | D | Js |
| A170 | Fecal E. coli | F | Diseases of the respiratory system | St. Anne's University Hospital Brno | 2007 | D | Ib, mV |
| A182 | Fecal E. coli | F | Diseases of the musculoskeletal system and connective tissue | St. Anne's University Hospital Brno | 2007 | B2 | Ib, mV |
| A45 | Fecal E. coli | F | Certain infectious and parasitic diseases | St. Anne's University Hospital Brno | 2007 | A | Ib, mV |
| A152 | Fecal E. coli | F | Diseases of the digestive system | St. Anne's University Hospital Brno | 2007 | D | Ib, mM, mV |
| A132 | Fecal E. coli | F | Factors influencing health status and contact with health services | St. Anne's University Hospital Brno | 2007 | A | Ib, mM |
| A181 | Fecal E. coli | F | Diseases of the circulatory system | St. Anne's University Hospital Brno | 2007 | B2 | Ib, mB17 |
| A5 | Fecal E. coli | F | Injury, poisoning and certain other consequences of external causes | St. Anne's University Hospital Brno | 2007 | A | Ib |
| A124 | Fecal E. coli | F | Factors influencing health status and contact with health services | St. Anne's University Hospital Brno | 2007 | A | Ia, mV |
| A148 | Fecal E. coli | F | Factors influencing health status and contact with health services | St. Anne's University Hospital Brno | 2007 | A | Ia, mV |
| A15 | Fecal E. coli | F | Neoplasms | St. Anne's University Hospital Brno | 2007 | B2 | Ia, mV |
| A2 | Fecal E. coli | M | Diseases of the blood and blood-forming organs and certain disorders involving the immune mechanism | St. Anne's University Hospital Brno | 2007 | B2 | Ia, mV |
| A4 | Fecal E. coli | F | Pregnancy, childbirth and the puerperium | St. Anne's University Hospital Brno | 2007 | B2 | Ia, mV |
| A6 | Fecal E. coli | F | Certain infectious and parasitic diseases | St. Anne's University Hospital Brno | 2007 | B2 | Ia, mV |
| A68 | Fecal E. coli | M | Symptoms, signs and abnormal clinical and laboratory findings, not elsewhere classified | St. Anne's University Hospital Brno | 2007 | A | Ia, mV |
| A104 | Fecal E. coli | F | Symptoms, signs and abnormal clinical and laboratory findings, not elsewhere classified | St. Anne's University Hospital Brno | 2007 | B2 | Ia, mM, mV |
| A102 | Fecal E. coli | F | Neoplasms | St. Anne's University Hospital Brno | 2007 | D | Ia, mH47, mM |
| A105 | Fecal E. coli | F | Diseases of the digestive system | St. Anne's University Hospital Brno | 2007 | D | Ia, mH47, mM |
| A129 | Fecal E. coli | M | Factors influencing health status and contact with health services | St. Anne's University Hospital Brno | 2007 | B2 | Ia, mH47, mM |
| A3 | Fecal E. coli | M | Neoplasms | St. Anne's University Hospital Brno | 2007 | B2 | Ia, mB17, mV |
| A24 | Fecal E. coli | F | Certain infectious and parasitic diseases | St. Anne's University Hospital Brno | 2007 | B2 | Ia, mB17, mH47, mM |
| A10 | Fecal E. coli | F | Injury, poisoning and certain other consequences of external causes | St. Anne's University Hospital Brno | 2007 | A | Ia, mB17 |
| A76 | Fecal E. coli | F | Diseases of the circulatory system | St. Anne's University Hospital Brno | 2007 | A | Ia, M, mV |
| A142 | Fecal E. coli | M | Diseases of the circulatory system | St. Anne's University Hospital Brno | 2007 | B2 | Ia, Js, mV |
| A153 | Fecal E. coli | M | Factors influencing health status and contact with health services | St. Anne's University Hospital Brno | 2007 | D | Ia |
| A156 | Fecal E. coli | F | Diseases of the skin and subcutaneous tissue | St. Anne's University Hospital Brno | 2007 | B2 | Ia |
| A164 | Fecal E. coli | M | Factors influencing health status and contact with health services | St. Anne's University Hospital Brno | 2007 | D | Ia |
| A71 | Fecal E. coli | M | Diseases of the circulatory system | St. Anne's University Hospital Brno | 2007 | B1 | E1, mB17 |
| A47 | Fecal E. coli | M | Factors influencing health status and contact with health services | St. Anne's University Hospital Brno | 2007 | D | E1, Js |
| A86 | Fecal E. coli | F | Diseases of the genitourinary system | St. Anne's University Hospital Brno | 2007 | A | E1, Ib, N, mV |
| A155 | Fecal E. coli | F | Diseases of the digestive system | St. Anne's University Hospital Brno | 2007 | B2 | E1, Ia, mV |
| A66 | Fecal E. coli | F | Neoplasms | St. Anne's University Hospital Brno | 2007 | A | E1, Ia, M, mV |
| A28 | Fecal E. coli | M | Diseases of the circulatory system | St. Anne's University Hospital Brno | 2007 | B2 | E1, Ia |
| A70 | Fecal E. coli | M | Neoplasms | St. Anne's University Hospital Brno | 2007 | D | E1, Ia |
| A127 | Fecal E. coli | F | Factors influencing health status and contact with health services | St. Anne's University Hospital Brno | 2007 | A | E1 |
| A145 | Fecal E. coli | F | Symptoms, signs and abnormal clinical and laboratory findings, not elsewhere classified | St. Anne's University Hospital Brno | 2007 | D | E1 |
| A26 | Fecal E. coli | F | Diseases of the digestive system | St. Anne's University Hospital Brno | 2007 | A | E1 |
| A55 | Fecal E. coli | F | Diseases of the digestive system | St. Anne's University Hospital Brno | 2007 | A | E1 |
| A146 | Fecal E. coli | F | Certain infectious and parasitic diseases | St. Anne's University Hospital Brno | 2007 | B2 | B, M, mV |
| A103 | Fecal E. coli | F | Factors influencing health status and contact with health services | St. Anne's University Hospital Brno | 2007 | B2 | B, M, mH47, mV |
| A40 | Fecal E. coli | M | Diseases of the digestive system | St. Anne's University Hospital Brno | 2007 | B2 | B, Ib, M, Y |
| A75 | Fecal E. coli | M | Certain infectious and parasitic diseases | St. Anne's University Hospital Brno | 2007 | A | B, Ia, M, mV |
| A38 | Fecal E. coli | M | Factors influencing health status and contact with health services | St. Anne's University Hospital Brno | 2007 | A | B, E7, M, mV |
| A27 | Fecal E. coli | F | Neoplasms | St. Anne's University Hospital Brno | 2007 | A | B, E1, M |
| A106 | Fecal E. coli | F | Diseases of the musculoskeletal system and connective tissue | St. Anne's University Hospital Brno | 2007 | B1 | - |
| A109 | Fecal E. coli | F | Diseases of the blood and blood-forming organs and certain disorders involving the immune mechanism | St. Anne's University Hospital Brno | 2007 | A | - |
| A11 | Fecal E. coli | M | Symptoms, signs and abnormal clinical and laboratory findings, not elsewhere classified | St. Anne's University Hospital Brno | 2007 | B2 | - |
| A113 | Fecal E. coli | M | Factors influencing health status and contact with health services | St. Anne's University Hospital Brno | 2007 | B2 | - |
| A114 | Fecal E. coli | F | Diseases of the circulatory system | St. Anne's University Hospital Brno | 2007 | A | - |
| A119 | Fecal E. coli | M | Diseases of the circulatory system | St. Anne's University Hospital Brno | 2007 | B2 | - |
| A120 | Fecal E. coli | M | Diseases of the digestive system | St. Anne's University Hospital Brno | 2007 | A | - |
| A123 | Fecal E. coli | F | Neoplasms | St. Anne's University Hospital Brno | 2007 | B2 | - |
| A125 | Fecal E. coli | F | Factors influencing health status and contact with health services | St. Anne's University Hospital Brno | 2007 | B2 | - |
| A126 | Fecal E. coli | M | Diseases of the skin and subcutaneous tissue | St. Anne's University Hospital Brno | 2007 | B2 | - |
| A13 | Fecal E. coli | M | Diseases of the digestive system | St. Anne's University Hospital Brno | 2007 | A | - |
| A141 | Fecal E. coli | M | Diseases of the digestive system | St. Anne's University Hospital Brno | 2007 | B2 | - |
| A143 | Fecal E. coli | M | Factors influencing health status and contact with health services | St. Anne's University Hospital Brno | 2007 | B2 | - |
| A147 | Fecal E. coli | F | Certain infectious and parasitic diseases | St. Anne's University Hospital Brno | 2007 | B2 | - |
| A150 | Fecal E. coli | M | Neoplasms | St. Anne's University Hospital Brno | 2007 | B2 | - |
| A151 | Fecal E. coli | M | Diseases of the digestive system | St. Anne's University Hospital Brno | 2007 | B1 | - |
| A157 | Fecal E. coli | M | Factors influencing health status and contact with health services | St. Anne's University Hospital Brno | 2007 | B2 | - |
| A16 | Fecal E. coli | M | Diseases of the genitourinary system | St. Anne's University Hospital Brno | 2007 | B2 | - |
| A161 | Fecal E. coli | F | Diseases of the digestive system | St. Anne's University Hospital Brno | 2007 | D | - |
| A168 | Fecal E. coli | M | Diseases of the digestive system | St. Anne's University Hospital Brno | 2007 | B2 | - |
| A169 | Fecal E. coli | M | Factors influencing health status and contact with health services | St. Anne's University Hospital Brno | 2007 | A | - |
| A17 | Fecal E. coli | M | Diseases of the digestive system | St. Anne's University Hospital Brno | 2007 | B2 | - |
| A171 | Fecal E. coli | F | Diseases of the nervous system | St. Anne's University Hospital Brno | 2007 | D | - |
| A176 | Fecal E. coli | F | Factors influencing health status and contact with health services | St. Anne's University Hospital Brno | 2007 | B2 | - |
| A177 | Fecal E. coli | F | Neoplasms | St. Anne's University Hospital Brno | 2007 | B2 | - |
| A178 | Fecal E. coli | F | Factors influencing health status and contact with health services | St. Anne's University Hospital Brno | 2007 | A | - |
| A18 | Fecal E. coli | F | Diseases of the skin and subcutaneous tissue | St. Anne's University Hospital Brno | 2007 | D | - |
| A183 | Fecal E. coli | F | Diseases of the digestive system | St. Anne's University Hospital Brno | 2007 | B2 | - |
| A20 | Fecal E. coli | M | Diseases of the digestive system | St. Anne's University Hospital Brno | 2007 | B2 | - |
| A22 | Fecal E. coli | F | Neoplasms | St. Anne's University Hospital Brno | 2007 | B2 | - |
| A23 | Fecal E. coli | M | Factors influencing health status and contact with health services | St. Anne's University Hospital Brno | 2007 | B2 | - |
| A29 | Fecal E. coli | F | Neoplasms | St. Anne's University Hospital Brno | 2007 | B2 | - |
| A31 | Fecal E. coli | F | Diseases of the circulatory system | St. Anne's University Hospital Brno | 2007 | B2 | - |
| A34 | Fecal E. coli | F | Diseases of the circulatory system | St. Anne's University Hospital Brno | 2007 | D | - |
| A37 | Fecal E. coli | F | Neoplasms | St. Anne's University Hospital Brno | 2007 | D | - |
| A41 | Fecal E. coli | F | Certain infectious and parasitic diseases | St. Anne's University Hospital Brno | 2007 | B2 | - |
| A43 | Fecal E. coli | M | Certain infectious and parasitic diseases | St. Anne's University Hospital Brno | 2007 | B2 | - |
| A44 | Fecal E. coli | M | Neoplasms | St. Anne's University Hospital Brno | 2007 | D | - |
| A50 | Fecal E. coli | F | Neoplasms | St. Anne's University Hospital Brno | 2007 | D | - |
| A51 | Fecal E. coli | M | Diseases of the digestive system | St. Anne's University Hospital Brno | 2007 | D | - |
| A52 | Fecal E. coli | F | Diseases of the digestive system | St. Anne's University Hospital Brno | 2007 | B2 | - |
| A54 | Fecal E. coli | M | Diseases of the circulatory system | St. Anne's University Hospital Brno | 2007 | A | - |
| A56 | Fecal E. coli | M | Diseases of the genitourinary system | St. Anne's University Hospital Brno | 2007 | A | - |
| A58 | Fecal E. coli | F | Diseases of the musculoskeletal system and connective tissue | St. Anne's University Hospital Brno | 2007 | D | - |
| A59 | Fecal E. coli | M | Diseases of the circulatory system | St. Anne's University Hospital Brno | 2007 | D | - |
| A60 | Fecal E. coli | F | Diseases of the nervous system | St. Anne's University Hospital Brno | 2007 | B2 | - |
| A61 | Fecal E. coli | F | Diseases of the digestive system | St. Anne's University Hospital Brno | 2007 | B2 | - |
| A65 | Fecal E. coli | F | Factors influencing health status and contact with health services | St. Anne's University Hospital Brno | 2007 | A | - |
| A67 | Fecal E. coli | F | Factors influencing health status and contact with health services | St. Anne's University Hospital Brno | 2007 | A | - |
| A69 | Fecal E. coli | F | Certain infectious and parasitic diseases | St. Anne's University Hospital Brno | 2007 | D | - |
| A8 | Fecal E. coli | F | Diseases of the circulatory system | St. Anne's University Hospital Brno | 2007 | A | - |
| A9 | Fecal E. coli | F | Diseases of the digestive system | St. Anne's University Hospital Brno | 2007 | B2 | - |
| B973 | Fecal E. coli | F | Diseases of the digestive system | University Hospital Brno | 2008 | D | unknown |
| B952 | Fecal E. coli | M | Certain infectious and parasitic diseases | University Hospital Brno | 2008 | B2 | S4, mH47, mM |
| B101 | Fecal E. coli | M | Certain infectious and parasitic diseases | University Hospital Brno | 2008 | D | S4, Js, mM |
| B232 | Fecal E. coli | F | Factors influencing health status and contact with health services | University Hospital Brno | 2008 | A | S4 |
| B38 | Fecal E. coli | M | Certain infectious and parasitic diseases | University Hospital Brno | 2008 | D | N, mH47, mM |
| B10 | Fecal E. coli | M | Diseases of the digestive system | University Hospital Brno | 2008 | D | mV |
| B161 | Fecal E. coli | F | Certain infectious and parasitic diseases | University Hospital Brno | 2008 | A | mV |
| B771 | Fecal E. coli | M | Certain infectious and parasitic diseases | University Hospital Brno | 2008 | A | mV |
| B78 | Fecal E. coli | F | Certain infectious and parasitic diseases | University Hospital Brno | 2008 | A | mV |
| B886 | Fecal E. coli | M | Certain infectious and parasitic diseases | University Hospital Brno | 2008 | D | mV |
| B887 | Fecal E. coli | M | Certain infectious and parasitic diseases | University Hospital Brno | 2008 | D | mV |
| B191 | Fecal E. coli | F | Symptoms, signs and abnormal clinical and laboratory findings, not elsewhere classified | University Hospital Brno | 2008 | D | mV |
| B64 | Fecal E. coli | M | Certain infectious and parasitic diseases | University Hospital Brno | 2008 | B2 | mM |
| B76 | Fecal E. coli | F | Symptoms, signs and abnormal clinical and laboratory findings, not elsewhere classified | University Hospital Brno | 2008 | B2 | mM |
| B366 | Fecal E. coli | F | Symptoms, signs and abnormal clinical and laboratory findings, not elsewhere classified | University Hospital Brno | 2008 | A | mH47, mV |
| B54 | Fecal E. coli | F | Symptoms, signs and abnormal clinical and laboratory findings, not elsewhere classified | University Hospital Brno | 2008 | A | mH47, mV |
| B945 | Fecal E. coli | F | Certain infectious and parasitic diseases | University Hospital Brno | 2008 | A | mH47, mV |
| A163 | Fecal E. coli | M | Diseases of the circulatory system | St. Anne's University Hospital Brno | 2008 | A | mH47, mM |
| A81 | Fecal E. coli | M | Symptoms, signs and abnormal clinical and laboratory findings, not elsewhere classified | St. Anne's University Hospital Brno | 2008 | D | mH47, mM |
| A82 | Fecal E. coli | M | Factors influencing health status and contact with health services | St. Anne's University Hospital Brno | 2008 | B2 | mH47, mM |
| A88 | Fecal E. coli | F | Diseases of the digestive system | St. Anne's University Hospital Brno | 2008 | D | mH47, mM |
| B118 | Fecal E. coli | M | Diseases of the digestive system | University Hospital Brno | 2008 | B2 | mH47, mM |
| B140 | Fecal E. coli | F | Symptoms, signs and abnormal clinical and laboratory findings, not elsewhere classified | University Hospital Brno | 2008 | B2 | mH47, mM |
| B189 | Fecal E. coli | M | Certain infectious and parasitic diseases | University Hospital Brno | 2008 | B2 | mH47, mM |
| B190 | Fecal E. coli | F | Certain infectious and parasitic diseases | University Hospital Brno | 2008 | B2 | mH47, mM |
| B46 | Fecal E. coli | M | Diseases of the digestive system | University Hospital Brno | 2008 | B2 | mH47, mM |
| B50 | Fecal E. coli | M | Diseases of the digestive system | University Hospital Brno | 2008 | B2 | mH47, mM |
| B71 | Fecal E. coli | F | Pregnancy, childbirth and the puerperium | University Hospital Brno | 2008 | D | mH47, mM |
| B81 | Fecal E. coli | M | Neoplasms | University Hospital Brno | 2008 | B2 | mH47, mM |
| B86 | Fecal E. coli | M | Diseases of the digestive system | University Hospital Brno | 2008 | B2 | mH47, mM |
| B997 | Fecal E. coli | F | Certain infectious and parasitic diseases | University Hospital Brno | 2008 | B2 | mH47, mM |
| B998 | Fecal E. coli | M | Certain infectious and parasitic diseases | University Hospital Brno | 2008 | A | mH47, mM |
| B155 | Fecal E. coli | M | Diseases of the digestive system | University Hospital Brno | 2008 | A | mH47, E7 |
| A115 | Fecal E. coli | M | Mental and behavioural disorders | St. Anne's University Hospital Brno | 2008 | A | mH47 |
| B148 | Fecal E. coli | M | Certain infectious and parasitic diseases | University Hospital Brno | 2008 | B1 | mH47 |
| B149 | Fecal E. coli | M | Certain infectious and parasitic diseases | University Hospital Brno | 2008 | B2 | mH47 |
| B169 | Fecal E. coli | F | Certain infectious and parasitic diseases | University Hospital Brno | 2008 | D | mH47 |
| B18 | Fecal E. coli | F | Certain infectious and parasitic diseases | University Hospital Brno | 2008 | A | mH47 |
| B183 | Fecal E. coli | M | Certain infectious and parasitic diseases | University Hospital Brno | 2008 | A | mH47 |
| B195 | Fecal E. coli | F | Certain infectious and parasitic diseases | University Hospital Brno | 2008 | D | mH47 |
| B200 | Fecal E. coli | F | Diseases of the digestive system | University Hospital Brno | 2008 | B2 | mH47 |
| B202 | Fecal E. coli | M | Certain infectious and parasitic diseases | University Hospital Brno | 2008 | B2 | mH47 |
| B203 | Fecal E. coli | F | Certain infectious and parasitic diseases | University Hospital Brno | 2008 | D | mH47 |
| B896 | Fecal E. coli | M | Certain infectious and parasitic diseases | University Hospital Brno | 2008 | D | mH47 |
| B897 | Fecal E. coli | F | Factors influencing health status and contact with health services | University Hospital Brno | 2008 | D | mH47 |
| B910 | Fecal E. coli | F | Certain infectious and parasitic diseases | University Hospital Brno | 2008 | B2 | mH47 |
| B911 | Fecal E. coli | F | Certain infectious and parasitic diseases | University Hospital Brno | 2008 | B2 | mH47 |
| B927 | Fecal E. coli | M | Diseases of the digestive system | University Hospital Brno | 2008 | B2 | mH47 |
| B939 | Fecal E. coli | M | Certain infectious and parasitic diseases | University Hospital Brno | 2008 | A | mH47 |
| B954 | Fecal E. coli | M | Certain infectious and parasitic diseases | University Hospital Brno | 2008 | D | mH47 |
| A77 | Fecal E. coli | M | Neoplasms | St. Anne's University Hospital Brno | 2008 | B2 | mC7, mM |
| B968 | Fecal E. coli | F | Certain infectious and parasitic diseases | University Hospital Brno | 2008 | B2 | mC7 |
| B67 | Fecal E. coli | M | Symptoms, signs and abnormal clinical and laboratory findings, not elsewhere classified | University Hospital Brno | 2008 | B2 | mB17, mM |
| B967 | Fecal E. coli | F | Certain infectious and parasitic diseases | University Hospital Brno | 2008 | B2 | mB17, mM |
| B873 | Fecal E. coli | M | Neoplasms | University Hospital Brno | 2008 | B2 | mB17, mH47, mM |
| B989 | Fecal E. coli | F | Diseases of the digestive system | University Hospital Brno | 2008 | A | mB17, mH47, mM |
| A78 | Fecal E. coli | F | Certain infectious and parasitic diseases | St. Anne's University Hospital Brno | 2008 | B2 | mB17 |
| B143 | Fecal E. coli | M | Certain infectious and parasitic diseases | University Hospital Brno | 2008 | B1 | mB17 |
| B17 | Fecal E. coli | F | Certain infectious and parasitic diseases | University Hospital Brno | 2008 | D | mB17 |
| B178 | Fecal E. coli | M | Certain infectious and parasitic diseases | University Hospital Brno | 2008 | D | mB17 |
| B24 | Fecal E. coli | M | Diseases of the respiratory system | University Hospital Brno | 2008 | D | mB17 |
| B293 | Fecal E. coli | F | Diseases of the nervous system | University Hospital Brno | 2008 | B2 | mB17 |
| B4 | Fecal E. coli | M | Factors influencing health status and contact with health services | University Hospital Brno | 2008 | B1 | mB17 |
| B978 | Fecal E. coli | F | Diseases of the skin and subcutaneous tissue | University Hospital Brno | 2008 | A | mB17 |
| B994 | Fecal E. coli | F | Diseases of the genitourinary system | University Hospital Brno | 2008 | A | mB17 |
| B139 | Fecal E. coli | M | Certain infectious and parasitic diseases | University Hospital Brno | 2008 | D | M, E1 |
| B883 | Fecal E. coli | M | Symptoms, signs and abnormal clinical and laboratory findings, not elsewhere classified | University Hospital Brno | 2008 | A | M, E1 |
| B942 | Fecal E. coli | M | Factors influencing health status and contact with health services | University Hospital Brno | 2008 | A | M, E1 |
| B906 | Fecal E. coli | F | Certain infectious and parasitic diseases | University Hospital Brno | 2008 | B1 | M, B, mV |
| B932 | Fecal E. coli | M | Factors influencing health status and contact with health services | University Hospital Brno | 2008 | D | M, B, mV |
| B953 | Fecal E. coli | F | Certain infectious and parasitic diseases | University Hospital Brno | 2008 | B2 | M, B, mV |
| B99 | Fecal E. coli | M | Certain infectious and parasitic diseases | University Hospital Brno | 2008 | A | M, B, mM |
| B100 | Fecal E. coli | M | Factors influencing health status and contact with health services | University Hospital Brno | 2008 | A | M, B, mH47 |
| B888 | Fecal E. coli | F | Certain infectious and parasitic diseases | University Hospital Brno | 2008 | B2 | M, B, mH47 |
| B923 | Fecal E. coli | M | Certain infectious and parasitic diseases | University Hospital Brno | 2008 | B2 | M, B, mH47 |
| B948 | Fecal E. coli | M | Certain infectious and parasitic diseases | University Hospital Brno | 2008 | D | M |
| B62 | Fecal E. coli | M | Diseases of the digestive system | University Hospital Brno | 2008 | A | K, mM |
| B70 | Fecal E. coli | M | Diseases of the nervous system | University Hospital Brno | 2008 | D | K, mH47, mM |
| B983 | Fecal E. coli | F | Certain infectious and parasitic diseases | University Hospital Brno | 2008 | A | K, mH47, mM |
| B61 | Fecal E. coli | M | Certain infectious and parasitic diseases | University Hospital Brno | 2008 | D | K, E8 |
| B971 | Fecal E. coli | M | Certain infectious and parasitic diseases | University Hospital Brno | 2008 | B2 | K |
| B83 | Fecal E. coli | F | Diseases of the digestive system | University Hospital Brno | 2008 | B2 | Js, mV |
| A135 | Fecal E. coli | F | Diseases of the digestive system | St. Anne's University Hospital Brno | 2008 | D | Js |
| B21 | Fecal E. coli | F | Certain infectious and parasitic diseases | University Hospital Brno | 2008 | B2 | Js |
| B22 | Fecal E. coli | F | Certain infectious and parasitic diseases | University Hospital Brno | 2008 | B2 | Js |
| B79 | Fecal E. coli | M | Diseases of the digestive system | University Hospital Brno | 2008 | B2 | Js |
| B903 | Fecal E. coli | M | Certain infectious and parasitic diseases | University Hospital Brno | 2008 | D | Js |
| A97 | Fecal E. coli | M | Factors influencing health status and contact with health services | St. Anne's University Hospital Brno | 2008 | B2 | Ib, mV |
| B150 | Fecal E. coli | M | Certain infectious and parasitic diseases | University Hospital Brno | 2008 | A | Ib, mV |
| B47 | Fecal E. coli | M | Diseases of the blood and blood-forming organs and certain disorders involving the immune mechanism | University Hospital Brno | 2008 | A | Ib, mV |
| B275 | Fecal E. coli | F | Certain infectious and parasitic diseases | University Hospital Brno | 2008 | B2 | Ib, mH47, mV |
| B206 | Fecal E. coli | F | Certain infectious and parasitic diseases | University Hospital Brno | 2008 | A | Ib, M, mV |
| B128 | Fecal E. coli | F | Diseases of the respiratory system | University Hospital Brno | 2008 | A | Ib, E1, mH47, mM |
| A172 | Fecal E. coli | M | Neoplasms | St. Anne's University Hospital Brno | 2008 | B2 | Ib |
| B138 | Fecal E. coli | M | Symptoms, signs and abnormal clinical and laboratory findings, not elsewhere classified | University Hospital Brno | 2008 | B1 | Ib |
| B145 | Fecal E. coli | M | Symptoms, signs and abnormal clinical and laboratory findings, not elsewhere classified | University Hospital Brno | 2008 | B2 | Ib |
| B234 | Fecal E. coli | M | Certain infectious and parasitic diseases | University Hospital Brno | 2008 | A | Ib |
| B29 | Fecal E. coli | M | Certain infectious and parasitic diseases | University Hospital Brno | 2008 | A | Ib |
| B921 | Fecal E. coli | M | Certain infectious and parasitic diseases | University Hospital Brno | 2008 | B2 | Ib |
| B990 | Fecal E. coli | M | Diseases of the skin and subcutaneous tissue | University Hospital Brno | 2008 | B2 | Ib |
| B127 | Fecal E. coli | F | Symptoms, signs and abnormal clinical and laboratory findings, not elsewhere classified | University Hospital Brno | 2008 | D | Ia, Y |
| B970 | Fecal E. coli | M | Symptoms, signs and abnormal clinical and laboratory findings, not elsewhere classified | University Hospital Brno | 2008 | A | Ia, U, Y |
| B769 | Fecal E. coli | F | Pregnancy, childbirth and the puerperium | University Hospital Brno | 2008 | D | Ia, U |
| B102 | Fecal E. coli | F | Diseases of the digestive system | University Hospital Brno | 2008 | A | Ia, S4 |
| A140 | Fecal E. coli | F | Neoplasms | St. Anne's University Hospital Brno | 2008 | A | Ia, mV |
| A175 | Fecal E. coli | F | Symptoms, signs and abnormal clinical and laboratory findings, not elsewhere classified | St. Anne's University Hospital Brno | 2008 | D | Ia, mV |
| B108 | Fecal E. coli | M | Certain infectious and parasitic diseases | University Hospital Brno | 2008 | B2 | Ia, mV |
| B159 | Fecal E. coli | F | Certain infectious and parasitic diseases | University Hospital Brno | 2008 | A | Ia, mV |
| B174 | Fecal E. coli | M | Certain infectious and parasitic diseases | University Hospital Brno | 2008 | B1 | Ia, mV |
| B182 | Fecal E. coli | M | Diseases of the digestive system | University Hospital Brno | 2008 | A | Ia, mV |
| B197 | Fecal E. coli | F | Certain infectious and parasitic diseases | University Hospital Brno | 2008 | D | Ia, mV |
| B23 | Fecal E. coli | M | Certain infectious and parasitic diseases | University Hospital Brno | 2008 | A | Ia, mV |
| B294 | Fecal E. coli | M | Certain infectious and parasitic diseases | University Hospital Brno | 2008 | A | Ia, mV |
| B41 | Fecal E. coli | F | Diseases of the skin and subcutaneous tissue | University Hospital Brno | 2008 | D | Ia, mV |
| B53 | Fecal E. coli | M | Neoplasms | University Hospital Brno | 2008 | A | Ia, mV |
| B585 | Fecal E. coli | F | Symptoms, signs and abnormal clinical and laboratory findings, not elsewhere classified | University Hospital Brno | 2008 | B2 | Ia, mV |
| B68 | Fecal E. coli | M | Diseases of the digestive system | University Hospital Brno | 2008 | B2 | Ia, mV |
| B877 | Fecal E. coli | F | Neoplasms | University Hospital Brno | 2008 | B2 | Ia, mV |
| B891 | Fecal E. coli | M | Certain infectious and parasitic diseases | University Hospital Brno | 2008 | B1 | Ia, mV |
| B898 | Fecal E. coli | F | Certain infectious and parasitic diseases | University Hospital Brno | 2008 | B1 | Ia, mV |
| B959 | Fecal E. coli | F | Certain infectious and parasitic diseases | University Hospital Brno | 2008 | A | Ia, mV |
| B966 | Fecal E. coli | F | Certain infectious and parasitic diseases | University Hospital Brno | 2008 | D | Ia, mV |
| B974 | Fecal E. coli | M | Certain infectious and parasitic diseases | University Hospital Brno | 2008 | B2 | Ia, mV |
| B975 | Fecal E. coli | M | Certain infectious and parasitic diseases | University Hospital Brno | 2008 | A | Ia, mV |
| B984 | Fecal E. coli | F | Certain infectious and parasitic diseases | University Hospital Brno | 2008 | A | Ia, mV |
| B988 | Fecal E. coli | F | Mental and behavioural disorders | University Hospital Brno | 2008 | A | Ia, mV |
| B34 | Fecal E. coli | M | Symptoms, signs and abnormal clinical and laboratory findings, not elsewhere classified | University Hospital Brno | 2008 | A | Ia, mM, mV |
| B986 | Fecal E. coli | F | Diseases of the circulatory system | University Hospital Brno | 2008 | D | Ia, mH47, mM |
| B75 | Fecal E. coli | M | Diseases of the musculoskeletal system and connective tissue | University Hospital Brno | 2008 | D | Ia, mC7, mJ25, mV |
| B48 | Fecal E. coli | F | Diseases of the digestive system | University Hospital Brno | 2008 | B2 | Ia, mB17, mH47, mM, mV |
| B179 | Fecal E. coli | F | Certain infectious and parasitic diseases | University Hospital Brno | 2008 | A | Ia, mB17 |
| B955 | Fecal E. coli | M | Certain infectious and parasitic diseases | University Hospital Brno | 2008 | A | Ia, mB17 |
| B160 | Fecal E. coli | M | Certain infectious and parasitic diseases | University Hospital Brno | 2008 | A | Ia, M, mV |
| B198 | Fecal E. coli | M | Certain infectious and parasitic diseases | University Hospital Brno | 2008 | B1 | Ia, M, B, mH47 |
| B137 | Fecal E. coli | M | Certain infectious and parasitic diseases | University Hospital Brno | 2008 | A | Ia, M |
| B63 | Fecal E. coli | F | Certain infectious and parasitic diseases | University Hospital Brno | 2008 | B2 | Ia, K, mV |
| B97 | Fecal E. coli | F | Symptoms, signs and abnormal clinical and laboratory findings, not elsewhere classified | University Hospital Brno | 2008 | D | Ia, K |
| B938 | Fecal E. coli | M | Certain infectious and parasitic diseases | University Hospital Brno | 2008 | B2 | Ia, E1, N, mV |
| B924 | Fecal E. coli | M | Certain infectious and parasitic diseases | University Hospital Brno | 2008 | A | Ia, E1, N, mC7, mV |
| B171 | Fecal E. coli | M | Certain infectious and parasitic diseases | University Hospital Brno | 2008 | B2 | Ia, E1, mV |
| B3 | Fecal E. coli | M | Factors influencing health status and contact with health services | University Hospital Brno | 2008 | B2 | Ia, E1, mV |
| B992 | Fecal E. coli | F | Symptoms, signs and abnormal clinical and laboratory findings, not elsewhere classified | University Hospital Brno | 2008 | A | Ia, E1, mV |
| B185 | Fecal E. coli | M | Certain infectious and parasitic diseases | University Hospital Brno | 2008 | B2 | Ia, E1, mB17, mV |
| B6 | Fecal E. coli | M | Diseases of the digestive system | University Hospital Brno | 2008 | B2 | Ia, E1, K, mV |
| B142 | Fecal E. coli | M | Certain infectious and parasitic diseases | University Hospital Brno | 2008 | A | Ia, E1, Js, mV |
| B125 | Fecal E. coli | F | Diseases of the digestive system | University Hospital Brno | 2008 | A | Ia, E1 |
| B144 | Fecal E. coli | M | Certain infectious and parasitic diseases | University Hospital Brno | 2008 | A | Ia, E1 |
| B889 | Fecal E. coli | M | Certain infectious and parasitic diseases | University Hospital Brno | 2008 | D | Ia, E1 |
| B123 | Fecal E. coli | M | Diseases of the digestive system | University Hospital Brno | 2008 | B2 | Ia, 5/10, mV |
| A73 | Fecal E. coli | F | Factors influencing health status and contact with health services | St. Anne's University Hospital Brno | 2008 | A | Ia |
| B104 | Fecal E. coli | F | Diseases of the digestive system | University Hospital Brno | 2008 | A | Ia |
| B121 | Fecal E. coli | M | Diseases of the digestive system | University Hospital Brno | 2008 | B2 | Ia |
| B141 | Fecal E. coli | M | Certain infectious and parasitic diseases | University Hospital Brno | 2008 | B1 | Ia |
| B19 | Fecal E. coli | M | Diseases of the blood and blood-forming organs and certain disorders involving the immune mechanism | University Hospital Brno | 2008 | D | Ia |
| B30 | Fecal E. coli | F | Symptoms, signs and abnormal clinical and laboratory findings, not elsewhere classified | University Hospital Brno | 2008 | A | Ia |
| B42 | Fecal E. coli | M | Certain infectious and parasitic diseases | University Hospital Brno | 2008 | A | Ia |
| B56 | Fecal E. coli | F | Certain infectious and parasitic diseases | University Hospital Brno | 2008 | D | Ia |
| B770 | Fecal E. coli | M | Diseases of the genitourinary system | University Hospital Brno | 2008 | A | Ia |
| B879 | Fecal E. coli | F | Diseases of the digestive system | University Hospital Brno | 2008 | D | Ia |
| B913 | Fecal E. coli | M | Certain infectious and parasitic diseases | University Hospital Brno | 2008 | D | Ia |
| B929 | Fecal E. coli | M | Injury, poisoning and certain other consequences of external causes | University Hospital Brno | 2008 | B2 | Ia |
| B93 | Fecal E. coli | F | Certain infectious and parasitic diseases | University Hospital Brno | 2008 | A | Ia |
| B960 | Fecal E. coli | F | Certain infectious and parasitic diseases | University Hospital Brno | 2008 | B2 | Ia |
| B961 | Fecal E. coli | M | Certain infectious and parasitic diseases | University Hospital Brno | 2008 | A | Ia |
| B969 | Fecal E. coli | M | Certain infectious and parasitic diseases | University Hospital Brno | 2008 | D | Ia |
| B979 | Fecal E. coli | F | Certain infectious and parasitic diseases | University Hospital Brno | 2008 | B1 | Ia |
| B204 | Fecal E. coli | M | Certain infectious and parasitic diseases | University Hospital Brno | 2008 | D | E8, M, B, mH47, mV |
| B31 | Fecal E. coli | F | Factors influencing health status and contact with health services | University Hospital Brno | 2008 | A | E7, Ib |
| B151 | Fecal E. coli | M | Certain infectious and parasitic diseases | University Hospital Brno | 2008 | D | E2 |
| B107 | Fecal E. coli | M | Certain infectious and parasitic diseases | University Hospital Brno | 2008 | A | E1, S4, mC7 |
| B933 | Fecal E. coli | F | Certain infectious and parasitic diseases | University Hospital Brno | 2008 | B2 | E1, N, mV |
| B109 | Fecal E. coli | F | Certain infectious and parasitic diseases | University Hospital Brno | 2008 | B2 | E1, mV |
| B167 | Fecal E. coli | M | Certain infectious and parasitic diseases | University Hospital Brno | 2008 | A | E1, mV |
| B205 | Fecal E. coli | F | Certain infectious and parasitic diseases | University Hospital Brno | 2008 | B2 | E1, mH47, mV |
| B963 | Fecal E. coli | F | Certain infectious and parasitic diseases | University Hospital Brno | 2008 | B2 | E1, mH47, mM |
| B184 | Fecal E. coli | M | Certain infectious and parasitic diseases | University Hospital Brno | 2008 | B2 | E1, mB17 |
| B45 | Fecal E. coli | M | Certain infectious and parasitic diseases | University Hospital Brno | 2008 | B2 | E1, mB17 |
| B9 | Fecal E. coli | M | Neoplasms | University Hospital Brno | 2008 | D | E1, mB17 |
| B925 | Fecal E. coli | M | Symptoms, signs and abnormal clinical and laboratory findings, not elsewhere classified | University Hospital Brno | 2008 | B2 | E1, mB17 |
| B106 | Fecal E. coli | F | Certain infectious and parasitic diseases | University Hospital Brno | 2008 | A | E1, K, mB17 |
| B90 | Fecal E. coli | F | Diseases of the musculoskeletal system and connective tissue | University Hospital Brno | 2008 | A | E1, K |
| B647 | Fecal E. coli | M | Factors influencing health status and contact with health services | University Hospital Brno | 2008 | A | E1, Js, mB17 |
| B60 | Fecal E. coli | F | Factors influencing health status and contact with health services | University Hospital Brno | 2008 | A | E1, Js |
| B131 | Fecal E. coli | F | Certain infectious and parasitic diseases | University Hospital Brno | 2008 | B2 | E1, Ib |
| B49 | Fecal E. coli | M | Certain infectious and parasitic diseases | University Hospital Brno | 2008 | A | E1, Ib |
| B207 | Fecal E. coli | M | Certain infectious and parasitic diseases | University Hospital Brno | 2008 | D | E1, Ia, M, mV |
| B201 | Fecal E. coli | M | Certain infectious and parasitic diseases | University Hospital Brno | 2008 | D | E1, E8, mB17, mH47, mM |
| A111 | Fecal E. coli | F | Diseases of the musculoskeletal system and connective tissue | St. Anne's University Hospital Brno | 2008 | B2 | E1, E7, Ib, mV |
| B126 | Fecal E. coli | F | Diseases of the digestive system | University Hospital Brno | 2008 | A | E1 |
| B132 | Fecal E. coli | M | Symptoms, signs and abnormal clinical and laboratory findings, not elsewhere classified | University Hospital Brno | 2008 | B2 | E1 |
| B135 | Fecal E. coli | M | Certain infectious and parasitic diseases | University Hospital Brno | 2008 | D | E1 |
| B156 | Fecal E. coli | F | Factors influencing health status and contact with health services | University Hospital Brno | 2008 | B2 | E1 |
| B448 | Fecal E. coli | M | Symptoms, signs and abnormal clinical and laboratory findings, not elsewhere classified | University Hospital Brno | 2008 | D | E1 |
| B52 | Fecal E. coli | F | Diseases of the digestive system | University Hospital Brno | 2008 | A | E1 |
| B74 | Fecal E. coli | F | Certain infectious and parasitic diseases | University Hospital Brno | 2008 | A | E1 |
| B881 | Fecal E. coli | F | Diseases of the digestive system | University Hospital Brno | 2008 | A | E1 |
| A85 | Fecal E. coli | M | Factors influencing health status and contact with health services | St. Anne's University Hospital Brno | 2008 | B2 | B, M, N, mV |
| B328 | Fecal E. coli | F | Diseases of the digestive system | University Hospital Brno | 2008 | A | B, M |
| B39 | Fecal E. coli | M | Diseases of the respiratory system | University Hospital Brno | 2008 | D | B, M |
| B8 | Fecal E. coli | F | Neoplasms | University Hospital Brno | 2008 | B2 | B, M |
| B914 | Fecal E. coli | F | Certain infectious and parasitic diseases | University Hospital Brno | 2008 | D | B, M |
| B916 | Fecal E. coli | F | Certain infectious and parasitic diseases | University Hospital Brno | 2008 | B2 | B, M |
| B92 | Fecal E. coli | F | Neoplasms | University Hospital Brno | 2008 | B2 | B, M |
| B985 | Fecal E. coli | F | Certain infectious and parasitic diseases | University Hospital Brno | 2008 | D | B, Ib, M, U |
| A101 | Fecal E. coli | F | Symptoms, signs and abnormal clinical and laboratory findings, not elsewhere classified | St. Anne's University Hospital Brno | 2008 | B2 | B, Ia, M, N, mV |
| B168 | Fecal E. coli | M | Certain infectious and parasitic diseases | University Hospital Brno | 2008 | A | B, Ia, M |
| B172 | Fecal E. coli | F | Certain infectious and parasitic diseases | University Hospital Brno | 2008 | A | B, Ia, M |
| B956 | Fecal E. coli | F | Factors influencing health status and contact with health services | University Hospital Brno | 2008 | B1 | B, Ia, M |
| B931 | Fecal E. coli | F | Certain infectious and parasitic diseases | University Hospital Brno | 2008 | A | B, Ia, K, M, mH47, mV |
| B767 | Fecal E. coli | M | Certain infectious and parasitic diseases | University Hospital Brno | 2008 | A | B |
| B186 | Fecal E. coli | M | Certain infectious and parasitic diseases | University Hospital Brno | 2008 | B2 | 5/10, mV |
| A100 | Fecal E. coli | F | Factors influencing health status and contact with health services | St. Anne's University Hospital Brno | 2008 | D | - |
| A112 | Fecal E. coli | F | Factors influencing health status and contact with health services | St. Anne's University Hospital Brno | 2008 | A | - |
| A116 | Fecal E. coli | F | Mental and behavioural disorders | St. Anne's University Hospital Brno | 2008 | A | - |
| A117 | Fecal E. coli | F | Diseases of the digestive system | St. Anne's University Hospital Brno | 2008 | B2 | - |
| A118 | Fecal E. coli | F | Factors influencing health status and contact with health services | St. Anne's University Hospital Brno | 2008 | B2 | - |
| A128 | Fecal E. coli | F | Diseases of the circulatory system | St. Anne's University Hospital Brno | 2008 | B2 | - |
| A130 | Fecal E. coli | F | Factors influencing health status and contact with health services | St. Anne's University Hospital Brno | 2008 | B2 | - |
| A133 | Fecal E. coli | M | Diseases of the digestive system | St. Anne's University Hospital Brno | 2008 | A | - |
| A134 | Fecal E. coli | F | Injury, poisoning and certain other consequences of external causes | St. Anne's University Hospital Brno | 2008 | D | - |
| A136 | Fecal E. coli | F | Factors influencing health status and contact with health services | St. Anne's University Hospital Brno | 2008 | B2 | - |
| A137 | Fecal E. coli | F | Factors influencing health status and contact with health services | St. Anne's University Hospital Brno | 2008 | B1 | - |
| A138 | Fecal E. coli | F | Neoplasms | St. Anne's University Hospital Brno | 2008 | B2 | - |
| A162 | Fecal E. coli | M | Diseases of the digestive system | St. Anne's University Hospital Brno | 2008 | B2 | - |
| A165 | Fecal E. coli | F | Diseases of the digestive system | St. Anne's University Hospital Brno | 2008 | B2 | - |
| A166 | Fecal E. coli | F | Diseases of the circulatory system | St. Anne's University Hospital Brno | 2008 | B2 | - |
| A167 | Fecal E. coli | F | Factors influencing health status and contact with health services | St. Anne's University Hospital Brno | 2008 | B2 | - |
| A173 | Fecal E. coli | F | Symptoms, signs and abnormal clinical and laboratory findings, not elsewhere classified | St. Anne's University Hospital Brno | 2008 | B2 | - |
| A174 | Fecal E. coli | F | Diseases of the digestive system | St. Anne's University Hospital Brno | 2008 | B2 | - |
| A72 | Fecal E. coli | M | Diseases of the digestive system | St. Anne's University Hospital Brno | 2008 | D | - |
| A74 | Fecal E. coli | F | Symptoms, signs and abnormal clinical and laboratory findings, not elsewhere classified | St. Anne's University Hospital Brno | 2008 | D | - |
| A79 | Fecal E. coli | M | Diseases of the digestive system | St. Anne's University Hospital Brno | 2008 | D | - |
| A80 | Fecal E. coli | M | Diseases of the digestive system | St. Anne's University Hospital Brno | 2008 | B2 | - |
| A83 | Fecal E. coli | M | Diseases of the digestive system | St. Anne's University Hospital Brno | 2008 | D | - |
| A84 | Fecal E. coli | M | Diseases of the skin and subcutaneous tissue | St. Anne's University Hospital Brno | 2008 | B2 | - |
| A87 | Fecal E. coli | F | Factors influencing health status and contact with health services | St. Anne's University Hospital Brno | 2008 | D | - |
| A89 | Fecal E. coli | M | Diseases of the digestive system | St. Anne's University Hospital Brno | 2008 | A | - |
| A90 | Fecal E. coli | F | Neoplasms | St. Anne's University Hospital Brno | 2008 | D | - |
| A91 | Fecal E. coli | F | Diseases of the genitourinary system | St. Anne's University Hospital Brno | 2008 | A | - |
| A92 | Fecal E. coli | F | Neoplasms | St. Anne's University Hospital Brno | 2008 | D | - |
| A93 | Fecal E. coli | F | Neoplasms | St. Anne's University Hospital Brno | 2008 | B2 | - |
| A94 | Fecal E. coli | F | Diseases of the circulatory system | St. Anne's University Hospital Brno | 2008 | A | - |
| A95 | Fecal E. coli | M | Diseases of the digestive system | St. Anne's University Hospital Brno | 2008 | B2 | - |
| A96 | Fecal E. coli | M | Symptoms, signs and abnormal clinical and laboratory findings, not elsewhere classified | St. Anne's University Hospital Brno | 2008 | A | - |
| A98 | Fecal E. coli | M | Diseases of the digestive system | St. Anne's University Hospital Brno | 2008 | A | - |
| A99 | Fecal E. coli | F | Factors influencing health status and contact with health services | St. Anne's University Hospital Brno | 2008 | B2 | - |
| B1 | Fecal E. coli | M | Certain infectious and parasitic diseases | University Hospital Brno | 2008 | A | - |
| B103 | Fecal E. coli | F | Certain infectious and parasitic diseases | University Hospital Brno | 2008 | B1 | - |
| B105 | Fecal E. coli | M | Certain infectious and parasitic diseases | University Hospital Brno | 2008 | B1 | - |
| B11 | Fecal E. coli | M | Neoplasms | University Hospital Brno | 2008 | B2 | - |
| B110 | Fecal E. coli | F | Certain infectious and parasitic diseases | University Hospital Brno | 2008 | A | - |
| B119 | Fecal E. coli | M | Diseases of the digestive system | University Hospital Brno | 2008 | B1 | - |
| B12 | Fecal E. coli | M | Symptoms, signs and abnormal clinical and laboratory findings, not elsewhere classified | University Hospital Brno | 2008 | B2 | - |
| B120 | Fecal E. coli | F | Diseases of the digestive system | University Hospital Brno | 2008 | A | - |
| B122 | Fecal E. coli | F | Diseases of the digestive system | University Hospital Brno | 2008 | A | - |
| B124 | Fecal E. coli | F | Certain infectious and parasitic diseases | University Hospital Brno | 2008 | B1 | - |
| B13 | Fecal E. coli | M | Diseases of the digestive system | University Hospital Brno | 2008 | A | - |
| B133 | Fecal E. coli | F | Diseases of the genitourinary system | University Hospital Brno | 2008 | B2 | - |
| B134 | Fecal E. coli | M | Diseases of the digestive system | University Hospital Brno | 2008 | B1 | - |
| B136 | Fecal E. coli | F | Neoplasms | University Hospital Brno | 2008 | B1 | - |
| B14 | Fecal E. coli | F | Certain infectious and parasitic diseases | University Hospital Brno | 2008 | B2 | - |
| B146 | Fecal E. coli | M | Certain infectious and parasitic diseases | University Hospital Brno | 2008 | A | - |
| B147 | Fecal E. coli | M | Certain infectious and parasitic diseases | University Hospital Brno | 2008 | D | - |
| B15 | Fecal E. coli | F | Diseases of the digestive system | University Hospital Brno | 2008 | D | - |
| B152 | Fecal E. coli | F | Certain infectious and parasitic diseases | University Hospital Brno | 2008 | B1 | - |
| B153 | Fecal E. coli | F | Diseases of the digestive system | University Hospital Brno | 2008 | B1 | - |
| B154 | Fecal E. coli | M | Factors influencing health status and contact with health services | University Hospital Brno | 2008 | D | - |
| B157 | Fecal E. coli | M | Certain infectious and parasitic diseases | University Hospital Brno | 2008 | B1 | - |
| B158 | Fecal E. coli | M | Certain infectious and parasitic diseases | University Hospital Brno | 2008 | B2 | - |
| B16 | Fecal E. coli | M | Symptoms, signs and abnormal clinical and laboratory findings, not elsewhere classified | University Hospital Brno | 2008 | A | - |
| B162 | Fecal E. coli | M | Certain infectious and parasitic diseases | University Hospital Brno | 2008 | A | - |
| B163 | Fecal E. coli | M | Certain infectious and parasitic diseases | University Hospital Brno | 2008 | B2 | - |
| B164 | Fecal E. coli | F | Certain infectious and parasitic diseases | University Hospital Brno | 2008 | A | - |
| B165 | Fecal E. coli | F | Diseases of the digestive system | University Hospital Brno | 2008 | B2 | - |
| B166 | Fecal E. coli | M | Certain infectious and parasitic diseases | University Hospital Brno | 2008 | B2 | - |
| B170 | Fecal E. coli | M | Certain infectious and parasitic diseases | University Hospital Brno | 2008 | A | - |
| B173 | Fecal E. coli | F | Diseases of the musculoskeletal system and connective tissue | University Hospital Brno | 2008 | D | - |
| B175 | Fecal E. coli | F | Certain infectious and parasitic diseases | University Hospital Brno | 2008 | B2 | - |
| B176 | Fecal E. coli | M | Certain infectious and parasitic diseases | University Hospital Brno | 2008 | D | - |
| B177 | Fecal E. coli | F | Symptoms, signs and abnormal clinical and laboratory findings, not elsewhere classified | University Hospital Brno | 2008 | D | - |
| B180 | Fecal E. coli | M | Certain infectious and parasitic diseases | University Hospital Brno | 2008 | D | - |
| B181 | Fecal E. coli | M | Certain infectious and parasitic diseases | University Hospital Brno | 2008 | D | - |
| B187 | Fecal E. coli | M | Symptoms, signs and abnormal clinical and laboratory findings, not elsewhere classified | University Hospital Brno | 2008 | A | - |
| B188 | Fecal E. coli | M | Certain infectious and parasitic diseases | University Hospital Brno | 2008 | B2 | - |
| B192 | Fecal E. coli | M | Certain infectious and parasitic diseases | University Hospital Brno | 2008 | D | - |
| B193 | Fecal E. coli | M | Diseases of the circulatory system | University Hospital Brno | 2008 | D | - |
| B194 | Fecal E. coli | F | Certain infectious and parasitic diseases | University Hospital Brno | 2008 | D | - |
| B196 | Fecal E. coli | F | Certain infectious and parasitic diseases | University Hospital Brno | 2008 | D | - |
| B199 | Fecal E. coli | F | Factors influencing health status and contact with health services | University Hospital Brno | 2008 | D | - |
| B2 | Fecal E. coli | F | Symptoms, signs and abnormal clinical and laboratory findings, not elsewhere classified | University Hospital Brno | 2008 | B2 | - |
| B20 | Fecal E. coli | M | Certain infectious and parasitic diseases | University Hospital Brno | 2008 | B2 | - |
| B209 | Fecal E. coli | F | Diseases of the digestive system | University Hospital Brno | 2008 | B2 | - |
| B218 | Fecal E. coli | F | Certain infectious and parasitic diseases | University Hospital Brno | 2008 | A | - |
| B228 | Fecal E. coli | F | Certain infectious and parasitic diseases | University Hospital Brno | 2008 | A | - |
| B229 | Fecal E. coli | M | Diseases of the digestive system | University Hospital Brno | 2008 | A | - |
| B230 | Fecal E. coli | F | Symptoms, signs and abnormal clinical and laboratory findings, not elsewhere classified | University Hospital Brno | 2008 | B2 | - |
| B231 | Fecal E. coli | F | Certain infectious and parasitic diseases | University Hospital Brno | 2008 | D | - |
| B233 | Fecal E. coli | F | Certain infectious and parasitic diseases | University Hospital Brno | 2008 | D | - |
| B235 | Fecal E. coli | M | Certain infectious and parasitic diseases | University Hospital Brno | 2008 | D | - |
| B236 | Fecal E. coli | M | Certain infectious and parasitic diseases | University Hospital Brno | 2008 | A | - |
| B237 | Fecal E. coli | M | Certain infectious and parasitic diseases | University Hospital Brno | 2008 | A | - |
| B25 | Fecal E. coli | F | Diseases of the digestive system | University Hospital Brno | 2008 | B2 | - |
| B253 | Fecal E. coli | M | Factors influencing health status and contact with health services | University Hospital Brno | 2008 | B2 | - |
| B26 | Fecal E. coli | F | Diseases of the respiratory system | University Hospital Brno | 2008 | D | - |
| B260 | Fecal E. coli | F | Certain infectious and parasitic diseases | University Hospital Brno | 2008 | B1 | - |
| B27 | Fecal E. coli | F | Certain infectious and parasitic diseases | University Hospital Brno | 2008 | A | - |
| B28 | Fecal E. coli | F | Certain infectious and parasitic diseases | University Hospital Brno | 2008 | D | - |
| B282 | Fecal E. coli | M | Diseases of the respiratory system | University Hospital Brno | 2008 | B2 | - |
| B301 | Fecal E. coli | M | Diseases of the digestive system | University Hospital Brno | 2008 | D | - |
| B307 | Fecal E. coli | F | Neoplasms | University Hospital Brno | 2008 | A | - |
| B32 | Fecal E. coli | F | Diseases of the genitourinary system | University Hospital Brno | 2008 | B2 | - |
| B33 | Fecal E. coli | F | Diseases of the digestive system | University Hospital Brno | 2008 | D | - |
| B337 | Fecal E. coli | M | Symptoms, signs and abnormal clinical and laboratory findings, not elsewhere classified | University Hospital Brno | 2008 | B2 | - |
| B343 | Fecal E. coli | M | Diseases of the digestive system | University Hospital Brno | 2008 | B1 | - |
| B35 | Fecal E. coli | F | Certain infectious and parasitic diseases | University Hospital Brno | 2008 | A | - |
| B358 | Fecal E. coli | F | Certain infectious and parasitic diseases | University Hospital Brno | 2008 | B1 | - |
| B36 | Fecal E. coli | F | Diseases of the genitourinary system | University Hospital Brno | 2008 | D | - |
| B365 | Fecal E. coli | F | Certain infectious and parasitic diseases | University Hospital Brno | 2008 | A | - |
| B368 | Fecal E. coli | F | Certain infectious and parasitic diseases | University Hospital Brno | 2008 | B1 | - |
| B37 | Fecal E. coli | F | Certain infectious and parasitic diseases | University Hospital Brno | 2008 | D | - |
| B40 | Fecal E. coli | M | Diseases of the respiratory system | University Hospital Brno | 2008 | B2 | - |
| B43 | Fecal E. coli | M | Certain infectious and parasitic diseases | University Hospital Brno | 2008 | D | - |
| B44 | Fecal E. coli | F | Symptoms, signs and abnormal clinical and laboratory findings, not elsewhere classified | University Hospital Brno | 2008 | B2 | - |
| B5 | Fecal E. coli | M | Factors influencing health status and contact with health services | University Hospital Brno | 2008 | B1 | - |
| B51 | Fecal E. coli | F | Factors influencing health status and contact with health services | University Hospital Brno | 2008 | B1 | - |
| B533 | Fecal E. coli | F | Diseases of the digestive system | University Hospital Brno | 2008 | D | - |
| B55 | Fecal E. coli | F | Certain infectious and parasitic diseases | University Hospital Brno | 2008 | B2 | - |
| B57 | Fecal E. coli | F | Certain infectious and parasitic diseases | University Hospital Brno | 2008 | D | - |
| B58 | Fecal E. coli | M | Diseases of the digestive system | University Hospital Brno | 2008 | B2 | - |
| B59 | Fecal E. coli | F | Certain infectious and parasitic diseases | University Hospital Brno | 2008 | A | - |
| B65 | Fecal E. coli | M | Diseases of the digestive system | University Hospital Brno | 2008 | A | - |
| B66 | Fecal E. coli | M | Factors influencing health status and contact with health services | University Hospital Brno | 2008 | B2 | - |
| B69 | Fecal E. coli | F | Diseases of the digestive system | University Hospital Brno | 2008 | A | - |
| B7 | Fecal E. coli | M | Diseases of the digestive system | University Hospital Brno | 2008 | D | - |
| B704 | Fecal E. coli | M | Diseases of the digestive system | University Hospital Brno | 2008 | B1 | - |
| B72 | Fecal E. coli | F | Diseases of the digestive system | University Hospital Brno | 2008 | D | - |
| B73 | Fecal E. coli | M | Factors influencing health status and contact with health services | University Hospital Brno | 2008 | A | - |
| B768 | Fecal E. coli | F | Diseases of the digestive system | University Hospital Brno | 2008 | A | - |
| B77 | Fecal E. coli | F | Diseases of the musculoskeletal system and connective tissue | University Hospital Brno | 2008 | B1 | - |
| B792 | Fecal E. coli | M | Diseases of the respiratory system | University Hospital Brno | 2008 | B2 | - |
| B80 | Fecal E. coli | M | Diseases of the digestive system | University Hospital Brno | 2008 | A | - |
| B82 | Fecal E. coli | F | Certain infectious and parasitic diseases | University Hospital Brno | 2008 | B2 | - |
| B84 | Fecal E. coli | F | Diseases of the digestive system | University Hospital Brno | 2008 | D | - |
| B85 | Fecal E. coli | M | Diseases of the digestive system | University Hospital Brno | 2008 | B1 | - |
| B87 | Fecal E. coli | F | Certain infectious and parasitic diseases | University Hospital Brno | 2008 | A | - |
| B872 | Fecal E. coli | M | Certain infectious and parasitic diseases | University Hospital Brno | 2008 | B2 | - |
| B874 | Fecal E. coli | M | Certain infectious and parasitic diseases | University Hospital Brno | 2008 | A | - |
| B875 | Fecal E. coli | F | Diseases of the genitourinary system | University Hospital Brno | 2008 | B2 | - |
| B876 | Fecal E. coli | F | Factors influencing health status and contact with health services | University Hospital Brno | 2008 | B2 | - |
| B878 | Fecal E. coli | M | Certain infectious and parasitic diseases | University Hospital Brno | 2008 | B2 | - |
| B88 | Fecal E. coli | M | Certain infectious and parasitic diseases | University Hospital Brno | 2008 | A | - |
| B880 | Fecal E. coli | F | Diseases of the digestive system | University Hospital Brno | 2008 | B2 | - |
| B882 | Fecal E. coli | M | Diseases of the digestive system | University Hospital Brno | 2008 | B1 | - |
| B884 | Fecal E. coli | M | Certain infectious and parasitic diseases | University Hospital Brno | 2008 | B2 | - |
| B885 | Fecal E. coli | F | Diseases of the circulatory system | University Hospital Brno | 2008 | B2 | - |
| B89 | Fecal E. coli | M | Certain infectious and parasitic diseases | University Hospital Brno | 2008 | B2 | - |
| B890 | Fecal E. coli | M | Symptoms, signs and abnormal clinical and laboratory findings, not elsewhere classified | University Hospital Brno | 2008 | A | - |
| B892 | Fecal E. coli | M | Certain infectious and parasitic diseases | University Hospital Brno | 2008 | D | - |
| B893 | Fecal E. coli | F | Certain infectious and parasitic diseases | University Hospital Brno | 2008 | A | - |
| B894 | Fecal E. coli | F | Certain infectious and parasitic diseases | University Hospital Brno | 2008 | A | - |
| B895 | Fecal E. coli | M | Certain infectious and parasitic diseases | University Hospital Brno | 2008 | D | - |
| B899 | Fecal E. coli | F | Certain infectious and parasitic diseases | University Hospital Brno | 2008 | A | - |
| B900 | Fecal E. coli | F | Certain infectious and parasitic diseases | University Hospital Brno | 2008 | D | - |
| B901 | Fecal E. coli | M | Endocrine, nutritional and metabolic diseases | University Hospital Brno | 2008 | B1 | - |
| B902 | Fecal E. coli | F | Certain infectious and parasitic diseases | University Hospital Brno | 2008 | B2 | - |
| B904 | Fecal E. coli | M | Certain infectious and parasitic diseases | University Hospital Brno | 2008 | D | - |
| B905 | Fecal E. coli | M | Symptoms, signs and abnormal clinical and laboratory findings, not elsewhere classified | University Hospital Brno | 2008 | B2 | - |
| B907 | Fecal E. coli | F | Certain infectious and parasitic diseases | University Hospital Brno | 2008 | B1 | - |
| B908 | Fecal E. coli | F | Certain infectious and parasitic diseases | University Hospital Brno | 2008 | B1 | - |
| B909 | Fecal E. coli | M | Certain infectious and parasitic diseases | University Hospital Brno | 2008 | D | - |
| B91 | Fecal E. coli | M | Endocrine, nutritional and metabolic diseases | University Hospital Brno | 2008 | D | - |
| B912 | Fecal E. coli | M | Diseases of the digestive system | University Hospital Brno | 2008 | D | - |
| B915 | Fecal E. coli | F | Certain infectious and parasitic diseases | University Hospital Brno | 2008 | D | - |
| B917 | Fecal E. coli | M | Certain infectious and parasitic diseases | University Hospital Brno | 2008 | A | - |
| B918 | Fecal E. coli | F | Certain infectious and parasitic diseases | University Hospital Brno | 2008 | A | - |
| B919 | Fecal E. coli | F | Certain infectious and parasitic diseases | University Hospital Brno | 2008 | A | - |
| B920 | Fecal E. coli | M | Certain infectious and parasitic diseases | University Hospital Brno | 2008 | B2 | - |
| B922 | Fecal E. coli | M | Factors influencing health status and contact with health services | University Hospital Brno | 2008 | A | - |
| B926 | Fecal E. coli | M | Certain infectious and parasitic diseases | University Hospital Brno | 2008 | D | - |
| B928 | Fecal E. coli | M | Certain infectious and parasitic diseases | University Hospital Brno | 2008 | D | - |
| B930 | Fecal E. coli | M | Diseases of the digestive system | University Hospital Brno | 2008 | D | - |
| B934 | Fecal E. coli | F | Certain infectious and parasitic diseases | University Hospital Brno | 2008 | B1 | - |
| B935 | Fecal E. coli | F | Certain infectious and parasitic diseases | University Hospital Brno | 2008 | D | - |
| B936 | Fecal E. coli | M | Certain infectious and parasitic diseases | University Hospital Brno | 2008 | B2 | - |
| B937 | Fecal E. coli | F | Certain infectious and parasitic diseases | University Hospital Brno | 2008 | B2 | - |
| B94 | Fecal E. coli | M | Certain infectious and parasitic diseases | University Hospital Brno | 2008 | A | - |
| B940 | Fecal E. coli | M | Symptoms, signs and abnormal clinical and laboratory findings, not elsewhere classified | University Hospital Brno | 2008 | B1 | - |
| B941 | Fecal E. coli | M | Certain infectious and parasitic diseases | University Hospital Brno | 2008 | B1 | - |
| B943 | Fecal E. coli | M | Certain infectious and parasitic diseases | University Hospital Brno | 2008 | D | - |
| B944 | Fecal E. coli | M | Certain infectious and parasitic diseases | University Hospital Brno | 2008 | B1 | - |
| B946 | Fecal E. coli | M | Factors influencing health status and contact with health services | University Hospital Brno | 2008 | A | - |
| B947 | Fecal E. coli | F | Certain infectious and parasitic diseases | University Hospital Brno | 2008 | A | - |
| B949 | Fecal E. coli | M | Certain infectious and parasitic diseases | University Hospital Brno | 2008 | A | - |
| B95 | Fecal E. coli | F | Diseases of the digestive system | University Hospital Brno | 2008 | A | - |
| B950 | Fecal E. coli | F | Certain infectious and parasitic diseases | University Hospital Brno | 2008 | A | - |
| B951 | Fecal E. coli | F | Certain infectious and parasitic diseases | University Hospital Brno | 2008 | A | - |
| B957 | Fecal E. coli | M | Certain infectious and parasitic diseases | University Hospital Brno | 2008 | A | - |
| B958 | Fecal E. coli | M | Certain infectious and parasitic diseases | University Hospital Brno | 2008 | A | - |
| B96 | Fecal E. coli | F | Diseases of the skin and subcutaneous tissue | University Hospital Brno | 2008 | D | - |
| B962 | Fecal E. coli | F | Certain infectious and parasitic diseases | University Hospital Brno | 2008 | D | - |
| B964 | Fecal E. coli | M | Certain infectious and parasitic diseases | University Hospital Brno | 2008 | B2 | - |
| B965 | Fecal E. coli | F | Certain infectious and parasitic diseases | University Hospital Brno | 2008 | D | - |
| B972 | Fecal E. coli | F | Certain infectious and parasitic diseases | University Hospital Brno | 2008 | D | - |
| B976 | Fecal E. coli | F | Certain infectious and parasitic diseases | University Hospital Brno | 2008 | D | - |
| B977 | Fecal E. coli | F | Certain infectious and parasitic diseases | University Hospital Brno | 2008 | A | - |
| B98 | Fecal E. coli | M | Certain infectious and parasitic diseases | University Hospital Brno | 2008 | D | - |
| B980 | Fecal E. coli | F | Diseases of the skin and subcutaneous tissue | University Hospital Brno | 2008 | D | - |
| B981 | Fecal E. coli | M | Certain infectious and parasitic diseases | University Hospital Brno | 2008 | B1 | - |
| B982 | Fecal E. coli | F | Diseases of the respiratory system | University Hospital Brno | 2008 | B2 | - |
| B987 | Fecal E. coli | F | Diseases of the digestive system | University Hospital Brno | 2008 | A | - |
| B991 | Fecal E. coli | F | Diseases of the respiratory system | University Hospital Brno | 2008 | B2 | - |
| B993 | Fecal E. coli | F | Certain infectious and parasitic diseases | University Hospital Brno | 2008 | D | - |
| B995 | Fecal E. coli | F | Certain infectious and parasitic diseases | University Hospital Brno | 2008 | A | - |
| B996 | Fecal E. coli | M | Certain infectious and parasitic diseases | University Hospital Brno | 2008 | A | - |
| B423 | Fecal E. coli | F | Diseases of the digestive system | University Hospital Brno | 2009 | B2 | N, mV |
| B434 | Fecal E. coli | M | Endocrine, nutritional and metabolic diseases | University Hospital Brno | 2009 | B2 | N, mV |
| B224 | Fecal E. coli | F | Certain infectious and parasitic diseases | University Hospital Brno | 2009 | B2 | mV |
| B225 | Fecal E. coli | M | Factors influencing health status and contact with health services | University Hospital Brno | 2009 | A | mV |
| B376 | Fecal E. coli | M | Certain infectious and parasitic diseases | University Hospital Brno | 2009 | A | mV |
| B584 | Fecal E. coli | F | Diseases of the digestive system | University Hospital Brno | 2009 | B2 | mV |
| B596 | Fecal E. coli | M | Diseases of the digestive system | University Hospital Brno | 2009 | D | mV |
| B575 | Fecal E. coli | F | Certain infectious and parasitic diseases | University Hospital Brno | 2009 | B2 | mM |
| B643 | Fecal E. coli | M | Factors influencing health status and contact with health services | University Hospital Brno | 2009 | B1 | mM |
| B369 | Fecal E. coli | M | Certain infectious and parasitic diseases | University Hospital Brno | 2009 | B2 | mL |
| B248 | Fecal E. coli | M | Injury, poisoning and certain other consequences of external causes | University Hospital Brno | 2009 | A | mH47, mM |
| B252 | Fecal E. coli | F | Diseases of the circulatory system | University Hospital Brno | 2009 | D | mH47, mM |
| B259 | Fecal E. coli | M | Certain infectious and parasitic diseases | University Hospital Brno | 2009 | B2 | mH47, mM |
| B263 | Fecal E. coli | F | Diseases of the digestive system | University Hospital Brno | 2009 | B2 | mH47, mM |
| B269 | Fecal E. coli | F | Diseases of the digestive system | University Hospital Brno | 2009 | B2 | mH47, mM |
| B295 | Fecal E. coli | F | Certain infectious and parasitic diseases | University Hospital Brno | 2009 | B2 | mH47, mM |
| B298 | Fecal E. coli | F | Certain infectious and parasitic diseases | University Hospital Brno | 2009 | B2 | mH47, mM |
| B314 | Fecal E. coli | F | Certain infectious and parasitic diseases | University Hospital Brno | 2009 | B2 | mH47, mM |
| B317 | Fecal E. coli | M | Neoplasms | University Hospital Brno | 2009 | B2 | mH47, mM |
| B321 | Fecal E. coli | F | Diseases of the skin and subcutaneous tissue | University Hospital Brno | 2009 | B2 | mH47, mM |
| B326 | Fecal E. coli | F | Diseases of the digestive system | University Hospital Brno | 2009 | B2 | mH47, mM |
| B327 | Fecal E. coli | M | Congenital malformations, deformations and chromosomal abnormalities | University Hospital Brno | 2009 | B2 | mH47, mM |
| B336 | Fecal E. coli | M | Certain infectious and parasitic diseases | University Hospital Brno | 2009 | B2 | mH47, mM |
| B350 | Fecal E. coli | M | Factors influencing health status and contact with health services | University Hospital Brno | 2009 | B2 | mH47, mM |
| B554 | Fecal E. coli | M | Endocrine, nutritional and metabolic diseases | University Hospital Brno | 2009 | B2 | mH47, mM |
| B557 | Fecal E. coli | F | Certain infectious and parasitic diseases | University Hospital Brno | 2009 | B2 | mH47, mM |
| B558 | Fecal E. coli | M | Symptoms, signs and abnormal clinical and laboratory findings, not elsewhere classified | University Hospital Brno | 2009 | B2 | mH47, mM |
| B581 | Fecal E. coli | M | Certain infectious and parasitic diseases | University Hospital Brno | 2009 | B2 | mH47, mM |
| B629 | Fecal E. coli | F | Symptoms, signs and abnormal clinical and laboratory findings, not elsewhere classified | University Hospital Brno | 2009 | B2 | mH47, mM |
| B642 | Fecal E. coli | F | Diseases of the nervous system | University Hospital Brno | 2009 | D | mH47, mM |
| B646 | Fecal E. coli | M | Symptoms, signs and abnormal clinical and laboratory findings, not elsewhere classified | University Hospital Brno | 2009 | D | mH47, mM |
| B652 | Fecal E. coli | M | Diseases of the nervous system | University Hospital Brno | 2009 | B2 | mH47, mM |
| B654 | Fecal E. coli | F | Certain infectious and parasitic diseases | University Hospital Brno | 2009 | B2 | mH47, mM |
| B685 | Fecal E. coli | F | Symptoms, signs and abnormal clinical and laboratory findings, not elsewhere classified | University Hospital Brno | 2009 | B1 | mH47, mM |
| B707 | Fecal E. coli | F | Symptoms, signs and abnormal clinical and laboratory findings, not elsewhere classified | University Hospital Brno | 2009 | D | mH47, mM |
| B709 | Fecal E. coli | F | Endocrine, nutritional and metabolic diseases | University Hospital Brno | 2009 | B2 | mH47, mM |
| B208 | Fecal E. coli | M | Certain infectious and parasitic diseases | University Hospital Brno | 2009 | D | mH47 |
| B223 | Fecal E. coli | F | Endocrine, nutritional and metabolic diseases | University Hospital Brno | 2009 | B2 | mH47 |
| B270 | Fecal E. coli | M | Certain infectious and parasitic diseases | University Hospital Brno | 2009 | D | mH47 |
| B377 | Fecal E. coli | M | Certain infectious and parasitic diseases | University Hospital Brno | 2009 | B2 | mH47 |
| B386 | Fecal E. coli | F | Symptoms, signs and abnormal clinical and laboratory findings, not elsewhere classified | University Hospital Brno | 2009 | B2 | mH47 |
| B392 | Fecal E. coli | M | Certain infectious and parasitic diseases | University Hospital Brno | 2009 | B2 | mH47 |
| B414 | Fecal E. coli | M | Endocrine, nutritional and metabolic diseases | University Hospital Brno | 2009 | B2 | mH47 |
| B428 | Fecal E. coli | M | Endocrine, nutritional and metabolic diseases | University Hospital Brno | 2009 | A | mH47 |
| B432 | Fecal E. coli | M | Endocrine, nutritional and metabolic diseases | University Hospital Brno | 2009 | B2 | mH47 |
| B552 | Fecal E. coli | F | Certain infectious and parasitic diseases | University Hospital Brno | 2009 | A | mH47 |
| B565 | Fecal E. coli | F | Diseases of the respiratory system | University Hospital Brno | 2009 | A | mH47 |
| B605 | Fecal E. coli | M | Certain infectious and parasitic diseases | University Hospital Brno | 2009 | A | mH47 |
| B613 | Fecal E. coli | F | Symptoms, signs and abnormal clinical and laboratory findings, not elsewhere classified | University Hospital Brno | 2009 | B2 | mH47 |
| B638 | Fecal E. coli | M | Diseases of the nervous system | University Hospital Brno | 2009 | B2 | mH47 |
| B678 | Fecal E. coli | M | Certain infectious and parasitic diseases | University Hospital Brno | 2009 | B2 | mH47 |
| B684 | Fecal E. coli | M | Diseases of the digestive system | University Hospital Brno | 2009 | A | mH47 |
| B717 | Fecal E. coli | M | Certain infectious and parasitic diseases | University Hospital Brno | 2009 | B2 | mH47 |
| B212 | Fecal E. coli | F | Certain infectious and parasitic diseases | University Hospital Brno | 2009 | D | mB17, mV |
| B257 | Fecal E. coli | F | Factors influencing health status and contact with health services | University Hospital Brno | 2009 | A | mB17, mV |
| B615 | Fecal E. coli | F | Diseases of the digestive system | University Hospital Brno | 2009 | D | mB17, mV |
| B616 | Fecal E. coli | F | Certain infectious and parasitic diseases | University Hospital Brno | 2009 | D | mB17, mV |
| B364 | Fecal E. coli | M | Symptoms, signs and abnormal clinical and laboratory findings, not elsewhere classified | University Hospital Brno | 2009 | B2 | mB17, mH47, mM |
| B408 | Fecal E. coli | M | Endocrine, nutritional and metabolic diseases | University Hospital Brno | 2009 | B2 | mB17, mH47, mM |
| B308 | Fecal E. coli | M | Diseases of the digestive system | University Hospital Brno | 2009 | D | mB17 |
| B246 | Fecal E. coli | F | Diseases of the digestive system | University Hospital Brno | 2009 | D | mB17 |
| B332 | Fecal E. coli | F | Diseases of the digestive system | University Hospital Brno | 2009 | D | mB17 |
| B334 | Fecal E. coli | F | Diseases of the digestive system | University Hospital Brno | 2009 | D | mB17 |
| B338 | Fecal E. coli | M | Diseases of the digestive system | University Hospital Brno | 2009 | D | mB17 |
| B363 | Fecal E. coli | M | Certain infectious and parasitic diseases | University Hospital Brno | 2009 | D | mB17 |
| B545 | Fecal E. coli | M | Symptoms, signs and abnormal clinical and laboratory findings, not elsewhere classified | University Hospital Brno | 2009 | B2 | mB17 |
| B673 | Fecal E. coli | F | Diseases of the digestive system | University Hospital Brno | 2009 | D | mB17 |
| B339 | Fecal E. coli | F | Diseases of the digestive system | University Hospital Brno | 2009 | D | M, S4, mV |
| B563 | Fecal E. coli | F | Endocrine, nutritional and metabolic diseases | University Hospital Brno | 2009 | A | M, mV |
| B438 | Fecal E. coli | F | Endocrine, nutritional and metabolic diseases | University Hospital Brno | 2009 | B2 | M, mM |
| B645 | Fecal E. coli | F | Certain infectious and parasitic diseases | University Hospital Brno | 2009 | B2 | M, mH47, mM |
| B667 | Fecal E. coli | F | Diseases of the nervous system | University Hospital Brno | 2009 | B2 | M, mH47, mM |
| B635 | Fecal E. coli | F | Diseases of the digestive system | University Hospital Brno | 2009 | B2 | M, mH47 |
| B262 | Fecal E. coli | M | Certain infectious and parasitic diseases | University Hospital Brno | 2009 | B2 | M, Ia |
| B383 | Fecal E. coli | F | Certain infectious and parasitic diseases | University Hospital Brno | 2009 | A | M, B, mV |
| B335 | Fecal E. coli | M | Factors influencing health status and contact with health services | University Hospital Brno | 2009 | B2 | M |
| B379 | Fecal E. coli | M | Certain infectious and parasitic diseases | University Hospital Brno | 2009 | A | M |
| B560 | Fecal E. coli | F | Certain infectious and parasitic diseases | University Hospital Brno | 2009 | A | M |
| B553 | Fecal E. coli | M | Endocrine, nutritional and metabolic diseases | University Hospital Brno | 2009 | D | K, mB17, mH47, mM |
| B397 | Fecal E. coli | F | Certain infectious and parasitic diseases | University Hospital Brno | 2009 | A | K |
| B345 | Fecal E. coli | M | Factors influencing health status and contact with health services | University Hospital Brno | 2009 | D | Js, mB17 |
| B384 | Fecal E. coli | F | Certain infectious and parasitic diseases | University Hospital Brno | 2009 | B2 | Js, mB17 |
| B226 | Fecal E. coli | F | Diseases of the digestive system | University Hospital Brno | 2009 | A | Js |
| B400 | Fecal E. coli | M | Endocrine, nutritional and metabolic diseases | University Hospital Brno | 2009 | D | Js |
| B582 | Fecal E. coli | M | Certain infectious and parasitic diseases | University Hospital Brno | 2009 | D | Js |
| B693 | Fecal E. coli | F | Pregnancy, childbirth and the puerperium | University Hospital Brno | 2009 | A | Js |
| B539 | Fecal E. coli | F | Certain infectious and parasitic diseases | University Hospital Brno | 2009 | B2 | Ib, mV |
| B618 | Fecal E. coli | F | Endocrine, nutritional and metabolic diseases | University Hospital Brno | 2009 | A | Ib, mV |
| B686 | Fecal E. coli | F | Diseases of the digestive system | University Hospital Brno | 2009 | B2 | Ib, mV |
| B714 | Fecal E. coli | F | Diseases of the digestive system | University Hospital Brno | 2009 | B2 | Ib, mV |
| B362 | Fecal E. coli | M | Diseases of the digestive system | University Hospital Brno | 2009 | A | Ib, mM, mV |
| B689 | Fecal E. coli | F | Endocrine, nutritional and metabolic diseases | University Hospital Brno | 2009 | B1 | Ib, mM |
| B241 | Fecal E. coli | F | Certain infectious and parasitic diseases | University Hospital Brno | 2009 | D | Ib, mH47, mM |
| B660 | Fecal E. coli | F | Symptoms, signs and abnormal clinical and laboratory findings, not elsewhere classified | University Hospital Brno | 2009 | B2 | Ib, mH47, mM |
| B354 | Fecal E. coli | F | Symptoms, signs and abnormal clinical and laboratory findings, not elsewhere classified | University Hospital Brno | 2009 | D | Ib, mB17, mM, mV |
| B549 | Fecal E. coli | M | Diseases of the digestive system | University Hospital Brno | 2009 | D | Ib, mB17, mH47 |
| B283 | Fecal E. coli | M | Diseases of the digestive system | University Hospital Brno | 2009 | B1 | Ib, mB17 |
| B713 | Fecal E. coli | M | Diseases of the digestive system | University Hospital Brno | 2009 | A | Ib, M, mH47 |
| B245 | Fecal E. coli | M | Certain infectious and parasitic diseases | University Hospital Brno | 2009 | A | Ib, M |
| B216 | Fecal E. coli | F | Certain infectious and parasitic diseases | University Hospital Brno | 2009 | D | Ib |
| B239 | Fecal E. coli | F | Symptoms, signs and abnormal clinical and laboratory findings, not elsewhere classified | University Hospital Brno | 2009 | B2 | Ib |
| B329 | Fecal E. coli | F | Certain infectious and parasitic diseases | University Hospital Brno | 2009 | B2 | Ib |
| B371 | Fecal E. coli | F | Certain infectious and parasitic diseases | University Hospital Brno | 2009 | A | Ib |
| B571 | Fecal E. coli | M | Endocrine, nutritional and metabolic diseases | University Hospital Brno | 2009 | A | Ib |
| B612 | Fecal E. coli | M | Symptoms, signs and abnormal clinical and laboratory findings, not elsewhere classified | University Hospital Brno | 2009 | B2 | Ib |
| B623 | Fecal E. coli | M | Certain infectious and parasitic diseases | University Hospital Brno | 2009 | A | Ib |
| B546 | Fecal E. coli | M | Certain infectious and parasitic diseases | University Hospital Brno | 2009 | B2 | Ia, N, mV |
| B115 | Fecal E. coli | F | Symptoms, signs and abnormal clinical and laboratory findings, not elsewhere classified | University Hospital Brno | 2009 | B2 | Ia, mV |
| B254 | Fecal E. coli | F | Certain infectious and parasitic diseases | University Hospital Brno | 2009 | A | Ia, mV |
| B288 | Fecal E. coli | F | Symptoms, signs and abnormal clinical and laboratory findings, not elsewhere classified | University Hospital Brno | 2009 | B2 | Ia, mV |
| B346 | Fecal E. coli | M | Factors influencing health status and contact with health services | University Hospital Brno | 2009 | A | Ia, mV |
| B361 | Fecal E. coli | M | Factors influencing health status and contact with health services | University Hospital Brno | 2009 | B2 | Ia, mV |
| B389 | Fecal E. coli | F | Diseases of the digestive system | University Hospital Brno | 2009 | D | Ia, mV |
| B399 | Fecal E. coli | M | Certain infectious and parasitic diseases | University Hospital Brno | 2009 | B2 | Ia, mV |
| B570 | Fecal E. coli | F | Injury, poisoning and certain other consequences of external causes | University Hospital Brno | 2009 | B1 | Ia, mV |
| B573 | Fecal E. coli | M | Certain infectious and parasitic diseases | University Hospital Brno | 2009 | D | Ia, mV |
| B604 | Fecal E. coli | F | Endocrine, nutritional and metabolic diseases | University Hospital Brno | 2009 | B2 | Ia, mV |
| B619 | Fecal E. coli | F | Endocrine, nutritional and metabolic diseases | University Hospital Brno | 2009 | B2 | Ia, mV |
| B624 | Fecal E. coli | F | Diseases of the digestive system | University Hospital Brno | 2009 | B2 | Ia, mV |
| B626 | Fecal E. coli | F | Certain infectious and parasitic diseases | University Hospital Brno | 2009 | D | Ia, mV |
| B665 | Fecal E. coli | F | Endocrine, nutritional and metabolic diseases | University Hospital Brno | 2009 | B2 | Ia, mV |
| B679 | Fecal E. coli | M | Symptoms, signs and abnormal clinical and laboratory findings, not elsewhere classified | University Hospital Brno | 2009 | A | Ia, mV |
| B683 | Fecal E. coli | F | Diseases of the digestive system | University Hospital Brno | 2009 | D | Ia, mV |
| B701 | Fecal E. coli | M | Endocrine, nutritional and metabolic diseases | University Hospital Brno | 2009 | A | Ia, mV |
| B705 | Fecal E. coli | M | Diseases of the blood and blood-forming organs and certain disorders involving the immune mechanism | University Hospital Brno | 2009 | B1 | Ia, mV |
| B351 | Fecal E. coli | M | Factors influencing health status and contact with health services | University Hospital Brno | 2009 | A | Ia, mM, mV |
| B574 | Fecal E. coli | F | Endocrine, nutritional and metabolic diseases | University Hospital Brno | 2009 | B2 | Ia, mM |
| B593 | Fecal E. coli | F | Endocrine, nutritional and metabolic diseases | University Hospital Brno | 2009 | B2 | Ia, mM |
| B598 | Fecal E. coli | M | Symptoms, signs and abnormal clinical and laboratory findings, not elsewhere classified | University Hospital Brno | 2009 | B2 | Ia, mL, mV |
| B600 | Fecal E. coli | M | Diseases of the digestive system | University Hospital Brno | 2009 | B2 | Ia, mH47, mM, mV |
| B544 | Fecal E. coli | F | Factors influencing health status and contact with health services | University Hospital Brno | 2009 | B1 | Ia, mH47 |
| B720 | Fecal E. coli | M | Diseases of the digestive system | University Hospital Brno | 2009 | A | Ia, mC7, mV |
| B711 | Fecal E. coli | F | Symptoms, signs and abnormal clinical and laboratory findings, not elsewhere classified | University Hospital Brno | 2009 | B2 | Ia, mB17, mV |
| B355 | Fecal E. coli | M | Certain infectious and parasitic diseases | University Hospital Brno | 2009 | D | Ia, mB17, mM, mV |
| B360 | Fecal E. coli | M | Factors influencing health status and contact with health services | University Hospital Brno | 2009 | A | Ia, mB17 |
| B433 | Fecal E. coli | M | Endocrine, nutritional and metabolic diseases | University Hospital Brno | 2009 | D | Ia, mB17 |
| B659 | Fecal E. coli | F | Symptoms, signs and abnormal clinical and laboratory findings, not elsewhere classified | University Hospital Brno | 2009 | B2 | Ia, M, mH47, mV |
| B405 | Fecal E. coli | F | Endocrine, nutritional and metabolic diseases | University Hospital Brno | 2009 | B2 | Ia, M, mH47 |
| B634 | Fecal E. coli | M | Certain infectious and parasitic diseases | University Hospital Brno | 2009 | B2 | Ia, M, mH47 |
| B243 | Fecal E. coli | M | Certain infectious and parasitic diseases | University Hospital Brno | 2009 | A | Ia, M |
| B391 | Fecal E. coli | M | Certain infectious and parasitic diseases | University Hospital Brno | 2009 | A | Ia, M |
| B418 | Fecal E. coli | M | Endocrine, nutritional and metabolic diseases | University Hospital Brno | 2009 | A | Ia, M |
| B577 | Fecal E. coli | M | Certain infectious and parasitic diseases | University Hospital Brno | 2009 | B2 | Ia, 5/10, mV |
| B672 | Fecal E. coli | F | Diseases of the digestive system | University Hospital Brno | 2009 | A | Ia, 5/10 |
| B210 | Fecal E. coli | F | Diseases of the digestive system | University Hospital Brno | 2009 | B1 | Ia |
| B256 | Fecal E. coli | M | Certain infectious and parasitic diseases | University Hospital Brno | 2009 | B1 | Ia |
| B267 | Fecal E. coli | M | Symptoms, signs and abnormal clinical and laboratory findings, not elsewhere classified | University Hospital Brno | 2009 | B2 | Ia |
| B278 | Fecal E. coli | M | Symptoms, signs and abnormal clinical and laboratory findings, not elsewhere classified | University Hospital Brno | 2009 | B2 | Ia |
| B296 | Fecal E. coli | M | Diseases of the digestive system | University Hospital Brno | 2009 | A | Ia |
| B310 | Fecal E. coli | M | Endocrine, nutritional and metabolic diseases | University Hospital Brno | 2009 | B2 | Ia |
| B375 | Fecal E. coli | M | Certain infectious and parasitic diseases | University Hospital Brno | 2009 | D | Ia |
| B588 | Fecal E. coli | M | Factors influencing health status and contact with health services | University Hospital Brno | 2009 | B2 | Ia |
| B703 | Fecal E. coli | M | Diseases of the digestive system | University Hospital Brno | 2009 | B2 | Ia |
| B215 | Fecal E. coli | M | Certain infectious and parasitic diseases | University Hospital Brno | 2009 | D | E8, S4, mH47, mM |
| B220 | Fecal E. coli | F | Diseases of the digestive system | University Hospital Brno | 2009 | A | E8, Ib, mV |
| B719 | Fecal E. coli | M | Endocrine, nutritional and metabolic diseases | University Hospital Brno | 2009 | D | E7, Ib, mH47, mM |
| B662 | Fecal E. coli | F | Factors influencing health status and contact with health services | University Hospital Brno | 2009 | A | E7 |
| B359 | Fecal E. coli | F | Diseases of the circulatory system | University Hospital Brno | 2009 | B2 | E6, mH47, mM |
| B551 | Fecal E. coli | F | Diseases of the digestive system | University Hospital Brno | 2009 | B2 | E5, mH47, mM |
| B627 | Fecal E. coli | M | Certain infectious and parasitic diseases | University Hospital Brno | 2009 | D | E3, Ia, K, S4, 5/10, mH47 |
| B550 | Fecal E. coli | M | Endocrine, nutritional and metabolic diseases | University Hospital Brno | 2009 | D | E2, M |
| B221 | Fecal E. coli | M | Certain infectious and parasitic diseases | University Hospital Brno | 2009 | A | E1, mV |
| B393 | Fecal E. coli | M | Diseases of the digestive system | University Hospital Brno | 2009 | D | E1, mM |
| B587 | Fecal E. coli | M | Factors influencing health status and contact with health services | University Hospital Brno | 2009 | B2 | E1, mM |
| B599 | Fecal E. coli | M | Diseases of the digestive system | University Hospital Brno | 2009 | B2 | E1, mH47, mM |
| B537 | Fecal E. coli | F | Factors influencing health status and contact with health services | University Hospital Brno | 2009 | B2 | E1, mH47 |
| B653 | Fecal E. coli | M | Factors influencing health status and contact with health services | University Hospital Brno | 2009 | D | E1, mB17, mM |
| B621 | Fecal E. coli | M | Factors influencing health status and contact with health services | University Hospital Brno | 2009 | A | E1, mB17, mH47 |
| B320 | Fecal E. coli | M | Factors influencing health status and contact with health services | University Hospital Brno | 2009 | D | E1, mB17 |
| B374 | Fecal E. coli | M | Certain infectious and parasitic diseases | University Hospital Brno | 2009 | D | E1, mB17 |
| B381 | Fecal E. coli | M | Endocrine, nutritional and metabolic diseases | University Hospital Brno | 2009 | D | E1, mB17 |
| B591 | Fecal E. coli | M | Diseases of the digestive system | University Hospital Brno | 2009 | B2 | E1, mB17 |
| B268 | Fecal E. coli | M | Diseases of the musculoskeletal system and connective tissue | University Hospital Brno | 2009 | B2 | E1, M, mM |
| B628 | Fecal E. coli | F | Diseases of the nervous system | University Hospital Brno | 2009 | D | E1, Js |
| B380 | Fecal E. coli | M | Certain infectious and parasitic diseases | University Hospital Brno | 2009 | D | E1, Ib, mV |
| B297 | Fecal E. coli | M | Diseases of the circulatory system | University Hospital Brno | 2009 | B2 | E1, Ib, mB17, mH47 |
| B340 | Fecal E. coli | M | Certain infectious and parasitic diseases | University Hospital Brno | 2009 | B2 | E1, Ib, M, mV |
| B352 | Fecal E. coli | M | Certain infectious and parasitic diseases | University Hospital Brno | 2009 | B2 | E1, Ib, M, mM, mV |
| B277 | Fecal E. coli | M | Diseases of the respiratory system | University Hospital Brno | 2009 | B2 | E1, Ib, M, mH47, mM |
| B279 | Fecal E. coli | F | Certain infectious and parasitic diseases | University Hospital Brno | 2009 | B1 | E1, Ib |
| B112 | Fecal E. coli | F | Certain infectious and parasitic diseases | University Hospital Brno | 2009 | A | E1, Ia, mV |
| B117 | Fecal E. coli | F | Neoplasms | University Hospital Brno | 2009 | A | E1, Ia, mV |
| B540 | Fecal E. coli | M | Certain infectious and parasitic diseases | University Hospital Brno | 2009 | B2 | E1, Ia, mV |
| B639 | Fecal E. coli | F | Diseases of the nervous system | University Hospital Brno | 2009 | B2 | E1, Ia, mH47, mM |
| B589 | Fecal E. coli | M | Diseases of the digestive system | University Hospital Brno | 2009 | A | E1, Ia, mB17, mV |
| B276 | Fecal E. coli | M | Diseases of the respiratory system | University Hospital Brno | 2009 | A | E1, Ia, M, mV |
| B284 | Fecal E. coli | M | Certain infectious and parasitic diseases | University Hospital Brno | 2009 | B2 | E1, Ia, M, mV |
| B287 | Fecal E. coli | F | Diseases of the digestive system | University Hospital Brno | 2009 | D | E1, Ia, M, mV |
| B222 | Fecal E. coli | F | Diseases of the digestive system | University Hospital Brno | 2009 | A | E1, Ia, M, mH47, mM |
| B330 | Fecal E. coli | M | Factors influencing health status and contact with health services | University Hospital Brno | 2009 | B2 | E1, Ia, K, mB17, mV |
| B265 | Fecal E. coli | M | Factors influencing health status and contact with health services | University Hospital Brno | 2009 | B1 | E1, E7 |
| B285 | Fecal E. coli | M | Factors influencing health status and contact with health services | University Hospital Brno | 2009 | A | E1, E6, Ia, M, mV |
| B116 | Fecal E. coli | M | Certain infectious and parasitic diseases | University Hospital Brno | 2009 | D | E1 |
| B213 | Fecal E. coli | F | Certain infectious and parasitic diseases | University Hospital Brno | 2009 | D | E1 |
| B280 | Fecal E. coli | F | Diseases of the digestive system | University Hospital Brno | 2009 | D | E1 |
| B348 | Fecal E. coli | M | Factors influencing health status and contact with health services | University Hospital Brno | 2009 | D | E1 |
| B407 | Fecal E. coli | M | Endocrine, nutritional and metabolic diseases | University Hospital Brno | 2009 | B2 | E1 |
| B543 | Fecal E. coli | F | Endocrine, nutritional and metabolic diseases | University Hospital Brno | 2009 | A | E1 |
| B594 | Fecal E. coli | F | Diseases of the digestive system | University Hospital Brno | 2009 | B2 | E1 |
| B669 | Fecal E. coli | M | Diseases of the digestive system | University Hospital Brno | 2009 | B1 | E1 |
| B681 | Fecal E. coli | F | Certain infectious and parasitic diseases | University Hospital Brno | 2009 | A | E1 |
| B688 | Fecal E. coli | F | Diseases of the digestive system | University Hospital Brno | 2009 | D | E1 |
| B695 | Fecal E. coli | F | Diseases of the digestive system | University Hospital Brno | 2009 | D | E1 |
| B715 | Fecal E. coli | M | Endocrine, nutritional and metabolic diseases | University Hospital Brno | 2009 | D | E1 |
| B429 | Fecal E. coli | F | Certain infectious and parasitic diseases | University Hospital Brno | 2009 | B2 | D, mH47, mM |
| B637 | Fecal E. coli | M | Diseases of the respiratory system | University Hospital Brno | 2009 | B2 | B, M, mM |
| B250 | Fecal E. coli | M | Certain infectious and parasitic diseases | University Hospital Brno | 2009 | D | B, M |
| B306 | Fecal E. coli | F | Neoplasms | University Hospital Brno | 2009 | A | B, M |
| B261 | Fecal E. coli | F | Certain infectious and parasitic diseases | University Hospital Brno | 2009 | D | B, K, M, mH47, mM |
| B403 | Fecal E. coli | F | Certain infectious and parasitic diseases | University Hospital Brno | 2009 | D | B, Ia, M |
| B311 | Fecal E. coli | F | Certain infectious and parasitic diseases | University Hospital Brno | 2009 | A | B, E7, Ib, M |
| B312 | Fecal E. coli | F | Diseases of the digestive system | University Hospital Brno | 2009 | A | B, E7, Ib, M |
| B636 | Fecal E. coli | F | Endocrine, nutritional and metabolic diseases | University Hospital Brno | 2009 | D | B, E1, M, mH47 |
| B435 | Fecal E. coli | F | Endocrine, nutritional and metabolic diseases | University Hospital Brno | 2009 | D | B, E1, M |
| B238 | Fecal E. coli | M | Factors influencing health status and contact with health services | University Hospital Brno | 2009 | D | B, E1, K, mH47 |
| B319 | Fecal E. coli | M | Factors influencing health status and contact with health services | University Hospital Brno | 2009 | B2 | B, E1, Ia, mJ25 |
| B315 | Fecal E. coli | M | Certain infectious and parasitic diseases | University Hospital Brno | 2009 | A | B, E1, E6, Ia, M, 5/10 |
| B692 | Fecal E. coli | F | Certain infectious and parasitic diseases | University Hospital Brno | 2009 | B2 | M, mH47, mM |
| A108 | Fecal E. coli | M | Diseases of the digestive system | St. Anne's University Hospital Brno | 2009 | B2 | - |
| A131 | Fecal E. coli | M | Diseases of the digestive system | St. Anne's University Hospital Brno | 2009 | A | - |
| B111 | Fecal E. coli | M | Diseases of the digestive system | University Hospital Brno | 2009 | B2 | - |
| B113 | Fecal E. coli | M | Diseases of the digestive system | University Hospital Brno | 2009 | A | - |
| B114 | Fecal E. coli | M | Diseases of the digestive system | University Hospital Brno | 2009 | B2 | - |
| B129 | Fecal E. coli | F | Mental and behavioural disorders | University Hospital Brno | 2009 | D | - |
| B130 | Fecal E. coli | M | Symptoms, signs and abnormal clinical and laboratory findings, not elsewhere classified | University Hospital Brno | 2009 | B2 | - |
| B211 | Fecal E. coli | F | Endocrine, nutritional and metabolic diseases | University Hospital Brno | 2009 | D | - |
| B214 | Fecal E. coli | M | Certain infectious and parasitic diseases | University Hospital Brno | 2009 | D | - |
| B217 | Fecal E. coli | F | Endocrine, nutritional and metabolic diseases | University Hospital Brno | 2009 | B2 | - |
| B219 | Fecal E. coli | F | Certain infectious and parasitic diseases | University Hospital Brno | 2009 | A | - |
| B227 | Fecal E. coli | M | Certain infectious and parasitic diseases | University Hospital Brno | 2009 | B2 | - |
| B240 | Fecal E. coli | F | Diseases of the digestive system | University Hospital Brno | 2009 | B2 | - |
| B242 | Fecal E. coli | M | Certain infectious and parasitic diseases | University Hospital Brno | 2009 | B2 | - |
| B244 | Fecal E. coli | M | Certain infectious and parasitic diseases | University Hospital Brno | 2009 | D | - |
| B247 | Fecal E. coli | M | Certain infectious and parasitic diseases | University Hospital Brno | 2009 | D | - |
| B249 | Fecal E. coli | M | Symptoms, signs and abnormal clinical and laboratory findings, not elsewhere classified | University Hospital Brno | 2009 | B2 | - |
| B251 | Fecal E. coli | F | Diseases of the digestive system | University Hospital Brno | 2009 | D | - |
| B255 | Fecal E. coli | M | Diseases of the respiratory system | University Hospital Brno | 2009 | B2 | - |
| B258 | Fecal E. coli | F | Diseases of the digestive system | University Hospital Brno | 2009 | A | - |
| B264 | Fecal E. coli | M | Certain infectious and parasitic diseases | University Hospital Brno | 2009 | A | - |
| B266 | Fecal E. coli | M | Factors influencing health status and contact with health services | University Hospital Brno | 2009 | A | - |
| B271 | Fecal E. coli | M | Certain infectious and parasitic diseases | University Hospital Brno | 2009 | B2 | - |
| B272 | Fecal E. coli | M | Certain infectious and parasitic diseases | University Hospital Brno | 2009 | B2 | - |
| B273 | Fecal E. coli | M | Certain infectious and parasitic diseases | University Hospital Brno | 2009 | A | - |
| B274 | Fecal E. coli | F | Neoplasms | University Hospital Brno | 2009 | D | - |
| B281 | Fecal E. coli | M | Diseases of the digestive system | University Hospital Brno | 2009 | B2 | - |
| B286 | Fecal E. coli | M | Factors influencing health status and contact with health services | University Hospital Brno | 2009 | D | - |
| B289 | Fecal E. coli | M | Neoplasms | University Hospital Brno | 2009 | B2 | - |
| B290 | Fecal E. coli | F | Certain infectious and parasitic diseases | University Hospital Brno | 2009 | B2 | - |
| B291 | Fecal E. coli | M | Injury, poisoning and certain other consequences of external causes | University Hospital Brno | 2009 | B2 | - |
| B292 | Fecal E. coli | M | Diseases of the digestive system | University Hospital Brno | 2009 | B2 | - |
| B299 | Fecal E. coli | F | Symptoms, signs and abnormal clinical and laboratory findings, not elsewhere classified | University Hospital Brno | 2009 | B2 | - |
| B300 | Fecal E. coli | F | Diseases of the digestive system | University Hospital Brno | 2009 | A | - |
| B302 | Fecal E. coli | M | Diseases of the digestive system | University Hospital Brno | 2009 | A | - |
| B303 | Fecal E. coli | F | Certain infectious and parasitic diseases | University Hospital Brno | 2009 | A | - |
| B304 | Fecal E. coli | F | Certain infectious and parasitic diseases | University Hospital Brno | 2009 | A | - |
| B305 | Fecal E. coli | M | Certain infectious and parasitic diseases | University Hospital Brno | 2009 | D | - |
| B309 | Fecal E. coli | F | Endocrine, nutritional and metabolic diseases | University Hospital Brno | 2009 | B2 | - |
| B313 | Fecal E. coli | M | Diseases of the musculoskeletal system and connective tissue | University Hospital Brno | 2009 | A | - |
| B316 | Fecal E. coli | F | Symptoms, signs and abnormal clinical and laboratory findings, not elsewhere classified | University Hospital Brno | 2009 | A | - |
| B318 | Fecal E. coli | M | Diseases of the digestive system | University Hospital Brno | 2009 | B2 | - |
| B322 | Fecal E. coli | M | Diseases of the digestive system | University Hospital Brno | 2009 | A | - |
| B323 | Fecal E. coli | F | Diseases of the respiratory system | University Hospital Brno | 2009 | D | - |
| B324 | Fecal E. coli | M | Certain infectious and parasitic diseases | University Hospital Brno | 2009 | B2 | - |
| B325 | Fecal E. coli | M | Symptoms, signs and abnormal clinical and laboratory findings, not elsewhere classified | University Hospital Brno | 2009 | B2 | - |
| B331 | Fecal E. coli | M | Factors influencing health status and contact with health services | University Hospital Brno | 2009 | D | - |
| B333 | Fecal E. coli | M | Diseases of the digestive system | University Hospital Brno | 2009 | A | - |
| B341 | Fecal E. coli | M | Diseases of the circulatory system | University Hospital Brno | 2009 | A | - |
| B342 | Fecal E. coli | M | Symptoms, signs and abnormal clinical and laboratory findings, not elsewhere classified | University Hospital Brno | 2009 | B2 | - |
| B344 | Fecal E. coli | F | Neoplasms | University Hospital Brno | 2009 | D | - |
| B347 | Fecal E. coli | M | Diseases of the digestive system | University Hospital Brno | 2009 | B2 | - |
| B349 | Fecal E. coli | M | Factors influencing health status and contact with health services | University Hospital Brno | 2009 | B2 | - |
| B353 | Fecal E. coli | F | Certain infectious and parasitic diseases | University Hospital Brno | 2009 | A | - |
| B356 | Fecal E. coli | F | Certain infectious and parasitic diseases | University Hospital Brno | 2009 | A | - |
| B357 | Fecal E. coli | F | Injury, poisoning and certain other consequences of external causes | University Hospital Brno | 2009 | D | - |
| B367 | Fecal E. coli | M | Neoplasms | University Hospital Brno | 2009 | B2 | - |
| B370 | Fecal E. coli | M | Certain infectious and parasitic diseases | University Hospital Brno | 2009 | A | - |
| B372 | Fecal E. coli | M | Symptoms, signs and abnormal clinical and laboratory findings, not elsewhere classified | University Hospital Brno | 2009 | A | - |
| B373 | Fecal E. coli | M | Certain infectious and parasitic diseases | University Hospital Brno | 2009 | B2 | - |
| B378 | Fecal E. coli | M | Certain infectious and parasitic diseases | University Hospital Brno | 2009 | D | - |
| B382 | Fecal E. coli | F | Endocrine, nutritional and metabolic diseases | University Hospital Brno | 2009 | B2 | - |
| B385 | Fecal E. coli | M | Certain infectious and parasitic diseases | University Hospital Brno | 2009 | B2 | - |
| B387 | Fecal E. coli | F | Endocrine, nutritional and metabolic diseases | University Hospital Brno | 2009 | D | - |
| B388 | Fecal E. coli | F | Certain infectious and parasitic diseases | University Hospital Brno | 2009 | A | - |
| B390 | Fecal E. coli | M | Certain infectious and parasitic diseases | University Hospital Brno | 2009 | A | - |
| B394 | Fecal E. coli | M | Certain infectious and parasitic diseases | University Hospital Brno | 2009 | B2 | - |
| B395 | Fecal E. coli | F | Certain infectious and parasitic diseases | University Hospital Brno | 2009 | D | - |
| B396 | Fecal E. coli | F | Endocrine, nutritional and metabolic diseases | University Hospital Brno | 2009 | D | - |
| B398 | Fecal E. coli | F | Endocrine, nutritional and metabolic diseases | University Hospital Brno | 2009 | A | - |
| B401 | Fecal E. coli | M | Certain infectious and parasitic diseases | University Hospital Brno | 2009 | D | - |
| B402 | Fecal E. coli | F | Endocrine, nutritional and metabolic diseases | University Hospital Brno | 2009 | B2 | - |
| B404 | Fecal E. coli | M | Endocrine, nutritional and metabolic diseases | University Hospital Brno | 2009 | B2 | - |
| B406 | Fecal E. coli | F | Endocrine, nutritional and metabolic diseases | University Hospital Brno | 2009 | A | - |
| B409 | Fecal E. coli | F | Certain infectious and parasitic diseases | University Hospital Brno | 2009 | A | - |
| B410 | Fecal E. coli | F | Endocrine, nutritional and metabolic diseases | University Hospital Brno | 2009 | A | - |
| B411 | Fecal E. coli | F | Certain infectious and parasitic diseases | University Hospital Brno | 2009 | D | - |
| B412 | Fecal E. coli | M | Certain infectious and parasitic diseases | University Hospital Brno | 2009 | A | - |
| B413 | Fecal E. coli | F | Endocrine, nutritional and metabolic diseases | University Hospital Brno | 2009 | D | - |
| B415 | Fecal E. coli | M | Certain infectious and parasitic diseases | University Hospital Brno | 2009 | A | - |
| B416 | Fecal E. coli | M | Endocrine, nutritional and metabolic diseases | University Hospital Brno | 2009 | A | - |
| B417 | Fecal E. coli | F | Certain infectious and parasitic diseases | University Hospital Brno | 2009 | A | - |
| B419 | Fecal E. coli | F | Certain infectious and parasitic diseases | University Hospital Brno | 2009 | B2 | - |
| B420 | Fecal E. coli | F | Endocrine, nutritional and metabolic diseases | University Hospital Brno | 2009 | A | - |
| B421 | Fecal E. coli | M | Endocrine, nutritional and metabolic diseases | University Hospital Brno | 2009 | B2 | - |
| B422 | Fecal E. coli | M | Certain infectious and parasitic diseases | University Hospital Brno | 2009 | D | - |
| B424 | Fecal E. coli | F | Endocrine, nutritional and metabolic diseases | University Hospital Brno | 2009 | D | - |
| B425 | Fecal E. coli | M | Certain infectious and parasitic diseases | University Hospital Brno | 2009 | B2 | - |
| B426 | Fecal E. coli | F | Certain infectious and parasitic diseases | University Hospital Brno | 2009 | A | - |
| B427 | Fecal E. coli | M | Endocrine, nutritional and metabolic diseases | University Hospital Brno | 2009 | B2 | - |
| B430 | Fecal E. coli | M | Certain infectious and parasitic diseases | University Hospital Brno | 2009 | A | - |
| B431 | Fecal E. coli | M | Certain infectious and parasitic diseases | University Hospital Brno | 2009 | B2 | - |
| B436 | Fecal E. coli | M | Certain infectious and parasitic diseases | University Hospital Brno | 2009 | A | - |
| B437 | Fecal E. coli | M | Certain infectious and parasitic diseases | University Hospital Brno | 2009 | A | - |
| B534 | Fecal E. coli | F | Diseases of the digestive system | University Hospital Brno | 2009 | D | - |
| B535 | Fecal E. coli | M | Certain infectious and parasitic diseases | University Hospital Brno | 2009 | A | - |
| B536 | Fecal E. coli | F | Symptoms, signs and abnormal clinical and laboratory findings, not elsewhere classified | University Hospital Brno | 2009 | A | - |
| B538 | Fecal E. coli | F | Diseases of the digestive system | University Hospital Brno | 2009 | D | - |
| B541 | Fecal E. coli | M | Certain infectious and parasitic diseases | University Hospital Brno | 2009 | D | - |
| B542 | Fecal E. coli | F | Symptoms, signs and abnormal clinical and laboratory findings, not elsewhere classified | University Hospital Brno | 2009 | B2 | - |
| B547 | Fecal E. coli | M | Diseases of the digestive system | University Hospital Brno | 2009 | B2 | - |
| B548 | Fecal E. coli | F | Diseases of the digestive system | University Hospital Brno | 2009 | B2 | - |
| B555 | Fecal E. coli | M | Diseases of the circulatory system | University Hospital Brno | 2009 | B2 | - |
| B556 | Fecal E. coli | F | Endocrine, nutritional and metabolic diseases | University Hospital Brno | 2009 | D | - |
| B559 | Fecal E. coli | M | Certain infectious and parasitic diseases | University Hospital Brno | 2009 | B2 | - |
| B561 | Fecal E. coli | M | Diseases of the digestive system | University Hospital Brno | 2009 | A | - |
| B562 | Fecal E. coli | M | Certain infectious and parasitic diseases | University Hospital Brno | 2009 | A | - |
| B564 | Fecal E. coli | M | Certain infectious and parasitic diseases | University Hospital Brno | 2009 | B2 | - |
| B566 | Fecal E. coli | F | Diseases of the digestive system | University Hospital Brno | 2009 | A | - |
| B567 | Fecal E. coli | M | Symptoms, signs and abnormal clinical and laboratory findings, not elsewhere classified | University Hospital Brno | 2009 | D | - |
| B568 | Fecal E. coli | F | Diseases of the digestive system | University Hospital Brno | 2009 | B2 | - |
| B569 | Fecal E. coli | M | Certain infectious and parasitic diseases | University Hospital Brno | 2009 | B2 | - |
| B572 | Fecal E. coli | F | Diseases of the digestive system | University Hospital Brno | 2009 | B2 | - |
| B576 | Fecal E. coli | M | Diseases of the genitourinary system | University Hospital Brno | 2009 | B1 | - |
| B578 | Fecal E. coli | M | Diseases of the digestive system | University Hospital Brno | 2009 | A | - |
| B579 | Fecal E. coli | M | Factors influencing health status and contact with health services | University Hospital Brno | 2009 | B2 | - |
| B580 | Fecal E. coli | M | Diseases of the circulatory system | University Hospital Brno | 2009 | D | - |
| B583 | Fecal E. coli | M | Diseases of the digestive system | University Hospital Brno | 2009 | A | - |
| B586 | Fecal E. coli | M | Endocrine, nutritional and metabolic diseases | University Hospital Brno | 2009 | B2 | - |
| B590 | Fecal E. coli | F | Factors influencing health status and contact with health services | University Hospital Brno | 2009 | D | - |
| B592 | Fecal E. coli | F | Certain infectious and parasitic diseases | University Hospital Brno | 2009 | D | - |
| B595 | Fecal E. coli | F | Certain infectious and parasitic diseases | University Hospital Brno | 2009 | A | - |
| B597 | Fecal E. coli | M | Endocrine, nutritional and metabolic diseases | University Hospital Brno | 2009 | A | - |
| B601 | Fecal E. coli | M | Certain infectious and parasitic diseases | University Hospital Brno | 2009 | B2 | - |
| B602 | Fecal E. coli | M | Diseases of the digestive system | University Hospital Brno | 2009 | B2 | - |
| B603 | Fecal E. coli | M | Endocrine, nutritional and metabolic diseases | University Hospital Brno | 2009 | B2 | - |
| B606 | Fecal E. coli | M | Diseases of the digestive system | University Hospital Brno | 2009 | B2 | - |
| B607 | Fecal E. coli | M | Factors influencing health status and contact with health services | University Hospital Brno | 2009 | B2 | - |
| B608 | Fecal E. coli | M | Endocrine, nutritional and metabolic diseases | University Hospital Brno | 2009 | B2 | - |
| B609 | Fecal E. coli | M | Endocrine, nutritional and metabolic diseases | University Hospital Brno | 2009 | D | - |
| B610 | Fecal E. coli | M | Endocrine, nutritional and metabolic diseases | University Hospital Brno | 2009 | A | - |
| B611 | Fecal E. coli | M | Diseases of the digestive system | University Hospital Brno | 2009 | B2 | - |
| B614 | Fecal E. coli | F | Diseases of the digestive system | University Hospital Brno | 2009 | D | - |
| B617 | Fecal E. coli | F | Symptoms, signs and abnormal clinical and laboratory findings, not elsewhere classified | University Hospital Brno | 2009 | D | - |
| B620 | Fecal E. coli | M | Certain infectious and parasitic diseases | University Hospital Brno | 2009 | D | - |
| B622 | Fecal E. coli | M | Certain infectious and parasitic diseases | University Hospital Brno | 2009 | A | - |
| B625 | Fecal E. coli | F | Certain infectious and parasitic diseases | University Hospital Brno | 2009 | A | - |
| B630 | Fecal E. coli | M | Diseases of the nervous system | University Hospital Brno | 2009 | A | - |
| B631 | Fecal E. coli | F | Diseases of the digestive system | University Hospital Brno | 2009 | B2 | - |
| B632 | Fecal E. coli | F | Endocrine, nutritional and metabolic diseases | University Hospital Brno | 2009 | B2 | - |
| B633 | Fecal E. coli | F | Certain infectious and parasitic diseases | University Hospital Brno | 2009 | B1 | - |
| B640 | Fecal E. coli | M | Certain infectious and parasitic diseases | University Hospital Brno | 2009 | B1 | - |
| B641 | Fecal E. coli | M | Certain infectious and parasitic diseases | University Hospital Brno | 2009 | D | - |
| B644 | Fecal E. coli | M | Symptoms, signs and abnormal clinical and laboratory findings, not elsewhere classified | University Hospital Brno | 2009 | D | - |
| B648 | Fecal E. coli | M | Factors influencing health status and contact with health services | University Hospital Brno | 2009 | A | - |
| B649 | Fecal E. coli | F | Factors influencing health status and contact with health services | University Hospital Brno | 2009 | B1 | - |
| B650 | Fecal E. coli | F | Symptoms, signs and abnormal clinical and laboratory findings, not elsewhere classified | University Hospital Brno | 2009 | B1 | - |
| B651 | Fecal E. coli | M | Diseases of the digestive system | University Hospital Brno | 2009 | D | - |
| B655 | Fecal E. coli | M | Diseases of the digestive system | University Hospital Brno | 2009 | B2 | - |
| B656 | Fecal E. coli | M | Diseases of the respiratory system | University Hospital Brno | 2009 | A | - |
| B657 | Fecal E. coli | M | Certain infectious and parasitic diseases | University Hospital Brno | 2009 | D | - |
| B658 | Fecal E. coli | M | Certain infectious and parasitic diseases | University Hospital Brno | 2009 | B2 | - |
| B661 | Fecal E. coli | F | Certain infectious and parasitic diseases | University Hospital Brno | 2009 | A | - |
| B663 | Fecal E. coli | F | Diseases of the digestive system | University Hospital Brno | 2009 | D | - |
| B664 | Fecal E. coli | F | Certain infectious and parasitic diseases | University Hospital Brno | 2009 | B2 | - |
| B666 | Fecal E. coli | F | Symptoms, signs and abnormal clinical and laboratory findings, not elsewhere classified | University Hospital Brno | 2009 | B1 | - |
| B668 | Fecal E. coli | F | Diseases of the digestive system | University Hospital Brno | 2009 | D | - |
| B670 | Fecal E. coli | F | Endocrine, nutritional and metabolic diseases | University Hospital Brno | 2009 | A | - |
| B671 | Fecal E. coli | M | Diseases of the digestive system | University Hospital Brno | 2009 | D | - |
| B674 | Fecal E. coli | F | Endocrine, nutritional and metabolic diseases | University Hospital Brno | 2009 | A | - |
| B675 | Fecal E. coli | M | Diseases of the digestive system | University Hospital Brno | 2009 | D | - |
| B676 | Fecal E. coli | M | Factors influencing health status and contact with health services | University Hospital Brno | 2009 | A | - |
| B677 | Fecal E. coli | M | Diseases of the digestive system | University Hospital Brno | 2009 | D | - |
| B680 | Fecal E. coli | F | Endocrine, nutritional and metabolic diseases | University Hospital Brno | 2009 | A | - |
| B682 | Fecal E. coli | M | Symptoms, signs and abnormal clinical and laboratory findings, not elsewhere classified | University Hospital Brno | 2009 | B1 | - |
| B687 | Fecal E. coli | M | Diseases of the digestive system | University Hospital Brno | 2009 | D | - |
| B690 | Fecal E. coli | M | Endocrine, nutritional and metabolic diseases | University Hospital Brno | 2009 | B1 | - |
| B691 | Fecal E. coli | F | Endocrine, nutritional and metabolic diseases | University Hospital Brno | 2009 | D | - |
| B694 | Fecal E. coli | F | Diseases of the digestive system | University Hospital Brno | 2009 | D | - |
| B696 | Fecal E. coli | M | Diseases of the digestive system | University Hospital Brno | 2009 | A | - |
| B697 | Fecal E. coli | F | Endocrine, nutritional and metabolic diseases | University Hospital Brno | 2009 | D | - |
| B698 | Fecal E. coli | F | Endocrine, nutritional and metabolic diseases | University Hospital Brno | 2009 | D | - |
| B699 | Fecal E. coli | M | Endocrine, nutritional and metabolic diseases | University Hospital Brno | 2009 | B1 | - |
| B700 | Fecal E. coli | F | Certain infectious and parasitic diseases | University Hospital Brno | 2009 | D | - |
| B702 | Fecal E. coli | M | Symptoms, signs and abnormal clinical and laboratory findings, not elsewhere classified | University Hospital Brno | 2009 | A | - |
| B706 | Fecal E. coli | F | Diseases of the digestive system | University Hospital Brno | 2009 | B2 | - |
| B708 | Fecal E. coli | F | Diseases of the genitourinary system | University Hospital Brno | 2009 | D | - |
| B710 | Fecal E. coli | F | Certain infectious and parasitic diseases | University Hospital Brno | 2009 | D | - |
| B712 | Fecal E. coli | M | Diseases of the digestive system | University Hospital Brno | 2009 | D | - |
| B716 | Fecal E. coli | M | Endocrine, nutritional and metabolic diseases | University Hospital Brno | 2009 | B1 | - |
| B718 | Fecal E. coli | F | Endocrine, nutritional and metabolic diseases | University Hospital Brno | 2009 | B1 | - |
| B721 | Fecal E. coli | F | Symptoms, signs and abnormal clinical and laboratory findings, not elsewhere classified | University Hospital Brno | 2009 | D | - |
| B740 | Fecal E. coli | F | Endocrine, nutritional and metabolic diseases | University Hospital Brno | 2010 | D | unknown |
| B756 | Fecal E. coli | F | Diseases of the digestive system | University Hospital Brno | 2010 | D | S4, mH47, mM |
| B443 | Fecal E. coli | F | Certain infectious and parasitic diseases | University Hospital Brno | 2010 | B2 | mV |
| B460 | Fecal E. coli | M | Symptoms, signs and abnormal clinical and laboratory findings, not elsewhere classified | University Hospital Brno | 2010 | B2 | mV |
| B466 | Fecal E. coli | M | Certain infectious and parasitic diseases | University Hospital Brno | 2010 | A | mV |
| B501 | Fecal E. coli | F | Certain infectious and parasitic diseases | University Hospital Brno | 2010 | D | mV |
| B532 | Fecal E. coli | M | Certain infectious and parasitic diseases | University Hospital Brno | 2010 | A | mV |
| B731 | Fecal E. coli | M | Neoplasms | University Hospital Brno | 2010 | B1 | mV |
| B743 | Fecal E. coli | M | Endocrine, nutritional and metabolic diseases | University Hospital Brno | 2010 | D | mV |
| B765 | Fecal E. coli | F | Certain infectious and parasitic diseases | University Hospital Brno | 2010 | B1 | mV |
| B822 | Fecal E. coli | M | Certain infectious and parasitic diseases | University Hospital Brno | 2010 | D | mV |
| H15 | Fecal E. coli | M | Neoplasms | University Teaching Hospital Hradec Králove | 2010 | A | mV |
| H19 | Fecal E. coli | F | Neoplasms | University Teaching Hospital Hradec Králove | 2010 | D | mV |
| H20 | Fecal E. coli | F | Neoplasms | University Teaching Hospital Hradec Králove | 2010 | D | mV |
| H21 | Fecal E. coli | F | Neoplasms | University Teaching Hospital Hradec Králove | 2010 | D | mV |
| B473 | Fecal E. coli | F | Injury, poisoning and certain other consequences of external causes | University Hospital Brno | 2010 | B1 | mH47, mM |
| B481 | Fecal E. coli | F | Endocrine, nutritional and metabolic diseases | University Hospital Brno | 2010 | B2 | mH47, mM |
| B487 | Fecal E. coli | M | Certain infectious and parasitic diseases | University Hospital Brno | 2010 | B2 | mH47, mM |
| B489 | Fecal E. coli | M | Endocrine, nutritional and metabolic diseases | University Hospital Brno | 2010 | B2 | mH47, mM |
| B727 | Fecal E. coli | F | Endocrine, nutritional and metabolic diseases | University Hospital Brno | 2010 | B2 | mH47, mM |
| B785 | Fecal E. coli | M | Diseases of the respiratory system | University Hospital Brno | 2010 | B2 | mH47, mM |
| B445 | Fecal E. coli | M | Symptoms, signs and abnormal clinical and laboratory findings, not elsewhere classified | University Hospital Brno | 2010 | B2 | mH47 |
| B454 | Fecal E. coli | M | Endocrine, nutritional and metabolic diseases | University Hospital Brno | 2010 | A | mH47 |
| B465 | Fecal E. coli | F | Diseases of the digestive system | University Hospital Brno | 2010 | B2 | mH47 |
| B478 | Fecal E. coli | F | Endocrine, nutritional and metabolic diseases | University Hospital Brno | 2010 | B2 | mH47 |
| B483 | Fecal E. coli | M | Certain infectious and parasitic diseases | University Hospital Brno | 2010 | B2 | mH47 |
| B498 | Fecal E. coli | M | Diseases of the digestive system | University Hospital Brno | 2010 | B2 | mH47 |
| B500 | Fecal E. coli | F | Factors influencing health status and contact with health services | University Hospital Brno | 2010 | B2 | mH47 |
| B507 | Fecal E. coli | M | Certain infectious and parasitic diseases | University Hospital Brno | 2010 | B2 | mH47 |
| B511 | Fecal E. coli | M | Certain infectious and parasitic diseases | University Hospital Brno | 2010 | B2 | mH47 |
| B522 | Fecal E. coli | M | Certain infectious and parasitic diseases | University Hospital Brno | 2010 | B2 | mH47 |
| B525 | Fecal E. coli | F | Certain infectious and parasitic diseases | University Hospital Brno | 2010 | B2 | mH47 |
| B526 | Fecal E. coli | M | Certain infectious and parasitic diseases | University Hospital Brno | 2010 | B2 | mH47 |
| B793 | Fecal E. coli | F | Certain infectious and parasitic diseases | University Hospital Brno | 2010 | B2 | mH47 |
| B808 | Fecal E. coli | M | Certain infectious and parasitic diseases | University Hospital Brno | 2010 | B2 | mH47 |
| B809 | Fecal E. coli | F | Certain infectious and parasitic diseases | University Hospital Brno | 2010 | B2 | mH47 |
| B810 | Fecal E. coli | M | Certain infectious and parasitic diseases | University Hospital Brno | 2010 | B2 | mH47 |
| B811 | Fecal E. coli | F | Certain infectious and parasitic diseases | University Hospital Brno | 2010 | B2 | mH47 |
| B868 | Fecal E. coli | M | Certain infectious and parasitic diseases | University Hospital Brno | 2010 | B2 | mH47 |
| B869 | Fecal E. coli | F | Certain infectious and parasitic diseases | University Hospital Brno | 2010 | B2 | mH47 |
| H2 | Fecal E. coli | M | Neoplasms | University Teaching Hospital Hradec Králove | 2010 | D | mH47 |
| H5 | Fecal E. coli | M | Neoplasms | University Teaching Hospital Hradec Králove | 2010 | B2 | mH47 |
| H8 | Fecal E. coli | M | Neoplasms | University Teaching Hospital Hradec Králove | 2010 | B2 | mC7, mH47, mM |
| B499 | Fecal E. coli | M | Certain infectious and parasitic diseases | University Hospital Brno | 2010 | B2 | mB17, mH47, mM |
| B837 | Fecal E. coli | M | Certain infectious and parasitic diseases | University Hospital Brno | 2010 | B2 | mB17, mH47 |
| B470 | Fecal E. coli | M | Diseases of the digestive system | University Hospital Brno | 2010 | B2 | mB17 |
| H14 | Fecal E. coli | M | Neoplasms | University Teaching Hospital Hradec Králove | 2010 | D | mB17 |
| B745 | Fecal E. coli | M | Endocrine, nutritional and metabolic diseases | University Hospital Brno | 2010 | B2 | M, mH47, mM |
| B746 | Fecal E. coli | M | Endocrine, nutritional and metabolic diseases | University Hospital Brno | 2010 | B2 | M, mH47, mM |
| B772 | Fecal E. coli | M | Diseases of the circulatory system | University Hospital Brno | 2010 | D | M, mH47, mM |
| B775 | Fecal E. coli | F | Diseases of the genitourinary system | University Hospital Brno | 2010 | B2 | M, mH47, mM |
| B776 | Fecal E. coli | M | Diseases of the blood and blood-forming organs and certain disorders involving the immune mechanism | University Hospital Brno | 2010 | D | M, mH47, mM |
| B777 | Fecal E. coli | M | Neoplasms | University Hospital Brno | 2010 | B2 | M, mH47, mM |
| B780 | Fecal E. coli | F | Diseases of the digestive system | University Hospital Brno | 2010 | B2 | M, mH47, mM |
| B790 | Fecal E. coli | F | Certain infectious and parasitic diseases | University Hospital Brno | 2010 | B2 | M, mH47, mM |
| B475 | Fecal E. coli | M | Certain infectious and parasitic diseases | University Hospital Brno | 2010 | D | M, mH47 |
| B788 | Fecal E. coli | F | Certain infectious and parasitic diseases | University Hospital Brno | 2010 | B2 | M, mB17, mH47, mM |
| B778 | Fecal E. coli | F | Diseases of the respiratory system | University Hospital Brno | 2010 | D | M, mB17, mH47 |
| B804 | Fecal E. coli | F | Diseases of the digestive system | University Hospital Brno | 2010 | B1 | M, B, mV |
| B442 | Fecal E. coli | M | Certain infectious and parasitic diseases | University Hospital Brno | 2010 | B1 | M |
| B451 | Fecal E. coli | F | Endocrine, nutritional and metabolic diseases | University Hospital Brno | 2010 | D | M |
| B797 | Fecal E. coli | M | Diseases of the digestive system | University Hospital Brno | 2010 | D | M |
| B851 | Fecal E. coli | M | Neoplasms | University Hospital Brno | 2010 | B1 | M |
| B864 | Fecal E. coli | M | Certain infectious and parasitic diseases | University Hospital Brno | 2010 | A | M |
| B737 | Fecal E. coli | F | Symptoms, signs and abnormal clinical and laboratory findings, not elsewhere classified | University Hospital Brno | 2010 | B1 | K, S4, mH47, mM |
| B825 | Fecal E. coli | M | Certain infectious and parasitic diseases | University Hospital Brno | 2010 | B2 | K, mH47, mM |
| B826 | Fecal E. coli | M | Certain infectious and parasitic diseases | University Hospital Brno | 2010 | B2 | K, mH47, mM |
| B802 | Fecal E. coli | F | Certain infectious and parasitic diseases | University Hospital Brno | 2010 | B2 | K, mB17, mH47, mM |
| B724 | Fecal E. coli | F | Factors influencing health status and contact with health services | University Hospital Brno | 2010 | B2 | K |
| B805 | Fecal E. coli | M | Certain infectious and parasitic diseases | University Hospital Brno | 2010 | D | Js |
| B846 | Fecal E. coli | F | Certain infectious and parasitic diseases | University Hospital Brno | 2010 | B2 | Js |
| B467 | Fecal E. coli | M | Certain infectious and parasitic diseases | University Hospital Brno | 2010 | A | Ib, mV |
| B734 | Fecal E. coli | M | Certain infectious and parasitic diseases | University Hospital Brno | 2010 | B2 | Ib, mH47, mM |
| B503 | Fecal E. coli | M | Diseases of the genitourinary system | University Hospital Brno | 2010 | A | Ib |
| H13 | Fecal E. coli | M | Neoplasms | University Teaching Hospital Hradec Králove | 2010 | B2 | Ia, S4, mB17, mV |
| B484 | Fecal E. coli | M | Endocrine, nutritional and metabolic diseases | University Hospital Brno | 2010 | D | Ia, N, S4, mV |
| B441 | Fecal E. coli | M | Certain infectious and parasitic diseases | University Hospital Brno | 2010 | B2 | Ia, mV |
| B455 | Fecal E. coli | F | Diseases of the digestive system | University Hospital Brno | 2010 | B2 | Ia, mV |
| B458 | Fecal E. coli | M | Certain infectious and parasitic diseases | University Hospital Brno | 2010 | B2 | Ia, mV |
| B464 | Fecal E. coli | F | Certain infectious and parasitic diseases | University Hospital Brno | 2010 | A | Ia, mV |
| B474 | Fecal E. coli | M | Endocrine, nutritional and metabolic diseases | University Hospital Brno | 2010 | A | Ia, mV |
| B509 | Fecal E. coli | F | Certain infectious and parasitic diseases | University Hospital Brno | 2010 | B2 | Ia, mV |
| B517 | Fecal E. coli | F | Diseases of the digestive system | University Hospital Brno | 2010 | B2 | Ia, mV |
| B723 | Fecal E. coli | F | Endocrine, nutritional and metabolic diseases | University Hospital Brno | 2010 | D | Ia, mV |
| B751 | Fecal E. coli | F | Endocrine, nutritional and metabolic diseases | University Hospital Brno | 2010 | B2 | Ia, mV |
| B814 | Fecal E. coli | M | Neoplasms | University Hospital Brno | 2010 | A | Ia, mV |
| B818 | Fecal E. coli | F | Certain infectious and parasitic diseases | University Hospital Brno | 2010 | D | Ia, mV |
| B841 | Fecal E. coli | M | Certain infectious and parasitic diseases | University Hospital Brno | 2010 | B2 | Ia, mV |
| B856 | Fecal E. coli | M | Neoplasms | University Hospital Brno | 2010 | A | Ia, mV |
| B463 | Fecal E. coli | F | Diseases of the genitourinary system | University Hospital Brno | 2010 | A | Ia, M |
| B516 | Fecal E. coli | F | Pregnancy, childbirth and the puerperium | University Hospital Brno | 2010 | A | Ia, K, mV |
| B471 | Fecal E. coli | M | Endocrine, nutritional and metabolic diseases | University Hospital Brno | 2010 | B2 | Ia, E1, mV |
| B472 | Fecal E. coli | M | Endocrine, nutritional and metabolic diseases | University Hospital Brno | 2010 | B2 | Ia, E1, mV |
| B488 | Fecal E. coli | F | Endocrine, nutritional and metabolic diseases | University Hospital Brno | 2010 | B2 | Ia, E1, mV |
| B527 | Fecal E. coli | F | Certain infectious and parasitic diseases | University Hospital Brno | 2010 | A | Ia, E1, mV |
| B824 | Fecal E. coli | M | Certain infectious and parasitic diseases | University Hospital Brno | 2010 | B2 | Ia, E1, mV |
| B508 | Fecal E. coli | M | Symptoms, signs and abnormal clinical and laboratory findings, not elsewhere classified | University Hospital Brno | 2010 | B2 | Ia, E1 |
| B521 | Fecal E. coli | M | Certain infectious and parasitic diseases | University Hospital Brno | 2010 | D | Ia, E1 |
| B791 | Fecal E. coli | M | Symptoms, signs and abnormal clinical and laboratory findings, not elsewhere classified | University Hospital Brno | 2010 | B2 | Ia, 5/10 |
| B440 | Fecal E. coli | M | Endocrine, nutritional and metabolic diseases | University Hospital Brno | 2010 | B1 | Ia |
| B449 | Fecal E. coli | M | Endocrine, nutritional and metabolic diseases | University Hospital Brno | 2010 | A | Ia |
| B456 | Fecal E. coli | F | Certain infectious and parasitic diseases | University Hospital Brno | 2010 | D | Ia |
| B730 | Fecal E. coli | M | Diseases of the circulatory system | University Hospital Brno | 2010 | B2 | Ia |
| B741 | Fecal E. coli | F | Certain infectious and parasitic diseases | University Hospital Brno | 2010 | D | Ia |
| B755 | Fecal E. coli | M | Certain infectious and parasitic diseases | University Hospital Brno | 2010 | B2 | Ia |
| B757 | Fecal E. coli | M | Diseases of the digestive system | University Hospital Brno | 2010 | D | Ia |
| B758 | Fecal E. coli | F | Certain infectious and parasitic diseases | University Hospital Brno | 2010 | B2 | Ia |
| B773 | Fecal E. coli | M | Diseases of the circulatory system | University Hospital Brno | 2010 | D | Ia |
| B839 | Fecal E. coli | F | Certain infectious and parasitic diseases | University Hospital Brno | 2010 | D | Ia |
| B849 | Fecal E. coli | F | Certain infectious and parasitic diseases | University Hospital Brno | 2010 | D | Ia |
| B852 | Fecal E. coli | F | Certain infectious and parasitic diseases | University Hospital Brno | 2010 | B2 | Ia |
| B861 | Fecal E. coli | F | Certain infectious and parasitic diseases | University Hospital Brno | 2010 | A | Ia |
| B866 | Fecal E. coli | F | Certain infectious and parasitic diseases | University Hospital Brno | 2010 | A | Ia |
| H22 | Fecal E. coli | F | Neoplasms | University Teaching Hospital Hradec Králove | 2010 | B2 | Ia |
| H18 | Fecal E. coli | M | Neoplasms | University Teaching Hospital Hradec Králove | 2010 | B1 | E7, M |
| B515 | Fecal E. coli | F | Certain infectious and parasitic diseases | University Hospital Brno | 2010 | D | E7, Ia, mV |
| B857 | Fecal E. coli | M | Certain infectious and parasitic diseases | University Hospital Brno | 2010 | B2 | E7, Ia, mH47 |
| B506 | Fecal E. coli | F | Certain infectious and parasitic diseases | University Hospital Brno | 2010 | B2 | E7 |
| B832 | Fecal E. coli | F | Certain infectious and parasitic diseases | University Hospital Brno | 2010 | A | E7 |
| B447 | Fecal E. coli | F | Endocrine, nutritional and metabolic diseases | University Hospital Brno | 2010 | B2 | E2, Ia, mB17, mV |
| B842 | Fecal E. coli | M | Certain infectious and parasitic diseases | University Hospital Brno | 2010 | B2 | E2 |
| B531 | Fecal E. coli | F | Diseases of the digestive system | University Hospital Brno | 2010 | D | E1, N, mB17 |
| B492 | Fecal E. coli | M | Certain infectious and parasitic diseases | University Hospital Brno | 2010 | B1 | E1, mV |
| B739 | Fecal E. coli | F | Symptoms, signs and abnormal clinical and laboratory findings, not elsewhere classified | University Hospital Brno | 2010 | B1 | E1, mV |
| B482 | Fecal E. coli | M | Endocrine, nutritional and metabolic diseases | University Hospital Brno | 2010 | B2 | E1, mM |
| B462 | Fecal E. coli | M | Factors influencing health status and contact with health services | University Hospital Brno | 2010 | A | E1, mH47, mM |
| B512 | Fecal E. coli | M | Symptoms, signs and abnormal clinical and laboratory findings, not elsewhere classified | University Hospital Brno | 2010 | B2 | E1, mH47, mM |
| B528 | Fecal E. coli | F | Certain infectious and parasitic diseases | University Hospital Brno | 2010 | B2 | E1, mH47, mM |
| B729 | Fecal E. coli | F | Diseases of the digestive system | University Hospital Brno | 2010 | D | E1, mB17 |
| B733 | Fecal E. coli | F | Certain infectious and parasitic diseases | University Hospital Brno | 2010 | B2 | E1, mB17 |
| B816 | Fecal E. coli | M | Symptoms, signs and abnormal clinical and laboratory findings, not elsewhere classified | University Hospital Brno | 2010 | D | E1, Js, mB17 |
| B867 | Fecal E. coli | F | Certain infectious and parasitic diseases | University Hospital Brno | 2010 | B2 | E1, Js |
| B748 | Fecal E. coli | M | Diseases of the digestive system | University Hospital Brno | 2010 | B1 | E1, Ib, mC7 |
| B476 | Fecal E. coli | M | Diseases of the digestive system | University Hospital Brno | 2010 | D | E1, Ia, M |
| B477 | Fecal E. coli | F | Endocrine, nutritional and metabolic diseases | University Hospital Brno | 2010 | B2 | E1 |
| B524 | Fecal E. coli | M | Symptoms, signs and abnormal clinical and laboratory findings, not elsewhere classified | University Hospital Brno | 2010 | D | E1 |
| B747 | Fecal E. coli | M | Endocrine, nutritional and metabolic diseases | University Hospital Brno | 2010 | B2 | E1 |
| B760 | Fecal E. coli | F | Diseases of the circulatory system | University Hospital Brno | 2010 | B2 | E1 |
| B821 | Fecal E. coli | M | Diseases of the digestive system | University Hospital Brno | 2010 | A | E1 |
| B845 | Fecal E. coli | F | Certain infectious and parasitic diseases | University Hospital Brno | 2010 | A | E1 |
| B865 | Fecal E. coli | F | Diseases of the digestive system | University Hospital Brno | 2010 | A | E1 |
| B799 | Fecal E. coli | M | Certain infectious and parasitic diseases | University Hospital Brno | 2010 | B1 | B, Ia, M |
| B834 | Fecal E. coli | M | Mental and behavioural disorders | University Hospital Brno | 2010 | A | B, Ia |
| B519 | Fecal E. coli | F | Certain infectious and parasitic diseases | University Hospital Brno | 2010 | A | B, E1, M |
| B754 | Fecal E. coli | M | Certain infectious and parasitic diseases | University Hospital Brno | 2010 | B1 | B, E1, M |
| B439 | Fecal E. coli | F | Endocrine, nutritional and metabolic diseases | University Hospital Brno | 2010 | A | - |
| B444 | Fecal E. coli | M | Endocrine, nutritional and metabolic diseases | University Hospital Brno | 2010 | B1 | - |
| B446 | Fecal E. coli | M | Certain infectious and parasitic diseases | University Hospital Brno | 2010 | B1 | - |
| B450 | Fecal E. coli | F | Endocrine, nutritional and metabolic diseases | University Hospital Brno | 2010 | D | - |
| B452 | Fecal E. coli | F | Endocrine, nutritional and metabolic diseases | University Hospital Brno | 2010 | A | - |
| B453 | Fecal E. coli | M | Symptoms, signs and abnormal clinical and laboratory findings, not elsewhere classified | University Hospital Brno | 2010 | D | - |
| B457 | Fecal E. coli | M | Certain infectious and parasitic diseases | University Hospital Brno | 2010 | B1 | - |
| B459 | Fecal E. coli | M | Symptoms, signs and abnormal clinical and laboratory findings, not elsewhere classified | University Hospital Brno | 2010 | A | - |
| B461 | Fecal E. coli | M | Certain infectious and parasitic diseases | University Hospital Brno | 2010 | B2 | - |
| B468 | Fecal E. coli | F | Diseases of the digestive system | University Hospital Brno | 2010 | B2 | - |
| B469 | Fecal E. coli | F | Certain infectious and parasitic diseases | University Hospital Brno | 2010 | B2 | - |
| B479 | Fecal E. coli | F | Endocrine, nutritional and metabolic diseases | University Hospital Brno | 2010 | A | - |
| B480 | Fecal E. coli | M | Endocrine, nutritional and metabolic diseases | University Hospital Brno | 2010 | B1 | - |
| B485 | Fecal E. coli | M | Certain infectious and parasitic diseases | University Hospital Brno | 2010 | A | - |
| B486 | Fecal E. coli | F | Endocrine, nutritional and metabolic diseases | University Hospital Brno | 2010 | B1 | - |
| B490 | Fecal E. coli | M | Diseases of the digestive system | University Hospital Brno | 2010 | D | - |
| B491 | Fecal E. coli | M | Endocrine, nutritional and metabolic diseases | University Hospital Brno | 2010 | B1 | - |
| B493 | Fecal E. coli | M | Endocrine, nutritional and metabolic diseases | University Hospital Brno | 2010 | D | - |
| B494 | Fecal E. coli | M | Certain infectious and parasitic diseases | University Hospital Brno | 2010 | D | - |
| B495 | Fecal E. coli | M | Endocrine, nutritional and metabolic diseases | University Hospital Brno | 2010 | A | - |
| B496 | Fecal E. coli | M | Certain infectious and parasitic diseases | University Hospital Brno | 2010 | B2 | - |
| B497 | Fecal E. coli | M | Certain infectious and parasitic diseases | University Hospital Brno | 2010 | D | - |
| B502 | Fecal E. coli | M | Certain infectious and parasitic diseases | University Hospital Brno | 2010 | B2 | - |
| B504 | Fecal E. coli | F | Certain infectious and parasitic diseases | University Hospital Brno | 2010 | B2 | - |
| B505 | Fecal E. coli | M | Certain infectious and parasitic diseases | University Hospital Brno | 2010 | B2 | - |
| B510 | Fecal E. coli | F | Certain infectious and parasitic diseases | University Hospital Brno | 2010 | A | - |
| B513 | Fecal E. coli | M | Certain infectious and parasitic diseases | University Hospital Brno | 2010 | B2 | - |
| B514 | Fecal E. coli | F | Diseases of the digestive system | University Hospital Brno | 2010 | A | - |
| B518 | Fecal E. coli | F | Certain infectious and parasitic diseases | University Hospital Brno | 2010 | D | - |
| B520 | Fecal E. coli | M | Certain infectious and parasitic diseases | University Hospital Brno | 2010 | B2 | - |
| B523 | Fecal E. coli | M | Certain infectious and parasitic diseases | University Hospital Brno | 2010 | A | - |
| B529 | Fecal E. coli | M | Certain infectious and parasitic diseases | University Hospital Brno | 2010 | D | - |
| B530 | Fecal E. coli | F | Certain infectious and parasitic diseases | University Hospital Brno | 2010 | D | - |
| B722 | Fecal E. coli | M | Endocrine, nutritional and metabolic diseases | University Hospital Brno | 2010 | A | - |
| B725 | Fecal E. coli | M | Diseases of the blood and blood-forming organs and certain disorders involving the immune mechanism | University Hospital Brno | 2010 | D | - |
| B726 | Fecal E. coli | M | Diseases of the blood and blood-forming organs and certain disorders involving the immune mechanism | University Hospital Brno | 2010 | B2 | - |
| B728 | Fecal E. coli | M | Diseases of the respiratory system | University Hospital Brno | 2010 | B2 | - |
| B732 | Fecal E. coli | M | Factors influencing health status and contact with health services | University Hospital Brno | 2010 | D | - |
| B735 | Fecal E. coli | M | Diseases of the digestive system | University Hospital Brno | 2010 | D | - |
| B736 | Fecal E. coli | F | Diseases of the digestive system | University Hospital Brno | 2010 | A | - |
| B738 | Fecal E. coli | M | Neoplasms | University Hospital Brno | 2010 | A | - |
| B742 | Fecal E. coli | F | Endocrine, nutritional and metabolic diseases | University Hospital Brno | 2010 | D | - |
| B744 | Fecal E. coli | F | Endocrine, nutritional and metabolic diseases | University Hospital Brno | 2010 | D | - |
| B749 | Fecal E. coli | M | Endocrine, nutritional and metabolic diseases | University Hospital Brno | 2010 | B2 | - |
| B750 | Fecal E. coli | M | Diseases of the digestive system | University Hospital Brno | 2010 | B2 | - |
| B752 | Fecal E. coli | F | Certain infectious and parasitic diseases | University Hospital Brno | 2010 | D | - |
| B753 | Fecal E. coli | F | Symptoms, signs and abnormal clinical and laboratory findings, not elsewhere classified | University Hospital Brno | 2010 | A | - |
| B759 | Fecal E. coli | M | Certain infectious and parasitic diseases | University Hospital Brno | 2010 | D | - |
| B761 | Fecal E. coli | M | Diseases of the circulatory system | University Hospital Brno | 2010 | A | - |
| B762 | Fecal E. coli | F | Certain infectious and parasitic diseases | University Hospital Brno | 2010 | B1 | - |
| B763 | Fecal E. coli | M | Symptoms, signs and abnormal clinical and laboratory findings, not elsewhere classified | University Hospital Brno | 2010 | D | - |
| B764 | Fecal E. coli | M | Certain infectious and parasitic diseases | University Hospital Brno | 2010 | A | - |
| B766 | Fecal E. coli | F | Certain infectious and parasitic diseases | University Hospital Brno | 2010 | D | - |
| B774 | Fecal E. coli | F | Certain infectious and parasitic diseases | University Hospital Brno | 2010 | B2 | - |
| B779 | Fecal E. coli | M | Factors influencing health status and contact with health services | University Hospital Brno | 2010 | A | - |
| B781 | Fecal E. coli | M | Certain infectious and parasitic diseases | University Hospital Brno | 2010 | B2 | - |
| B782 | Fecal E. coli | F | Symptoms, signs and abnormal clinical and laboratory findings, not elsewhere classified | University Hospital Brno | 2010 | B1 | - |
| B783 | Fecal E. coli | F | Neoplasms | University Hospital Brno | 2010 | B2 | - |
| B784 | Fecal E. coli | F | Certain infectious and parasitic diseases | University Hospital Brno | 2010 | B2 | - |
| B786 | Fecal E. coli | M | Diseases of the blood and blood-forming organs and certain disorders involving the immune mechanism | University Hospital Brno | 2010 | D | - |
| B787 | Fecal E. coli | F | Certain infectious and parasitic diseases | University Hospital Brno | 2010 | D | - |
| B789 | Fecal E. coli | M | Diseases of the circulatory system | University Hospital Brno | 2010 | B2 | - |
| B794 | Fecal E. coli | M | Certain infectious and parasitic diseases | University Hospital Brno | 2010 | B2 | - |
| B795 | Fecal E. coli | F | Certain infectious and parasitic diseases | University Hospital Brno | 2010 | A | - |
| B796 | Fecal E. coli | F | Certain infectious and parasitic diseases | University Hospital Brno | 2010 | B2 | - |
| B798 | Fecal E. coli | M | Certain infectious and parasitic diseases | University Hospital Brno | 2010 | A | - |
| B800 | Fecal E. coli | M | Certain infectious and parasitic diseases | University Hospital Brno | 2010 | D | - |
| B801 | Fecal E. coli | M | Symptoms, signs and abnormal clinical and laboratory findings, not elsewhere classified | University Hospital Brno | 2010 | D | - |
| B803 | Fecal E. coli | F | Certain infectious and parasitic diseases | University Hospital Brno | 2010 | B2 | - |
| B806 | Fecal E. coli | F | Diseases of the digestive system | University Hospital Brno | 2010 | A | - |
| B807 | Fecal E. coli | F | Symptoms, signs and abnormal clinical and laboratory findings, not elsewhere classified | University Hospital Brno | 2010 | B2 | - |
| B812 | Fecal E. coli | F | Certain infectious and parasitic diseases | University Hospital Brno | 2010 | A | - |
| B813 | Fecal E. coli | M | Certain infectious and parasitic diseases | University Hospital Brno | 2010 | B2 | - |
| B815 | Fecal E. coli | F | Certain infectious and parasitic diseases | University Hospital Brno | 2010 | D | - |
| B817 | Fecal E. coli | M | Certain infectious and parasitic diseases | University Hospital Brno | 2010 | A | - |
| B819 | Fecal E. coli | F | Certain infectious and parasitic diseases | University Hospital Brno | 2010 | D | - |
| B820 | Fecal E. coli | M | Diseases of the digestive system | University Hospital Brno | 2010 | A | - |
| B823 | Fecal E. coli | M | Factors influencing health status and contact with health services | University Hospital Brno | 2010 | A | - |
| B827 | Fecal E. coli | M | Diseases of the digestive system | University Hospital Brno | 2010 | D | - |
| B828 | Fecal E. coli | F | Factors influencing health status and contact with health services | University Hospital Brno | 2010 | A | - |
| B829 | Fecal E. coli | M | Certain infectious and parasitic diseases | University Hospital Brno | 2010 | D | - |
| B830 | Fecal E. coli | F | Factors influencing health status and contact with health services | University Hospital Brno | 2010 | A | - |
| B831 | Fecal E. coli | M | Diseases of the digestive system | University Hospital Brno | 2010 | B2 | - |
| B833 | Fecal E. coli | F | Certain infectious and parasitic diseases | University Hospital Brno | 2010 | D | - |
| B835 | Fecal E. coli | F | Certain infectious and parasitic diseases | University Hospital Brno | 2010 | B2 | - |
| B836 | Fecal E. coli | M | Certain infectious and parasitic diseases | University Hospital Brno | 2010 | A | - |
| B838 | Fecal E. coli | F | Certain infectious and parasitic diseases | University Hospital Brno | 2010 | B2 | - |
| B840 | Fecal E. coli | M | Certain infectious and parasitic diseases | University Hospital Brno | 2010 | D | - |
| B843 | Fecal E. coli | M | Certain infectious and parasitic diseases | University Hospital Brno | 2010 | A | - |
| B844 | Fecal E. coli | M | Certain infectious and parasitic diseases | University Hospital Brno | 2010 | A | - |
| B847 | Fecal E. coli | M | Diseases of the respiratory system | University Hospital Brno | 2010 | B2 | - |
| B848 | Fecal E. coli | M | Certain infectious and parasitic diseases | University Hospital Brno | 2010 | B2 | - |
| B850 | Fecal E. coli | M | Certain infectious and parasitic diseases | University Hospital Brno | 2010 | A | - |
| B853 | Fecal E. coli | F | Certain infectious and parasitic diseases | University Hospital Brno | 2010 | D | - |
| B854 | Fecal E. coli | F | Certain infectious and parasitic diseases | University Hospital Brno | 2010 | B2 | - |
| B855 | Fecal E. coli | M | Certain infectious and parasitic diseases | University Hospital Brno | 2010 | A | - |
| B858 | Fecal E. coli | M | Certain infectious and parasitic diseases | University Hospital Brno | 2010 | D | - |
| B859 | Fecal E. coli | F | Symptoms, signs and abnormal clinical and laboratory findings, not elsewhere classified | University Hospital Brno | 2010 | B2 | - |
| B860 | Fecal E. coli | M | Certain infectious and parasitic diseases | University Hospital Brno | 2010 | B2 | - |
| B862 | Fecal E. coli | F | Certain infectious and parasitic diseases | University Hospital Brno | 2010 | B2 | - |
| B863 | Fecal E. coli | M | Symptoms, signs and abnormal clinical and laboratory findings, not elsewhere classified | University Hospital Brno | 2010 | A | - |
| B870 | Fecal E. coli | M | Certain infectious and parasitic diseases | University Hospital Brno | 2010 | D | - |
| B871 | Fecal E. coli | M | Certain infectious and parasitic diseases | University Hospital Brno | 2010 | A | - |
| H1 | Fecal E. coli | F | Neoplasms | University Teaching Hospital Hradec Králove | 2010 | A | - |
| H10 | Fecal E. coli | F | Neoplasms | University Teaching Hospital Hradec Králove | 2010 | A | - |
| H11 | Fecal E. coli | F | Neoplasms | University Teaching Hospital Hradec Králove | 2010 | D | - |
| H12 | Fecal E. coli | F | Neoplasms | University Teaching Hospital Hradec Králove | 2010 | A | - |
| H16 | Fecal E. coli | M | Neoplasms | University Teaching Hospital Hradec Králove | 2010 | A | - |
| H17 | Fecal E. coli | M | Neoplasms | University Teaching Hospital Hradec Králove | 2010 | B2 | - |
| H3 | Fecal E. coli | M | Neoplasms | University Teaching Hospital Hradec Králove | 2010 | B2 | - |
| H4 | Fecal E. coli | M | Neoplasms | University Teaching Hospital Hradec Králove | 2010 | B2 | - |
| H6 | Fecal E. coli | F | Neoplasms | University Teaching Hospital Hradec Králove | 2010 | A | - |
| H7 | Fecal E. coli | F | Neoplasms | University Teaching Hospital Hradec Králove | 2010 | A | - |
| H9 | Fecal E. coli | M | Neoplasms | University Teaching Hospital Hradec Králove | 2010 | A | - |
| H38 | Fecal E. coli | F | Neoplasms | University Teaching Hospital Hradec Králove | 2011 | A | S4, mH47, mM |
| H33 | Fecal E. coli | F | Neoplasms | University Teaching Hospital Hradec Králove | 2011 | A | mV |
| H29 | Fecal E. coli | M | Neoplasms | University Teaching Hospital Hradec Králove | 2011 | B2 | mM |
| H23 | Fecal E. coli | M | Neoplasms | University Teaching Hospital Hradec Králove | 2011 | B2 | mH47, mM |
| H28 | Fecal E. coli | F | Neoplasms | University Teaching Hospital Hradec Králove | 2011 | B2 | mH47, mM |
| H39 | Fecal E. coli | F | Neoplasms | University Teaching Hospital Hradec Králove | 2011 | B2 | mB17, mH47, mM, mV |
| H31 | Fecal E. coli | M | Neoplasms | University Teaching Hospital Hradec Králove | 2011 | B2 | K |
| H24 | Fecal E. coli | F | Neoplasms | University Teaching Hospital Hradec Králove | 2011 | A | E1, Ia, mV |
| H36 | Fecal E. coli | F | Neoplasms | University Teaching Hospital Hradec Králove | 2011 | A | E1, Ia |
| H34 | Fecal E. coli | M | Neoplasms | University Teaching Hospital Hradec Králove | 2011 | B2 | B, M, mH47, mM |
| H25 | Fecal E. coli | F | Neoplasms | University Teaching Hospital Hradec Králove | 2011 | A | B, E1, M |
| H26 | Fecal E. coli | M | Neoplasms | University Teaching Hospital Hradec Králove | 2011 | D | - |
| H27 | Fecal E. coli | F | Neoplasms | University Teaching Hospital Hradec Králove | 2011 | D | - |
| H30 | Fecal E. coli | M | Neoplasms | University Teaching Hospital Hradec Králove | 2011 | D | - |
| H32 | Fecal E. coli | M | Neoplasms | University Teaching Hospital Hradec Králove | 2011 | D | - |
| H35 | Fecal E. coli | M | Neoplasms | University Teaching Hospital Hradec Králove | 2011 | B2 | - |
| H37 | Fecal E. coli | F | Neoplasms | University Teaching Hospital Hradec Králove | 2011 | A | - |
| H49 | Fecal E. coli | F | Neoplasms | University Teaching Hospital Hradec Králove | 2013 | B2 | mV |
| H75 | Fecal E. coli | M | Neoplasms | University Teaching Hospital Hradec Králove | 2013 | D | mV |
| H60 | Fecal E. coli | F | Neoplasms | University Teaching Hospital Hradec Králove | 2013 | B2 | mM |
| H96 | Fecal E. coli | F | Neoplasms | University Teaching Hospital Hradec Králove | 2013 | B2 | mM |
| H97 | Fecal E. coli | F | Neoplasms | University Teaching Hospital Hradec Králove | 2013 | B2 | mM |
| H89 | Fecal E. coli | M | Neoplasms | University Teaching Hospital Hradec Králove | 2013 | B2 | mH47, mV |
| H46 | Fecal E. coli | F | Neoplasms | University Teaching Hospital Hradec Králove | 2013 | B2 | mH47, mM |
| H98 | Fecal E. coli | M | Neoplasms | University Teaching Hospital Hradec Králove | 2013 | B2 | mH47, mM |
| H76 | Fecal E. coli | M | Neoplasms | University Teaching Hospital Hradec Králove | 2013 | B2 | mH47, mL, mM |
| H77 | Fecal E. coli | M | Neoplasms | University Teaching Hospital Hradec Králove | 2013 | D | mH47, mL, mM |
| H69 | Fecal E. coli | F | Neoplasms | University Teaching Hospital Hradec Králove | 2013 | A | mH47 |
| H93 | Fecal E. coli | M | Neoplasms | University Teaching Hospital Hradec Králove | 2013 | D | mC7, mV |
| H94 | Fecal E. coli | M | Neoplasms | University Teaching Hospital Hradec Králove | 2013 | D | mC7, mV |
| H84 | Fecal E. coli | F | Neoplasms | University Teaching Hospital Hradec Králove | 2013 | A | mB17 |
| H85 | Fecal E. coli | F | Neoplasms | University Teaching Hospital Hradec Králove | 2013 | A | mB17 |
| H90 | Fecal E. coli | F | Neoplasms | University Teaching Hospital Hradec Králove | 2013 | D | mB17 |
| H50 | Fecal E. coli | M | Neoplasms | University Teaching Hospital Hradec Králove | 2013 | B2 | Js |
| H87 | Fecal E. coli | F | Neoplasms | University Teaching Hospital Hradec Králove | 2013 | B1 | Ib, mV |
| H41 | Fecal E. coli | F | Neoplasms | University Teaching Hospital Hradec Králove | 2013 | B2 | Ia, mV |
| H67 | Fecal E. coli | F | Neoplasms | University Teaching Hospital Hradec Králove | 2013 | B2 | Ia, mV |
| H101 | Fecal E. coli | M | Neoplasms | University Teaching Hospital Hradec Králove | 2013 | D | Ia |
| H88 | Fecal E. coli | F | Neoplasms | University Teaching Hospital Hradec Králove | 2013 | A | Ia |
| H92 | Fecal E. coli | M | Neoplasms | University Teaching Hospital Hradec Králove | 2013 | A | E7, M |
| H42 | Fecal E. coli | F | Neoplasms | University Teaching Hospital Hradec Králove | 2013 | B1 | E7 |
| H61 | Fecal E. coli | M | Neoplasms | University Teaching Hospital Hradec Králove | 2013 | A | E1, mB17, mH47 |
| H44 | Fecal E. coli | M | Neoplasms | University Teaching Hospital Hradec Králove | 2013 | A | E1, K, mH47 |
| H91 | Fecal E. coli | F | Neoplasms | University Teaching Hospital Hradec Králove | 2013 | D | E1, Js, mM |
| H100 | Fecal E. coli | M | Neoplasms | University Teaching Hospital Hradec Králove | 2013 | A | E1 |
| H66 | Fecal E. coli | M | Neoplasms | University Teaching Hospital Hradec Králove | 2013 | A | E1 |
| H70 | Fecal E. coli | F | Neoplasms | University Teaching Hospital Hradec Králove | 2013 | A | E1 |
| H68 | Fecal E. coli | M | Neoplasms | University Teaching Hospital Hradec Králove | 2013 | B2 | B, mH47, mV |
| H45 | Fecal E. coli | M | Neoplasms | University Teaching Hospital Hradec Králove | 2013 | B2 | B, M, mV |
| H102 | Fecal E. coli | M | Neoplasms | University Teaching Hospital Hradec Králove | 2013 | D | - |
| H40 | Fecal E. coli | M | Neoplasms | University Teaching Hospital Hradec Králove | 2013 | A | - |
| H43 | Fecal E. coli | M | Neoplasms | University Teaching Hospital Hradec Králove | 2013 | B2 | - |
| H47 | Fecal E. coli | M | Neoplasms | University Teaching Hospital Hradec Králove | 2013 | B2 | - |
| H48 | Fecal E. coli | F | Neoplasms | University Teaching Hospital Hradec Králove | 2013 | B2 | - |
| H51 | Fecal E. coli | F | Neoplasms | University Teaching Hospital Hradec Králove | 2013 | A | - |
| H52 | Fecal E. coli | F | Neoplasms | University Teaching Hospital Hradec Králove | 2013 | D | - |
| H53 | Fecal E. coli | F | Neoplasms | University Teaching Hospital Hradec Králove | 2013 | B2 | - |
| H54 | Fecal E. coli | F | Neoplasms | University Teaching Hospital Hradec Králove | 2013 | B2 | - |
| H55 | Fecal E. coli | M | Neoplasms | University Teaching Hospital Hradec Králove | 2013 | B2 | - |
| H56 | Fecal E. coli | F | Neoplasms | University Teaching Hospital Hradec Králove | 2013 | B2 | - |
| H57 | Fecal E. coli | F | Neoplasms | University Teaching Hospital Hradec Králove | 2013 | D | - |
| H58 | Fecal E. coli | F | Neoplasms | University Teaching Hospital Hradec Králove | 2013 | B2 | - |
| H59 | Fecal E. coli | M | Neoplasms | University Teaching Hospital Hradec Králove | 2013 | D | - |
| H62 | Fecal E. coli | M | Neoplasms | University Teaching Hospital Hradec Králove | 2013 | A | - |
| H63 | Fecal E. coli | M | Neoplasms | University Teaching Hospital Hradec Králove | 2013 | A | - |
| H64 | Fecal E. coli | M | Neoplasms | University Teaching Hospital Hradec Králove | 2013 | B1 | - |
| H65 | Fecal E. coli | M | Neoplasms | University Teaching Hospital Hradec Králove | 2013 | A | - |
| H71 | Fecal E. coli | M | Neoplasms | University Teaching Hospital Hradec Králove | 2013 | A | - |
| H72 | Fecal E. coli | F | Neoplasms | University Teaching Hospital Hradec Králove | 2013 | B1 | - |
| H73 | Fecal E. coli | M | Neoplasms | University Teaching Hospital Hradec Králove | 2013 | B2 | - |
| H74 | Fecal E. coli | M | Neoplasms | University Teaching Hospital Hradec Králove | 2013 | A | - |
| H78 | Fecal E. coli | F | Neoplasms | University Teaching Hospital Hradec Králove | 2013 | A | - |
| H79 | Fecal E. coli | F | Neoplasms | University Teaching Hospital Hradec Králove | 2013 | D | - |
| H80 | Fecal E. coli | M | Neoplasms | University Teaching Hospital Hradec Králove | 2013 | A | - |
| H81 | Fecal E. coli | M | Neoplasms | University Teaching Hospital Hradec Králove | 2013 | D | - |
| H82 | Fecal E. coli | F | Neoplasms | University Teaching Hospital Hradec Králove | 2013 | B2 | - |
| H83 | Fecal E. coli | M | Neoplasms | University Teaching Hospital Hradec Králove | 2013 | A | - |
| H86 | Fecal E. coli | F | Neoplasms | University Teaching Hospital Hradec Králove | 2013 | A | - |
| H95 | Fecal E. coli | F | Neoplasms | University Teaching Hospital Hradec Králove | 2013 | B2 | - |
| H99 | Fecal E. coli | F | Neoplasms | University Teaching Hospital Hradec Králove | 2013 | D | - |
